# Resolving the structure of the E_1_ state of Mo nitrogenase through Mo and Fe K-edge EXAFS and QM/MM calculations†
†Electronic supplementary information (ESI) available. See DOI: 10.1039/c9sc02187f


**DOI:** 10.1039/c9sc02187f

**Published:** 2019-09-04

**Authors:** Casey Van Stappen, Albert Thor Thorhallsson, Laure Decamps, Ragnar Bjornsson, Serena DeBeer

**Affiliations:** a Max-Planck Institute for Chemical Energy Conversion , Stiftstrasse 34-36 , 45470 Mülheim an der Ruhr , NRW , Germany . Email: serena.debeer@cec.mpg.de ; Email: ragnar.bjornsson@cec.mpg.de

## Abstract

Biological nitrogen fixation is predominately accomplished through Mo nitrogenase, which utilizes a complex MoFe_7_S_9_C catalytic cluster to reduce N_2_ to NH_3_. This cluster requires the accumulation of three to four reducing equivalents prior to binding N_2_; however, despite decades of research, the intermediate states formed prior to N_2_ binding are still poorly understood. Herein, we use Mo and Fe K-edge X-ray absorption spectroscopy and QM/MM calculations to investigate the nature of the E_1_ state, which is formed following the addition of the first reducing equivalent to Mo nitrogenase. By analyzing the extended X-ray absorption fine structure (EXAFS) region, we provide structural insight into the changes that occur in the metal clusters of the protein when forming the E_1_ state, and use these metrics to assess a variety of possible models of the E_1_ state. The combination of our experimental and theoretical results supports that formation of E_1_ involves an Fe-centered reduction combined with the protonation of a belt-sulfide of the cluster. Hence, these results provide critical experiment and computational insight into the mechanism of this important enzyme.

## Abstract 2

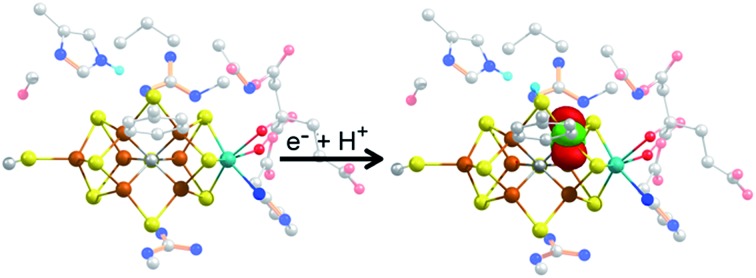
The FeMoco cluster of Mo nitrogenase undergoes minor distortions upon reduction to E_1_, supporting iron-based reduction and belt sulfide protonation.

## Introduction

Mo nitrogenase (Mo N_2_ase) performs a crucial step in the biogeochemical nitrogen cycle, reducing N_2_ to two molecules of NH_3_. This enzyme utilizes a two-component system comprised of the active-site containing MoFe protein and reducing Fe protein (FeP). The subunits of the dimeric FeP are connected through a 4Fe–4S cluster, which serves to transfer electrons to the MoFe protein. The MoFe protein is composed of an α_2_β_2_ heterotetramer, where each αβ subunit contains two large iron–sulfur clusters, namely the 8Fe–7S P-cluster, which serves as an electron transfer site, and the MoFe_7_S_9_C cluster, commonly referred to as the FeMo-cofactor (FeMoco), which serves as the catalytic active site for N_2_ reduction.^1^,^2^

**Scheme 1:**
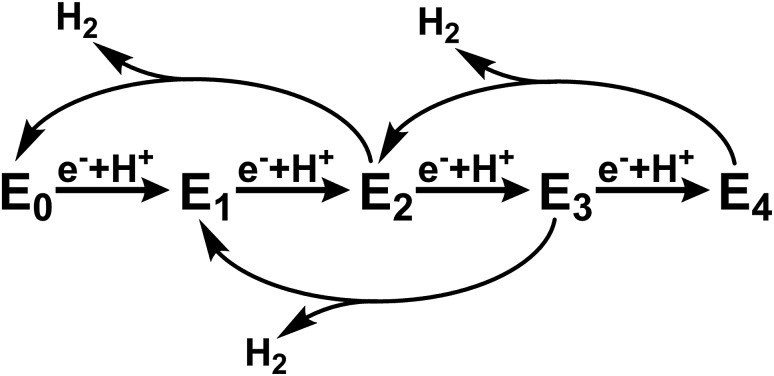
Abbreviated version of the Lowe–Thorneley cycle emphasizing the states formed in the absence of N_2_ substrate.^6^–^9^

During native turnover, electrons are transferred to FeMoco in a discreet, step-wise fashion. This is initiated by the binding of reduced ATP-bound Fe protein to MoFe, which induces a conformationally gated one-electron transfer from the P-cluster to FeMoco (forming P^1+^) that is in turn followed by a rapid one-electron transfer from FeP^red^ to the P-cluster in a “deficit spending” electron transfer process.^3^,^4^ This is followed by hydrolysis of ATP to ADP, the release of two phosphate ions (P_i_), and subsequent dissociation of oxidized FeP.^5^ This process is repeated a total of four times to initiate binding of N_2_ to the FeMoco cluster and an additional four times to subsequently reduce N_2_ to 2NH_3_. In the absence of N_2_ or other possible substrates, no more than four e^–^/H^+^ equivalents have been observed to accumulate, and release of H_2_ leads to relaxation of the cluster to its resting state (Scheme 1).

This cycle is commonly interpreted in terms of the pioneering work of Lowe and Thorneley, who described the kinetic relationships between the proposed catalytic intermediates E_*n*_, in which *n* represents the number of electrons delivered to the FeMoco cofactors from FeP.^6^–^9^ Since each electron transfer step is coupled with the transfer of a proton, H_2_ may be produced as a side product from any state between *n* = 2 and 4. To avoid producing excessive amounts of H_2_, the rate of electron transfer must be fast relative to the rate of H_2_ production. The electron transfer rate, and in turn population of the various states E_*n*_ prior to N_2_ binding at the E_4_ state, may be controlled by varying the ratio of the two protein components. Based on this scheme, it is possible to selectively populate only the E_0_ and E_1_ states under conditions in which the rate of H_2_ production from the E_2_ state (reported as up to ∼250 s^–1^)^6^ is faster than the rate of E_2_ formation. This is enabled by use of a large ratio of MoFe : FeP (for example, 25 : 1 or greater) which results in a low rate of electron-transfer. Meanwhile, in the absence of N_2_, sufficiently low ratios of MoFe : FeP (less than 1 : 4) should result in the near complete population of E_4_.^10^

Recently, significant advances have been made in our understanding of how FeMoco may be capable of accumulating and storing protons and electrons in E_2_ and E_4_ as a result of ^1^H and ^57^Fe ENDOR studies. These studies support the formation of hydride species in both E_2_ and E_4_, which may serve to level the redox potential of the cluster.^11^–^13^ However, the E_1_ and E_3_ states have remained uncharacterized by EPR methods due in part to their integer spin. In addition, the inability to isolate pure intermediates during turnover has limited the application of other spectroscopic techniques. To-date, there are no reports characterizing the electronic or geometric structure of E_3_, and only three which have investigated E_1_.^14^–^16^

One of these three investigations of E_1_ utilized Mo and Fe K-edge EXAFS to structurally characterize this state. Interestingly, significant contractions in the Mo–Fe and Mo–O/N distances (–0.06 Å and –0.07 Å, respectively) were reported relative to the E_0_ state. Similarly, a contraction of ∼0.05 Å was found in the average short Fe–Fe distances.^14^ Meanwhile, our recent studies have revealed that formation of E_1_ involves an Fe-centered reduction, and that Mo remains redox innocent.^15^ These results appear confounding, as such large contractions in bond distances are non-intuitive for a system undergoing either reduction (in the case of Fe) or no effective oxidation state change (in the case of Mo). In addition, a more recent EXAFS study examining the structural changes of FeMoco upon binding of CO during native turnover (using similar electron-flux conditions as the previous EXAFS study) did not report any bond contractions at Mo when moving from the resting to CO bound state; instead, only minor elongations of the Mo–O/N and Mo–Fe distances were found.^17^

**Fig. 1:**
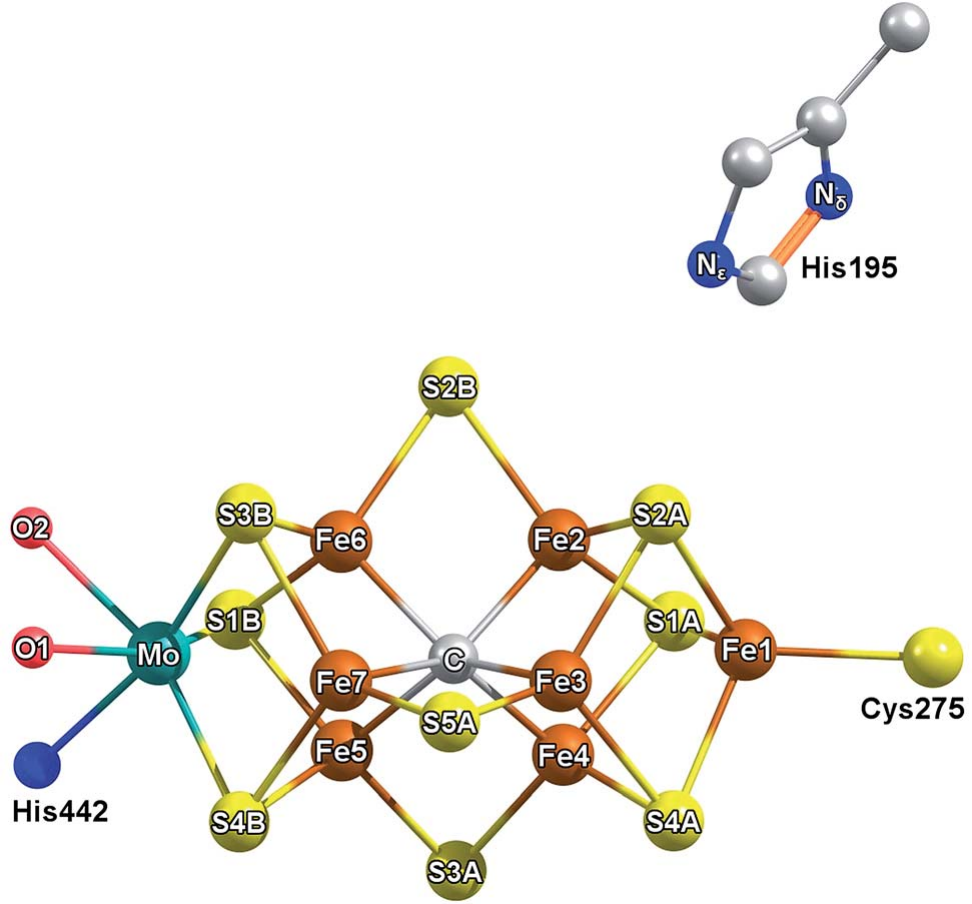
Labelling scheme used presently to describe the iron and sulfur sites of FeMoco and their relative orientation to homocitrate (red) and residues His442, His195. The coordinating O of homocitrate are labeled as O1 for the carboxylic oxygen and O2 for the hydroxyl group.

What is the precise nature of the E_1_ state? On the basis of previous Mo and Fe K-edge XAS and ^57^Fe Mössbauer studies, it is clear that Fe is reduced in E_1_.^15^,^16^ However, whether E_1_ additionally involves protonation of a sulfide or the formation of an iron-hydride (either end-on or bridging) at the FeMoco cluster remains unclear. The permutation space for possible species is quite high, as any of the 7 irons or 9 sulfur sites could hypothetically be protonated. Previous computational investigations have found the bridging sulfides (often referred to as S2B, S3A, S5A) to be the most basic,^18^,^19^ while S3B has also been suggested as an initial sulfur protonation site (see Fig. 1 for labelling).^19^–^23^ The situation is further complicated by whether the Mo-bound homocitrate is protonated; while computational studies have suggested a protonated hydroxyl group of homocitrate in E_0_ based on crystal structure comparison,^24^,^25^ this information is not currently available for the E_1_ state.

To shed light on the nature of the E_1_ state, we have reinvestigated the Fe and Mo EXAFS of Mo N_2_ase to elucidate the structural changes which occur at Fe and Mo. The revised experimental changes in this state are further used as a metric for QM/MM DFT calculations to provide further insight into the possible nature of this intermediate, particularly regarding sulfide-protonation *vs.* iron-hydride formation.

## Experimental details

### Materials and protein purifications

All reagents were obtained from Sigma-Aldrich (St. Louis, MO) or Fisher Scientific (Fair Lawn, NJ) and were used without further purification. Argon and dinitrogen gases were purchased from Westfalen and passed through an activated copper catalyst to remove any traces of dioxygen before use. Mo nitrogenase of *Azotobacter vinelandii* was produced as described previously.^26^ Protein concentrations were determined by Lowry assay.^27^ The purities of these proteins were >95% based on SDS-PAGE analysis with Coomassie staining. All manipulation of proteins and buffers were performed under an Ar atmosphere.

### Preparation of freeze-quenched nitrogenase XAS samples

All XAS samples were prepared under an Ar atmosphere and contained final concentrations of 300 μM MoFe, 12 μM FeP, 50 mM Tris, 200 mM NaCl, 2.5 mM MgCl_2_, and 10 mM sodium dithionite at pH = 7.5. Turnover state samples were prepared by the addition of an “activating” buffer solution containing 60 mM creatine phosphate, 30 mM MgATP, and 50 units per ml creatine phosphokinase (50% of total final volume) to a solution containing 600 μM MoFe and 24 μM FeP, which was then freeze quenched in liquid N_2_ after being allowed to react for 120 s. Turnover samples contained final concentrations of 30 mM creatine phosphate, 15 mM MgATP, and 25 units per ml creatine kinase. Although FeP is present in both resting and turnover samples, the Fe present from FeP only accounts for approximately 0.5% of the total Fe in these samples as a 25 : 1 [MoFe] : [FeP] ratio was employed.

### EPR measurements

EPR measurements were performed to quantify the reduction of resting MoFe, and are detailed in Section S1 of the ESI.† An average of 50% reduction in the *S* = 3/2 E_0_ signal was found for turnover samples, based upon both relative intensity of the *g*_1_ feature at *g* = 4.3 and spin-integration area (Fig. S1-1†). No *S* = 1/2 or 5/2 signals associated with the one electron oxidized P^1+^ state of the P-cluster were observed.

### X-ray spectroscopic measurements

X-ray absorption measurements of intact nitrogenase MoFe and FeP were obtained at the 9–3 beamline of the Stanford Synchrotron Radiation Lightsource (SSRL). The SPEAR storage ring operated at 3.0 GeV in top-off mode with a ∼500 mA ring current. A liquid N_2_ cooled double-crystal monochromator with Si(220) crystals at *φ* = 0° was used to select the incoming X-ray energy with an intrinsic resolution (Δ*E*/*E*) of ∼0.6 × 10^–4^, and a Rh-coated mirror was used for harmonic rejection. The X-ray beam size was 1 × 4 mm^2^ (*V* × *H*) at the sample position. A liquid helium flow cryostat was used to maintain a ∼20 K sample environment in order to minimize radiation damage and maintain an inert sample environment. Fluorescence measurements were recorded using a Canberra 100-element Ge monolith solid-state detector. Prior to measurements, each sample was checked for signs of radiation damage by performing subsequent five minute scans over the same sample spot. These tests showed the MoFe protein was stable under X-ray irradiation at the Mo K-edge for >90 minutes, and >70 minutes at the Fe K-edge.

For Mo XAS measurements, the energy of the incoming X-rays was calibrated by simultaneous measurement of a Mo foil and assigning the energy of the maximum of the white line to 20 016.4 eV. Full XAS scans were collected by scanning the incident energy from 19 780 to 21 142 eV. All Fe XAS scans were collected by scanning the incident energy from 6882 to 8093 eV, and calibrated by simultaneous measurement of an Fe foil, with the first inflection point set to 7111.2 eV.

### PFY-XAS data processing & statistical analysis

In all experiments, individual scans were normalized to the incident photon flux and averaged using the program Athena from the software package Demeter.^28^ Further processing of spectra including background subtraction and normalization was also performed using Athena,^28^ following standard protocols for X-ray spectroscopy described below. Statistical analysis of XAS measurements was performed by normalization of individual scans based on edge area, followed by a calculation of the standard deviation (eqn (1)),1
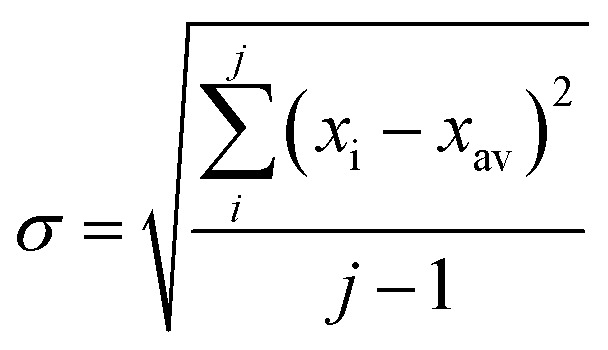
where *σ* is the standard deviation, *x*_i_ is an individual scan, *x*_av_ is the average over all scans, and *j* is the total number of scans. Errors provided for difference spectra were propagated using eqn (2),2
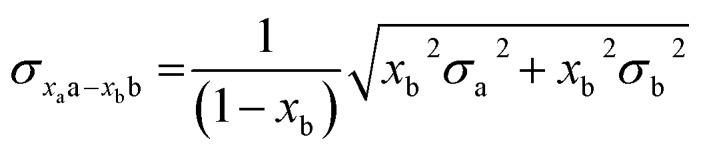
where *σ*_*x*_a_a–*x*_b_b_ is the standard deviation of the renormalized spectrum generated by subtraction of fraction *x*_b_ of spectrum “b” from spectrum “a”. In all cases, *x*_a_ = 1. Where difference spectra are presented, in which *x*_b_ = 1, eqn (2) simplifies to:3
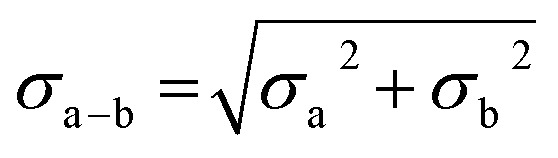



EXAFS fitting was performed using the program Artemis, also of the software package Demeter.^28^ Possible scattering paths for the EXAFS models were initially determined using FEFF 7.0 in combination with a recent high-resolution crystal structure (PDB ID ; 3U7Q).^29^ The structural parameters *R* (bond distance) and *σ*^2^ (bond variance) were allowed to vary during fitting refinement for all measured data. A value of *S*_0_^2^ of 1 was used in fitting the Mo EXAFS, and 0.9 in fitting the Fe EXAFS. A single Δ*E*_0_ parameter was assigned to all scattering paths at a given edge, and allowed to vary in the refinement of the resting state Mo and Fe K-edge EXAFS. This refined value of Δ*E*_0_ was subsequently fixed at these best fit values for further analysis of the turnover and E_1_ EXAFS data. The Mo and Fe spectra of E_1_ were generated by multiplying the normalized E_0_ spectrum by 0.50 (quantified by EPR based on the 50% reduction of the E_0_*S* = 3/2 signal during turnover, Fig. S1-1†), and subtracting it from the normalized turnover (E_0_ + E_1_) spectrum; the result was renormalized by multiplying by two.

Root-mean-square deviations of the changes in distances of the calculations relative to those determined experimentally were also determined. To do so, we first define the change in distance, Δ*R*, of path *i*:4
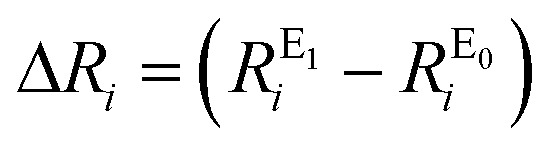



We can further define the deviation of the calculated change in distance from that determined experimentally as:

5 Δ*R*calc–exp*i* = (Δ*R*calc*i* – Δ*R*exp*i*)

where Δ*R*calc*i* is the calculated change in a given scattering path, and Δ*R*exp*i* is the experimentally observed change in a giving scattering distance, E_1_–E_0_. The root mean-square deviation can then be calculated as the normalized sum-of-squares.6
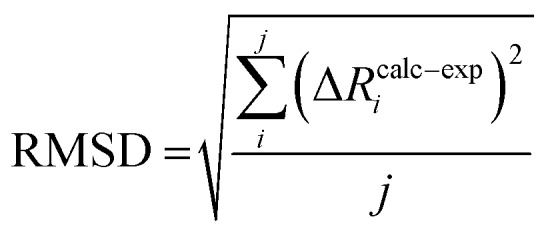
where *j* is the total number of paths. To account for the uncertainty in the experimentally determined distances, the RMSD can be weighted using a normal distribution:7
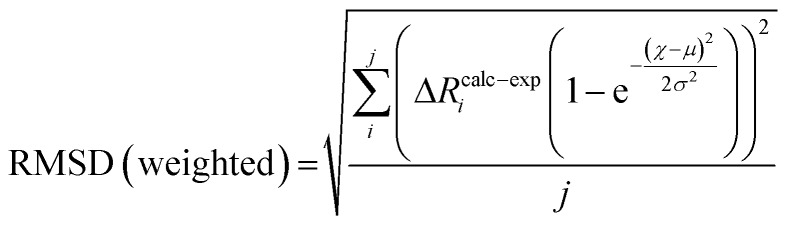
where *χ* is the calculated mean, *μ* is the experimental mean, and *σ* is the experimental standard deviation.

### Mo data processing

Background subtraction and normalization of the averaged Mo EXAFS spectrum was performed using a linear regression for the pre-edge region of 19 910–19 947 eV, and a quadratic polynomial regression for the post-edge region of 20 157–20 807 eV. Data were splined from *k* = 0–17.2 Å^–1^ using an *R*-background of 1.0 Å and *k*-weight of 2. The resulting spectrum was *k*^3^-weighted to emphasize the high importance of data at higher *k*. A *k*-range of 2–16.5 Å^–1^ was used for the curve fitting analysis of E_0_ and E_0_ + E_1_ giving a maximum resolution of Δ*R* = 0.108 Å. A *k*-range of 2–16 Å^–1^ was used in the curve fitting analysis of the E_1_ spectrum, giving a maximum resolution of Δ*R* = 0.112 Å. All data were fit in *R*-space using an *R*-range of 1.5 to 3.5 Å. Due to the already considerable complexity of the EXAFS of the MoFe protein, fitting was limited to include only single scattering paths. No smoothing was used at any point in any of the data processing.

### Fe data processing

The Fe EXAFS was processed in a similar fashion to that of the Mo EXAFS. Background subtraction and normalization was performed using a linear regression for the pre-edge region of 6990–7005 eV, and a quadratic polynomial regression for the post-edge region of 7160–8200 eV. Data were splined from *k* = 0–15.9 Å^–1^ using an *R*-background of 1.0 and *k*-weight of 2. A *k*-range of 2–15.8 Å^–1^ was used in the curve fitting analysis of E_0_ and E_0_ + E_1_ to provide a maximum resolution of Δ*R* = 0.114 Å. Meanwhile, a reduced *k*-range of 2–13 Å^–1^ was used for E_1_, with a maximum Δ*R* = 0.143 Å. All data were fit in *R*-space using an *R*-range of 1.5 to 4.0 Å. Due to the already considerable complexity of the EXAFS of the MoFe protein, fitting was limited to include only single scattering paths. In the case of the split short Fe–Fe scattering path model, the Debye–Waller factors of the two short Fe–Fe scatterers are fixed to be equivalent to one another as the scatterers are the same identity and in a similar environment. This was done to minimize the number of free parameters. No smoothing was used at any point in any of the data processing.

### QM/MM

The QM/MM models for E_1_ were based on our previous model for the E_0_ resting state.^24^ It is a spherical QM/MM model (42 Å radius) centered on the carbide of FeMoco. In the QM/MM geometry optimizations, the active region consists of 1000 atoms and a QM region of 133 atoms. All QM/MM calculations were performed in Chemshell version 3.7 (ref. 30) using the built-in MM code DL_POLY^31^ with the CHARMM36 (ref. 32 and 33) forcefield and ORCA version 4.0 (ref. 34) as QM code. The QM region contains the FeMoco cofactor, singly protonated homocitrate (unless otherwise mentioned) and the sidechains surrounding the cluster which are believed to be critical to describing the coordination, asymmetry, and hydrogen-bonding environment around FeMoco. This includes residues directly coordinating FeMoco (His442, Cys275), neighboring charged residues (Arg96, Arg359), those capable of participating in hydrogen bonding (His195, Gln191, Ser278, Glu380), as well as spatially close residues (Val70, Phe381). For further details, see Fig. S6.4-2 of the ESI.† All QM/MM calculations used electrostatic embedding, and the link atom scheme with charge-shifting was used to terminate the QM–MM border as implemented in Chemshell.^30^ The QM calculations used the TPSSh^35^ hybrid density functional (previously found to describe the cofactor well^24^), a ZORA scalar relativistic Hamiltonian,^36^,^37^ the relativistically recontracted def2-TZVP^38^–^40^ basis set on all metal and sulfur atoms, as well as on the homocitrate, carbide, and two H atoms (def2-SVP on other atoms) and the D3 dispersion correction with Becke–Johnson damping.^41^–^43^ The RIJCOSX approximation^44^–^47^ was used to speed up computation of Coulomb and HF Exchange integrals. Different electronic states of the cofactor for both the E_0_ model and the different E_1_ models were explored by the use of broken-symmetry DFT methodology, as used in previous studies by us.^24^,^48^,^49^ This involves flipping the spin on different Fe atoms starting from a high-spin *M*_S_ = 35/2 determinant (for E_0_) or *M*_S_ = 34/2 (for E_1_) and then converging to antiferromagnetic low-spin states with a particular *M*_S_ value. This results in different electronic states (broken-symmetry states), that we label according to which Fe ions are “spin-down”. The three lowest energy states (for both E_0_ and E_1_) correspond to the “BS7” category by Lovell, *et al.*^50^ Section 6.1 of the ESI† provides more detail on the nature of these different states.

## Results

### XANES

The normalized Mo and Fe K-edge XANES spectra of MoFe in the resting (E_0_), turnover (E_0_ + E_1_), and E_1_ states are provided in Fig. S5-1 and S5-2.† We have previously reported on the Mo and Fe K-edges of MoFe in the E_1_ state in detail.^15^ The changes observed presently at both Mo and Fe are consistent with our previous findings. No significant shifts are found at the Mo K-edge moving from E_0_ to E_1_, indicating no change in either oxidation state or coordination at Mo. Meanwhile, at the Fe K-edge, reduced pre-edge and edge intensities are followed by a concomitant increase in intensity at the white-line when moving from E_0_ to E_1_, indicative of an Fe-centered reduction.^15^,^48^

### Mo EXAFS

**Fig. 2:**
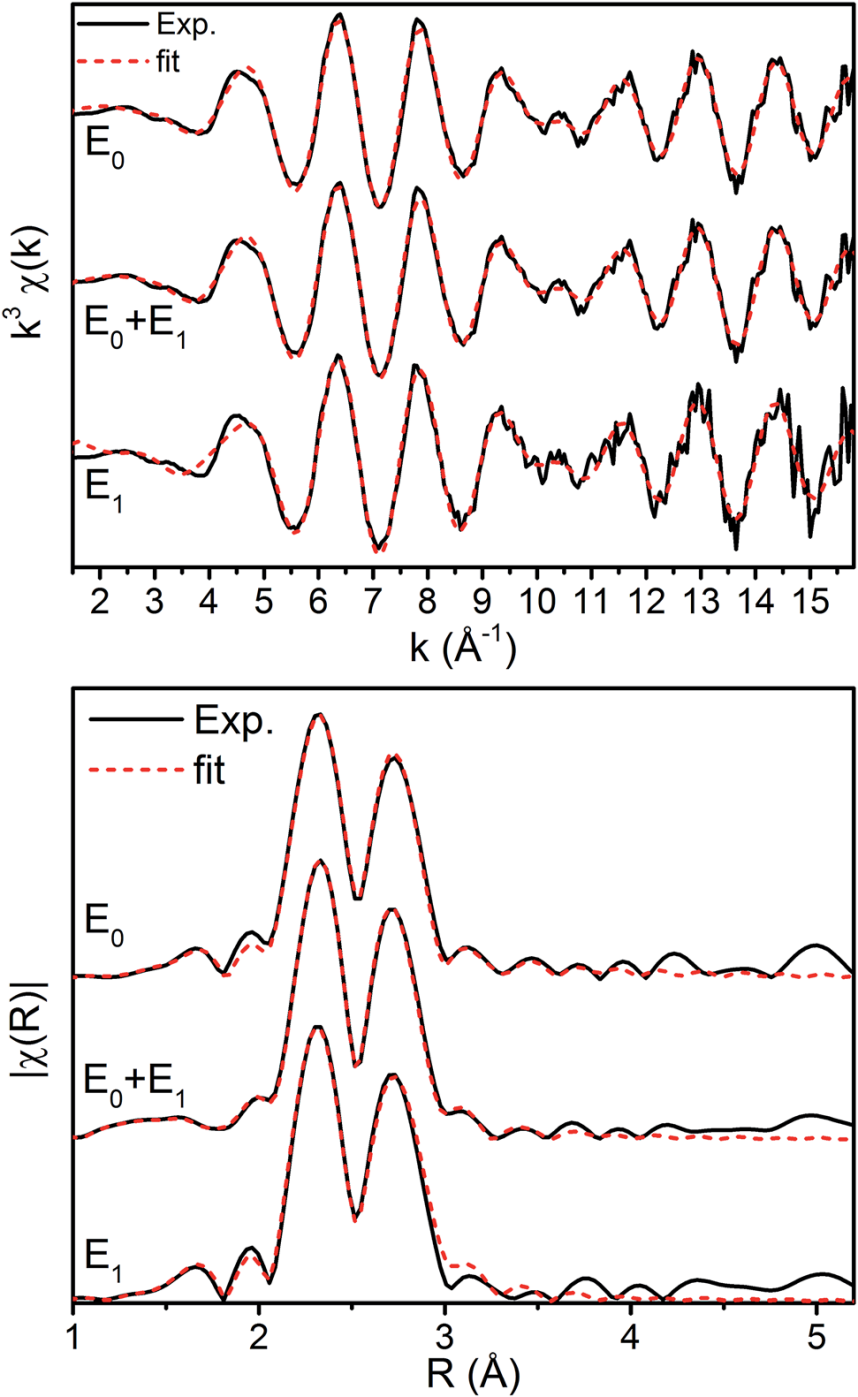
(Top) *k*^3^-Weighted and (bottom) FT-spectra of the Mo EXAFS of resting (E_0_), turnover (E_0_ + E_1_), and E_1_ state. Solid black lines denote experimental spectra while red dashed lines indicate best fits using the 3 scattering path model. The FT-spectra are phase corrected for the Mo–S scattering path.

The *k*^3^-weighted Mo EXAFS of the resting E_0_, turnover (E_0_ + E_1_), and E_1_ states of MoFe are shown in Fig. 2 along with their corresponding Fourier transforms (FT), while fit parameters are provided in Table 1.

**Table 1:** Summary of Mo EXAFS fitting parameters for the resting (E_0_), turnover (E_0_ + E_1_), and E_1_ spectra using both 3 scattering path (Mo–O/N, Mo–S, and Mo–Fe) and 4 scattering path (Mo–O, Mo–N, Mo–S, and Mo–Fe) models. Standard errors are provided for *σ*^2^ and *R* in parentheses as determined from the fitting procedure

Mo FEFF fits, *E*_0_ = 20 013.895 eV							
Sample	Path = 3	*N*	*S*_0_^2^	*σ* ^2^ (10^–3^ Å^2^)	*R* (Å)	*k* (Å^–1^)	*R*-Factor
Resting	O/N	3	1	4.20 (1.01)	2.217 (0.009)	2–16.5 min. Δ*R* 0.108	0.0061
	S	3		2.42 (0.30)	2.362 (0.003)		
	Fe-short	3		3.25 (0.18)	2.689 (0.002)		
Turnover (E_0_ + E_1_)	O/N	3	1	3.52 (1.01)	2.221 (0.009)	2–16.5 min. Δ*R* 0.108	0.0065
	S	3		2.57 (0.36)	2.361 (0.004)		
	Fe-short	3		3.24 (0.19)	2.688 (0.002)		
Turnover (E_1_)	O/N	3	1	2.34 (1.31)	2.221 (0.010)	2–16 min. Δ*R* 0.112	0.0117
	S	3		2.81 (0.68)	2.365 (0.005)		
	Fe-short	3		3.16 (0.29)	2.697 (0.003)		

Sample	Path = 4	*N*	*S*_0_^2^	*σ* ^2^ (10^–3^ Å^2^)	*R* (Å)	*k* (Å^–1^)	*R*-Factor
Resting	O	2	1	2.36 (0.67)	2.198 (0.007)	1.6–16.4 min. Δ*R* 0.106	0.0024
	N	1		1.72 (1.09)	2.303 (0.011)		
	S	3		2.51 (0.20)	2.365 (0.004)		
	Fe-short	3		3.18 (0.11)	2.693 (0.001)		
Turnover (E_0_ + E_1_)	O	2	1	2.53 (0.68)	2.197 (0.006)	1.6–16.4 min. Δ*R* 0.106	0.0024
	N	1		2.01 (1.35)	2.309 (0.011)		
	S	3		2.24 (0.16)	2.361 (0.002)		
	Fe-short	3		3.16 (0.11)	2.688 (0.001)		
Turnover (E_1_)	O	2	1	0.93 (0.77)	2.202 (0.011)	1.6–16 min. Δ*R* 0.109	0.0108
	N	1		3.00 (0.02)	2.302 (0.006)		
	S	3		2.57 (0.52)	2.367 (0.006)		
	Fe-short	3		3.18 (0.26)	2.697 (0.003)		

No significant changes are observed from a simple comparison of the resting and turnover states. A slight broadening in the |FT| of E_1_ is seen, though this is likely due to the increase in noise which naturally results from the spectra subtraction. All spectra exhibit two clear shells (∼2.3 and 2.7 Å), as well as a small feature ∼5 Å, previously reported as a long-distance Mo–Fe scatterer.^17^

**Fig. 3:**
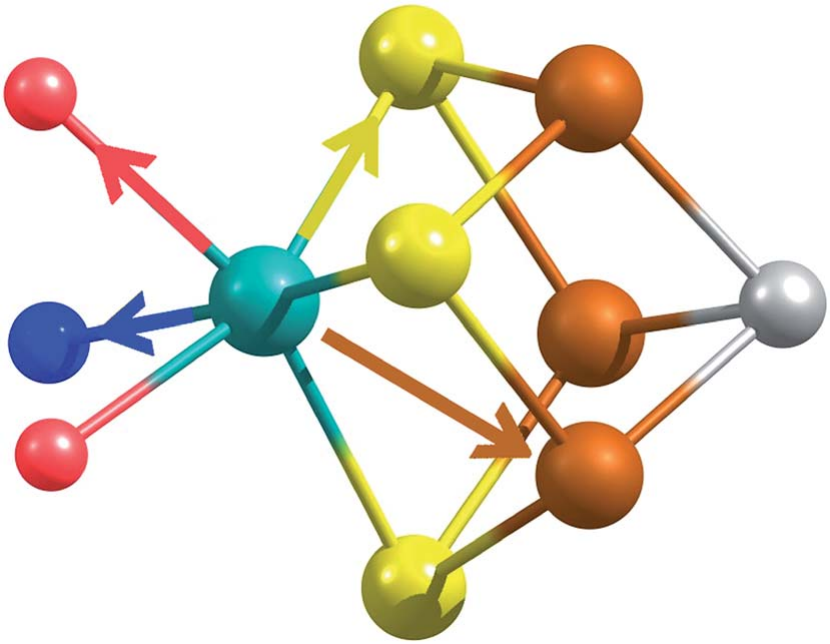
Short single-scattering paths of Mo in FeMoco. Model based on coordinates obtained from the XRD structure, PDB ID: 3U7Q.^29^ These include the Mo–N (blue), Mo–O (red), Mo–S (yellow), and Mo–Fe (rust) distances.

**Fig. 4:**
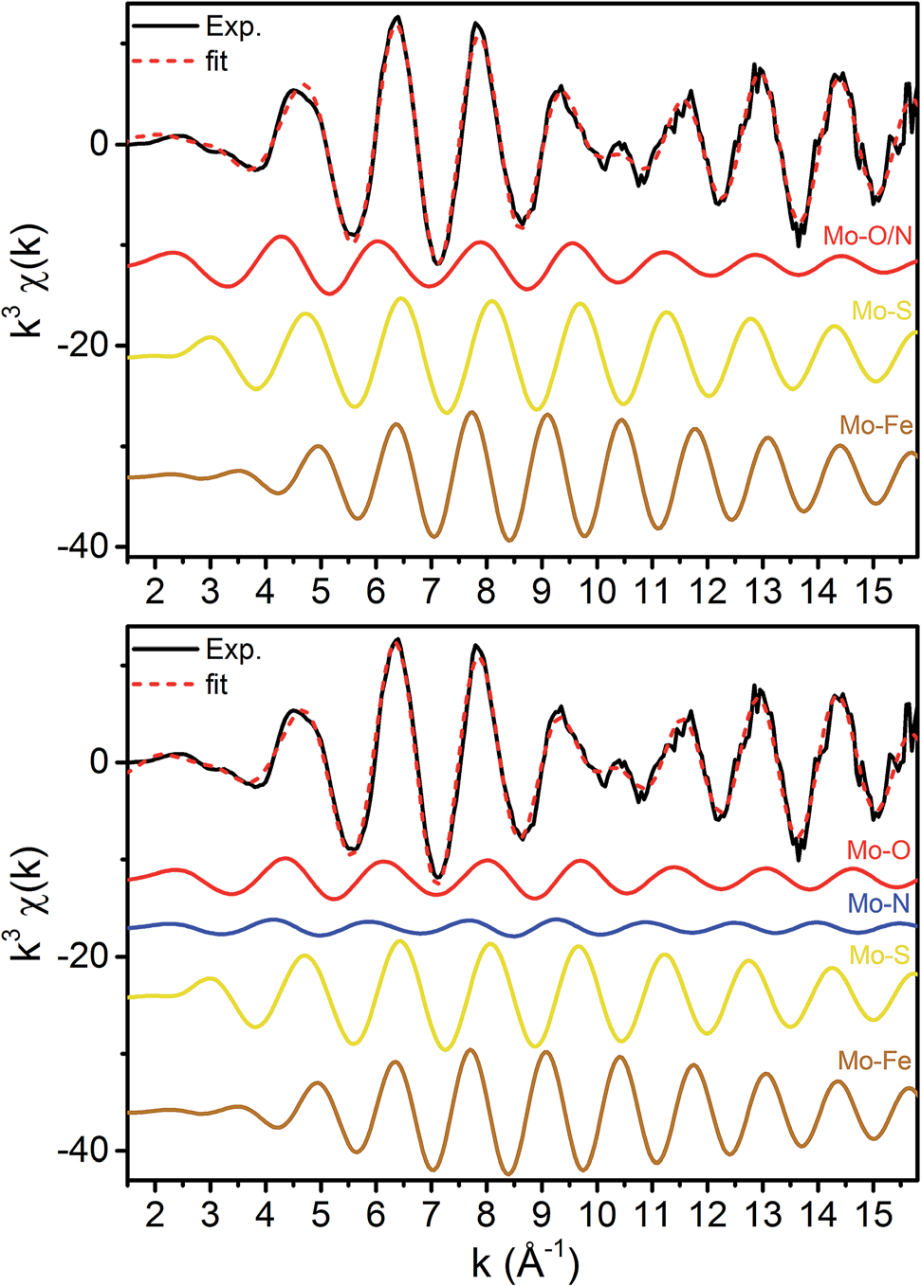
*k* ^3^-Weighted fits of the resting (E_0_) Mo K-edge EXAFS spectrum using (top) 3 paths and (bottom) 4 paths.

X-ray diffraction crystallography has clearly shown that the resting (E_0_) MoFe protein contains a single unique Mo, which is coordinated by homocitrate, histidine, and three inorganic sulfides from the FeMoco cluster.^29^,^51^ Additionally, there are three neighboring Fe atoms at ∼2.7 Å, ranging from 2.67 to 2.73 Å. Due to the relatively high symmetry of the Mo–S and Mo–Fe distances in the cluster, there are four possible short-range single scattering pathways from the Mo site, which include Mo–S, Mo–Fe, Mo–O, and Mo–N (Fig. 3). In a recent high-resolution crystal structure, the average Mo–O distance is approximately 2.18 Å (at 2.16 and 2.19 Å), while that of Mo–N is 2.33 Å.^29^ While N and O are generally indistinguishable by EXAFS due to their similar mass, the relatively large deviation between Mo–O and Mo–N bond distances determined by this structure (Δ*R* = 0.15 Å) in combination with the relatively high resolution of the present experiment (minimum Δ*R* = 0.108 Å for E_0_ and 0.112 Å for E_0_ + E_1_) warranted further investigation. From Fig. 4, it is clear that the Mo–O and Mo–N paths can be distinctly fit for the E_0_ state, meaning their fit paths are neither entirely destructive or constructive. However, the use of separate fitting paths results in only very minor statistical improvement (Table 1, 3- *vs.* 4-path fits for E_0_). Due to the small magnitude of the Mo–O/N path(s) contribution to the overall spectra, the fit parameters for these paths naturally have a larger degree of uncertainty than the heavier S and Fe scatterers.

**Fig. 5:**
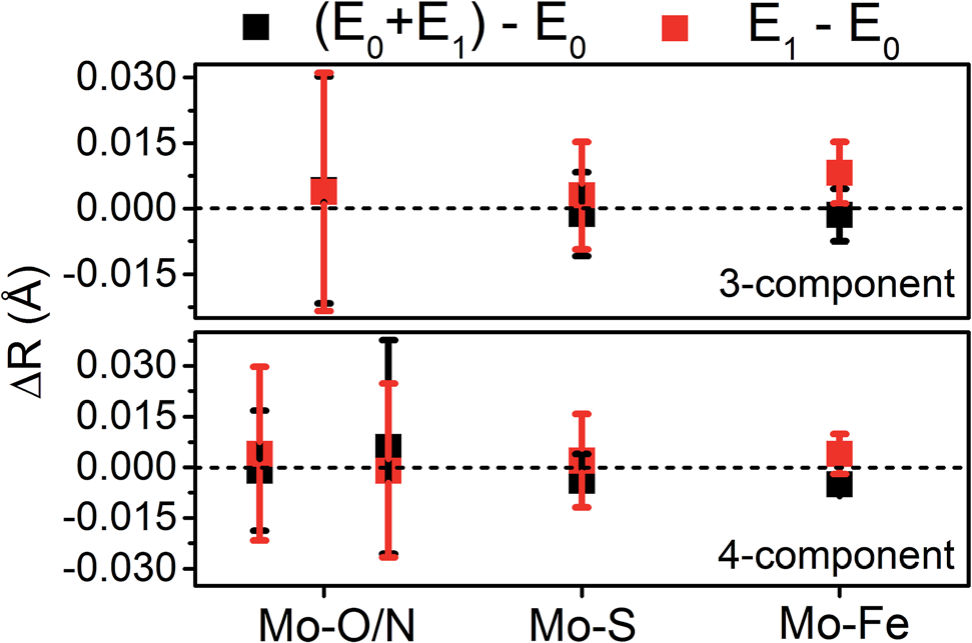
Comparison of variation in determined bond distances from Mo K-edge EXAFS. Changes in distances are calculated by subtraction of the fit E_0_ distances from those of the turnover (E_0_ + E_1_) and E_1_ fits. Error bars are reported on a 95% confidence interval.

Moving to the reduced E_0_ + E_1_ and E_1_ spectra, we find that no significant changes are observed in the Mo–S distance in either fit model relative to the resting E_0_ (Fig. 5). A small contraction in the average Mo–Fe distance is found when using either model to fit E_0_ + E_1_, while a small expansion is found for E_1_. As any real change in distance should trend when moving from E_0_ + E_1_ to E_1_ (as in an increase for E_0_ + E_1_ should be even larger in E_1_), we conclude that no significant structural changes occur from the perspective of Mo when progressing from the E_0_ to E_1_ state of MoFe.

### Fe EXAFS

**Fig. 6:**
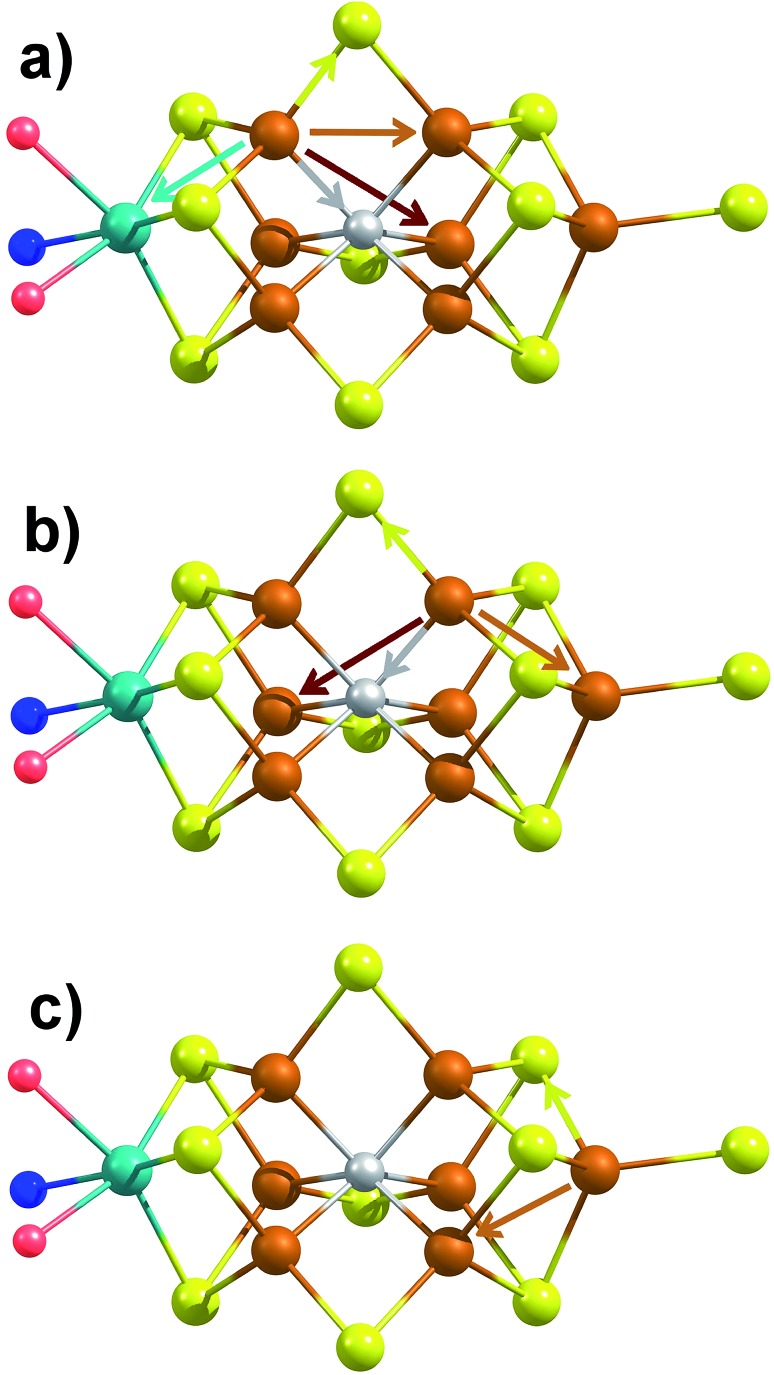
Depiction of the unique single-scattering paths for each of the three classes of Fe (a–c) in FeMoco. Generated using coordinates of XRD structure PDB ID 3U7Q.^29^ These include the Fe–Mo (cyan), Fe–C (gray), Fe–S (yellow), Fe–Fe(short) (rust), and Fe–Fe(long) (red) paths.

**Fig. 7:**
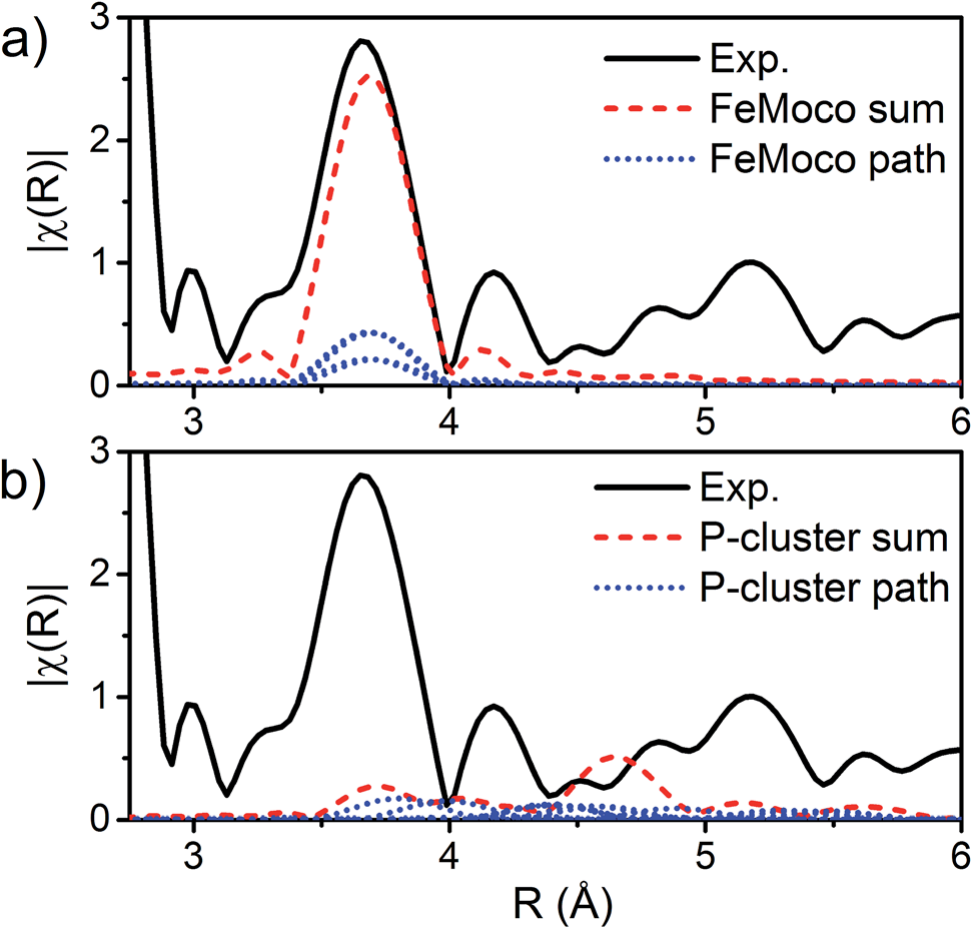
|FT| space spectrum of the Fe K-edge EXAFS of *E*_0_ (black, solid), scaled from 2.8–6 Å to compare the cumulative Fe–Fe(long) scattering paths for (a) FeMoco and (b) P-cluster, as determined by FEFF calculations of the ; 3U7Q XRD structure.^29^ The paths for each unique long Fe–Fe scatterer are depicted in blue (dotted), and the sum of these unique long Fe–Fe scatterers is shown in red (dashed). The high symmetry of the FeMoco cluster results in near-identical Fe–Fe(long) distances, which accumulate to provide a significant contribution to the spectrum, which can be modeled by a single Fe–Fe scattering path. Meanwhile, the long Fe–Fe distances in the P-cluster are highly disordered, making little contribution to the overall fit.

The major single-scattering paths in FeMoco from the perspective of Fe include Fe–S, Fe–Mo, Fe–Fe(short), and Fe–Fe(long). Additionally, it is known that a central carbide exists in the core of the FeMoco cluster, adding the possibility of a Fe–C path (Fig. 6a and b).^29^,^52^,^53^ Based on the XRD structure (PDB ID: ; 3U7Q), the FeMoco cluster is relatively symmetric and as a result the deviations in distance for a given path are quite small (for example, ∼0.04 Å in the long Fe–Fe path and ∼0.1 Å in the short Fe–Fe).^29^ However, the P-cluster presents a more complicated situation. While there are only Fe–S and Fe–Fe single scattering paths to consider for this cluster, its greater asymmetry results in a much wider distribution of distances. This becomes clear when examining distances determined in the high resolution crystal structure (PDB ID: ; 3U7Q),^29^ where the short Fe–Fe distances range from 2.50 to 2.92 Å (Fig. S2-2†). Similarly, the Fe–S distances vary from 2.25 to 2.47 Å, and the long Fe–Fe path from 3.79 to 5.48 Å. As a result of this distribution of distances, several considerations on how to appropriately treat these three paths in our model must be made, while still maintaining the minimal necessary number of variables. The Fe–S shell may still be modeled by a single path; however, the relatively large variation in these distances results in a greater degree of static disorder, and in turn a larger Debye–Waller factor. While the long Fe–Fe distances in the FeMoco cluster are nearly identical (Fig. 7a), fitting the highly disordered long Fe–Fe distances in the P-cluster (Fig. 7b) would necessitate separately treating numerous long Fe–Fe scattering paths. This approach would require the use of a large number of parameters, and would only make minor contributions to the long-range region. Therefore, the contribution of the long Fe–Fe scattering paths arising from the P-cluster are not considered in our model. The disorder of the short Fe–Fe distances in the P-cluster present an intermediate case, where they are disordered but still tightly clustered enough that they must be considered in the model. There is an existing precedence for (a) splitting the Fe–Fe(short) scattering path into two shells or (b) using a reduced number (*N*) of short Fe–Fe scatterers.^14^,^54^,^55^ The use of a reduced number *N* in the short Fe–Fe path operates under the assumption that the individual short Fe–Fe scattering paths in the P-cluster effectively cancel one another, as evidenced by an almost absent short Fe–Fe shell in the FT-EXAFS of the P-cluster only variant ΔnifB.^54^

To cover both approaches, models using a single Fe–Fe(short) scattering path, split Fe–Fe(short) scattering paths, and a single Fe–Fe(short) scattering path with reduced *N* = 1.65 were generated (fit parameters and figures are provided in the ESI†). In short, both the use of a split Fe–Fe(short) scattering path and single Fe–Fe(short) path with reduced *N* can be used to reasonably fit the data, while use of full *N* with only one Fe–Fe(short) scatterer results in a poor fit. However, use of a split Fe–Fe(short) shell demonstrates that the two Fe–Fe paths are not entirely destructive (Fig. S3-1†), and produce a nominal statistical improvement of the fit (Tables S5-3 and S5-6†). Therefore, the model used to fit the data presented here utilizes Fe–S, Fe–Mo, Fe–C, Fe–Fe(long), and split Fe–Fe(short) single-scattering paths. While previous EXAFS studies of the FeMoco cluster have found statistical improvement by the inclusion of a light Fe–X scatterer,^53^ our fits show that inclusion of the Fe–C path has little to no impact on either the statistics of the fit or the parameters determined for the other scattering paths. This discrepancy may in part be due to the presence of the P-cluster in the current samples, which significantly reduces the contribution of the Fe–C path to the overall EXAFS. The feasibility of objectively fitting the Fe–C scattering path is further discussed in Section S3 of the ESI.† Despite the lack of significant statistical improvement achieved by inclusion of the Fe–C scatterer, we have opted to still include this path in our presented 6-path model in acknowledgement of its presence.

**Fig. 8:**
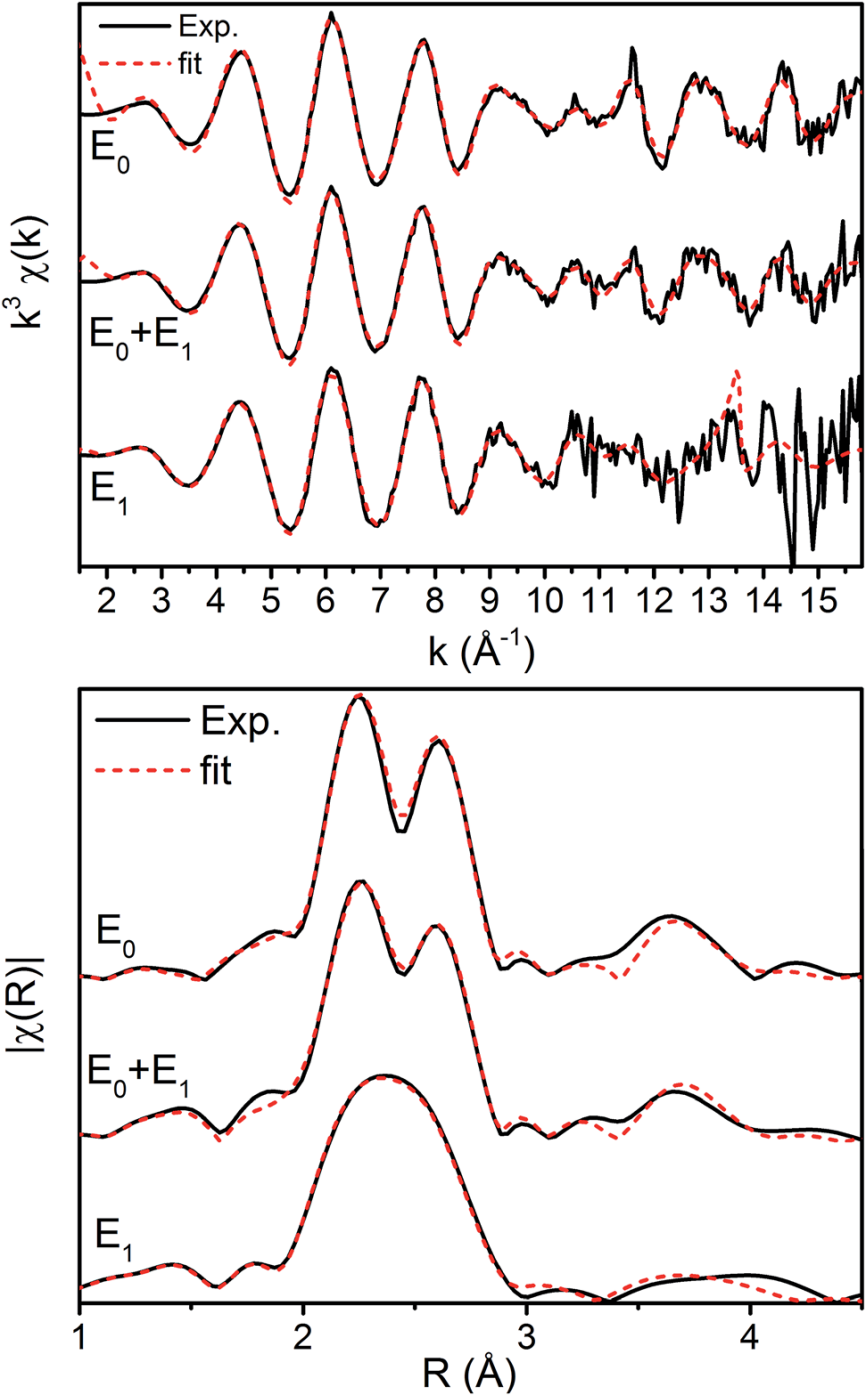
Fe K-edge EXAFS spectra of resting (E_0_), turnover (E_0_ + E_1_), and E_1_ state in both (top) *k*-space and (bottom) |FT| space. The *k*-space spectra are *k*^3^-weighted phase-shifted and the |FT| are phase corrected for the Fe–S scattering path. Solid black lines denote experimental spectra while red dashed lines indicate best fits using the 6 scattering path model.

The *k*^3^-weighted Fe EXAFS spectra of resting (E_0_), turnover (E_0_ + E_1_), and E_1_ MoFe are provided in Fig. 8 along with the corresponding FTs and best fits using the 6-component model. The corresponding parameters for these fits are provided in Table 2.

**Table 2:** Fe K-edge EXAFS fitting parameters of the 6 component model as applied to the resting (E_0_), turnover (E_0_ + E_1_), and E_1_ spectra. Statistical errors are provided for *σ*^2^ and *R* as determined from the fitting procedure

Fe FEFF fits, *E*_0_ = 7118.03 eV							
Sample	Path = 6	*N* ^*a*^	*S*_0_^2^	*σ* ^2^ (10^–3^ Å^2^)	*R* (Å)	*k* (Å^–1^)	*R*-Factor
Resting	C	0.4	0.9	2.00 (0.82)	1.911 (0.034)	2–15.8 min. Δ*R* 0.114	0.0142
	Mo	0.2		2.00 (0.62)	2.683 (0.013)		
	S	3.6		5.59 (0.44)	2.267 (0.002)		
	Fe-short	2.53		5.72 (1.30)	2.622 (0.003)		
	Fe-short	0.93			2.854 (0.012)		
	Fe-long	0.8		1.99 (0.09)	3.691 (0.010)		
Turnover (E_0_ + E_1_)	C	0.4	0.9	2.00 (2.81)	1.928 (0.024)	2–15.8 min. Δ*R* 0.114	0.0093
	Mo	0.2		2.00 (2.27)	2.679 (0.010)		
	S	3.6		6.10 (0.39)	2.273 (0.001)		
	Fe-short	2.53		6.45 (1.11)	2.620 (0.003)		
	Fe-short	0.93			2.858 (0.010)		
	Fe-long	0.8		1.99 (0.85)	3.704 (0.007)		
Turnover (E_1_)	C	0.4	0.9	2.00 (0.35)	1.992 (0.029)	2–13 min. Δ*R* 0.143	0.0079
	Mo	0.2		5.00 (0.00)	2.664 (0.001)		
	S	3.6		7.12 (0.00)	2.283 (0.003)		
	Fe-short	2.53		7.60 (0.91)	2.603 (0.003)		
	Fe-short	0.93			2.856 (0.010)		
	Fe-long	0.8		4.66 (2.10)	3.713 (0.014)		

^*a*^Please see Section S2 of the ESI for details on the determination of the path degeneracy *N*.

**Fig. 9:**
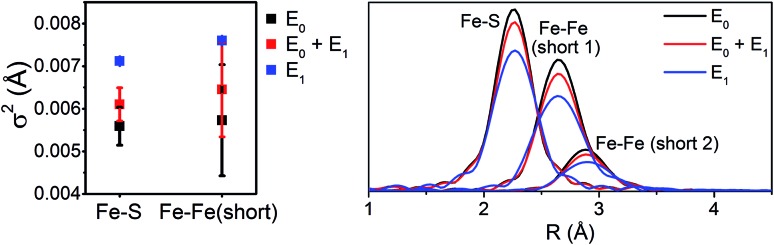
Comparison of *σ*^2^ (left) and |FT| (right) of the Fe–S, Fe–Fe(short 1), and Fe–Fe(short 2) paths for the 6-component model fits of resting (E_0_), turnover (E_0_ + E_1_), and E_1_ MoFe.

Interestingly, the splitting between the Fe–S and Fe–Fe(short) shells decreases moving from E_0_ to E_0_ + E_1_, and is completely lost in the simulated pure E_1_ spectrum. Fitting these data, it is apparent from the Debye–Waller factors of the Fe–S and Fe–Fe(short) shells that the degree of disorder of the metal clusters of MoFe increase when under turnover (Table 2 and Fig. 9). As all spectra were measured at the same temperature, it is sensible that this effect likely arises from static disorder. This is expected, considering the E_0_ + E_1_ spectrum represents a 50/50 mixture of the E_0_ and E_1_ states.

**Fig. 10:**
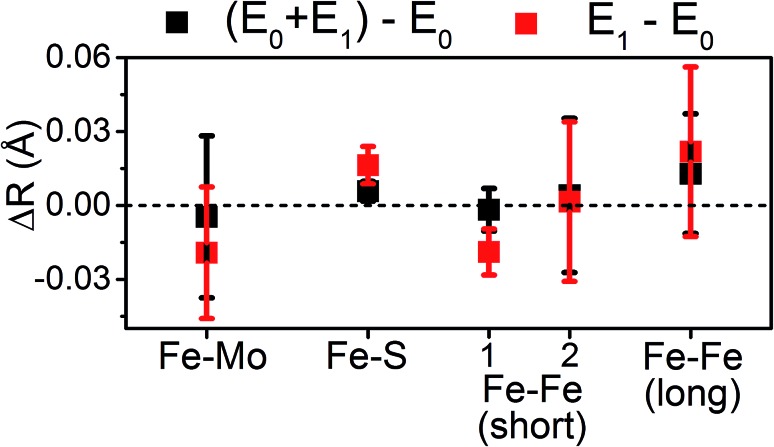
Comparison of variation in determined bond distances from the resting E_0_ state in the 6-component model applied to the Fe EXAFS. Distances are calculated by subtraction of the fit E_0_ distances from those of the E_0_ + E_1_ and E_1_ fits. The differences in the Fe–Fe(short) distances are split such that Fe–Fe(short 1) is to the left, and Fe–Fe(short 2) to the right. Error bars are reported on a 95% confidence interval.

The similarity of the Fe–Mo with Fe–Fe(short) distances combined with the relatively small contribution of the Fe–Mo scattering path to the overall fit results in considerable correlation between the fit Fe–Mo and Fe–Fe(short) parameters when determined from Fe EXAFS. As a result, there is an intrinsically large degree of error in the Fe EXAFS determined Fe–Mo distances. Regardless of the applied model, no significant changes in the Fe–Mo are observed, consistent with results obtained from the Mo EXAFS. Generally, a small but statistically significant increase in the Fe–S distances is found, while a small decrease in the Fe–Fe(short 1) distance is observed in fitting the E_1_ spectrum (Fig. 10). These changes hold true for all models investigated in the present study (Fig. S5-9†). It is worthwhile to note that while the Fe–S path represents an average of both FeMoco and P-cluster, the Fe–Fe(long) path is predominately representative of the FeMoco cluster. Additionally, by splitting the short Fe–Fe path, the first Fe–Fe(short 1) path represents a combination of the FeMoco and P-cluster Fe–Fe(short) scatterers, while Fe–Fe(short 2) should only be representative of the P-cluster. It is therefore not surprising to see that Fe–Fe(short 1) contracts while no effective changes are observed for Fe–Fe(short 2).

Thus far we have rigorously reexamined the Mo and Fe EXAFS of the E_0_ and E_1_ states of MoFe using a variety of possible models. The presented results have demonstrated that no effective changes are found in the first coordination sphere of the Mo of the FeMoco cluster, while all fit models show small but consistent variations in the Fe distances. With these results in hand, we turn to theoretical methods, specifically QM/MM, for a more detailed investigation of the specific nature of the E_1_ state.

### QM/MM calculations

We performed a series of quantum mechanics/molecular mechanics (QM/MM) calculations to investigate the possible electronic states accessible by single e^–^ reduction of FeMoco and the nature and position of the transferred H^+^ in the FeMoco cluster (*i.e.* iron-hydride formation *vs.* sulfur protonation). A previous QM/MM study of resting state MoFe protein by one of us has demonstrated that very good agreement between the computed FeMoco structure and the high-resolution crystal structure can be obtained when the protein environment is included *via* QM/MM and the TPSSh hybrid functional is used.^24^ Here, we employ an almost identical computational approach to study the changes which occur going from the E_0_ to E_1_ states. To reduce systematic errors present in the calculations (such as any over- or underestimation of the covalency of specific chemical bonds, or errors resulting from the simplified spin-coupling treatment employed), we will focus on the changes which occur in E_1_ relative to the resting E_0_ state. Additionally, in order to compare the changes in calculated bond distances Δ*R* (defined by *R*(E_1_) – *R*(E_0_) for a given path), with those determined by EXAFS, we have averaged the Mo and Fe bond distances to the same level of resolution as observed in the Mo EXAFS 4-path model, and the Fe EXAFS 6-path model.

In approaching the question of the identity of E_1_, three major considerations must be made, namely (a) the protonation state of the Mo-binding homocitrate in E_0_ and E_1_, (b) the location of protonation on the FeMoco cluster (as well as whether it even occurs), and (c) the possible electronic states accessible upon reduction, directly related to the site of reduction as discussed later. Additionally, as it is unknown whether the N_δ_ or N_ε_ position of His195 is protonated in E_1_, we have considered both possibilities.

The protonation state of homocitrate has been previously discussed for the E_0_ state, where comparison of computed distances (primarily the distance between the oxygens of the hydroxyl and carboxylate groups in homocitrate) and quantum crystallographic refinement have indicated the Mo-bound hydroxyl group is protonated.^24^,^25^ We revisit this protonation assignment here in the context of E_1_, and discuss it in greater detail in Section 6.2 of the ESI.† Briefly, all models which leave the homocitrate unprotonated (in either the resting E_0_ or reduced E_1_ states) result in the highest occupied molecular orbitals (HOMOs) having positive energies, which is unphysical. Additionally, models involving deprotonated homocitrate result in the contraction of the averaged Mo–O bond by 0.05 Å and lengthening of both Mo–S and Mo–Fe by ∼0.02 Å when moving from E_0_ to E_1_, disagreeing with the EXAFS results. Meanwhile, all models which retain a protonated homocitrate in both E_0_ and E_1_ states show a mild expansion of the average Mo–O/N distance by ∼0.02 Å, a negligible expansion of the Mo–S distances, and a small contraction of the Mo–Fe distance by ∼–0.02 Å. The combination of the strong Mo–O contraction, which is well out of the standard error of the experiment, combined with the non-physical energies of the HOMOs indicate that the Mo-bound hydroxyl group of homocitrate is likely protonated in both the E_0_ and E_1_ states, and will be considered as such for the remainder of the results.

We have found that addition of an electron to the FeMoco in the absence of a proton results in mild modulation of all bond lengths, which are in reasonable agreement with those observed in the EXAFS (Fig. S6.4-3 through S6.4-8†). However, similar to the case of the unprotonated homocitrate, the energies of the HOMOs become positive (see Tables S6.4-3 through S6.4-6†), suggesting that protonation of FeMoco may be required to obtain a physically relevant E_1_ state. It has already been established that the belt sulfides of the cluster (S2B, S3A, and S5A) are the most basic according to another detailed QM/MM study,^18^ and we have therefore focused the present study on the protonation of these sulfurs. Additionally, we have investigated the possibility of terminal hydride formation at the sterically unhindered Fe6, as well as the protonation of S1A and S3B sites. While a bridging hydride model was considered, it was found to be unstable and consistently converted to terminal coordination upon optimization; previous computational studies of several bridging hydride models of E_1_ were also found to be highly energetically unfavorable.^18^ The direction of protonation in each of these models is discussed in Section S6.3.2 of the ESI.† Finally, we investigated the possibility of a carbide protonation in E_1_, as this has been suggested to be thermodynamically favorable according to calculations by Rao, *et al.*^56^ For each of these protonation states, we have further considered three unique electronic states of the cofactor (referred to as broken-symmetry (BS) solutions BS-235, BS-346 and BS-247) which correspond to different locations of the Fe up/down local spins and delocalized pairs in the cluster. In this nomenclature, the first two numbers indicate the two Fe which form a spin-down mixed-valent delocalized pair in the [Fe_4_S_3_C] sub-cubane, and the third denotes the Fe which becomes “spin-down” in the [MoFe_3_S_3_C] sub-cubane. This third Fe is spin-localized, is either ferric (in E_0_) or ferrous (in E_1_). Section S5.1 of the ESI† provides a more detailed description of this scheme. Although all three BS solutions share the same favorable antiferromagnetic spin orientation (known as BS7 in the literature),^50^ it is necessary to consider all three since the effective *C*_3v_ symmetry of the cluster is broken by the secondary environment of the protein. In the context of E_1_, these three solutions effectively rotate which iron in the cluster is reduced (Fe4 or Fe5 in BS-235, Fe2 or Fe6 in BS-346, and Fe3 or Fe7 in BS-247; see Fig. 1). While it is possible that the added electron could be on either of two Fe atoms for a given BS solution, we have found that reduction is always localized to one of the Fe atoms of the [MoFe_3_S_3_C] sub-cubane (Fe5 through Fe7). Other electronic states that would hypothetically result in reduction of the [Fe_4_S_3_C] cubane were considered but found to be energetically unfavorable (>15 kcal mol^–1^), in agreement with a previous study by Cao *et al.*^18^

The presented broken-symmetry solutions have a final spin-state of *M*_S_ = 2, which was found to be generally energetically more favorable than *M*_S_ = 1 (with the Fe6-hydride state being the only exception, where the *M*_S_ = 1 and *M*_S_ = 2 energies are comparable). This is the logical spin state when a spin-down high-spin Fe(iii) (local spin 5/2) is reduced to spin-down Fe(ii) (local spin 2), changing the total spin-state of the cluster from *M*_S_ = 3/2 (E_0_) to *M*_S_ = 2 (E_1_) within a highly simplified spin-coupling model. This is in good agreement with the experimental spin states of [MoFe_3_S_4_]^3+^ (*S* = 3/2) and [MoFe_3_S_4_]^2+^ (*S* = 2) synthetic cubanes.^57^,^58^ Further details about the electronic states can be found in the ESI, Section S6.1.†

To compare the structures of the E_1_ models featuring different protonation/electronic states to the new EXAFS data in an unbiased fashion, it is helpful to first introduce and discuss a simple nonbiased metric. In this regard, the root-mean-square deviation (RMSD) of the *R*(E_1_) – *R*(E_0_) structural differences relative to the experimental EXAFS data is quite useful; however, as the experimental standard deviation for different scattering paths can vary considerably (see Fig. 5 and 10), a Gaussian-based weighting scheme has been employed that takes the experimental deviation into account in the RMSD metric. This approach is detailed in the statistical analysis section of the Experimental details.

**Fig. 11:**
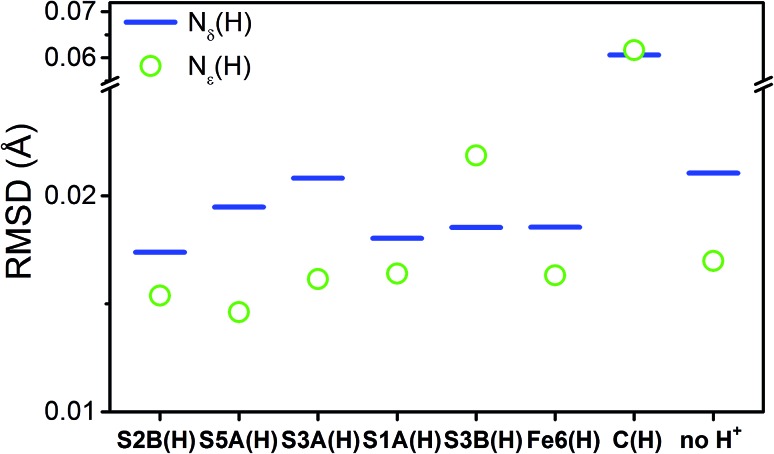
Weighted root-mean-square deviations for the calculated changes in distances (Δ*R*^calc^) of the computational models of the E_1_ state averaged across the BS-235, BS-346, and BS-247 solutions. Each individual contributing path has been weighted based on the experimental standard deviation, as described in the statistical analysis section of the Experimental details. Experimental changes in distances (Δ*R*^exp^) used in these calculations were acquired from E_1_–E_0_.

Fig. 11 shows the weighted RMSDs for the different computational models, where the results for each protonation state have been averaged over the BS electronic states (for the RMSDs of individual BS solutions, see Fig. S6.4-10 and 6.4-11 in the ESI†). The results most clearly reveal that the model involving a protonated carbide strongly deviates from the EXAFS results, featuring an RMSD >0.06 Å. The most favorable models also vary considerably depending on whether the N_ε_ or N_δ_ positions of His195 are protonated. In the case of His195-N_δ_(H), the S2B(H) position appears to most favorably agree, followed by the S1A(H); both the S3A(H) and “no H^+^” models appear poor. Of the models involving His195-N_ε_(H), the S5A(H) and S2B(H) are in most favorable agreement, while S3B(H) fits relatively poorly. It is worthwhile to note that for most models the N_ε_ protonated state of His195 generally gives consistently lower RMSDs than the N_δ_ protonated state.

**Fig. 12:**
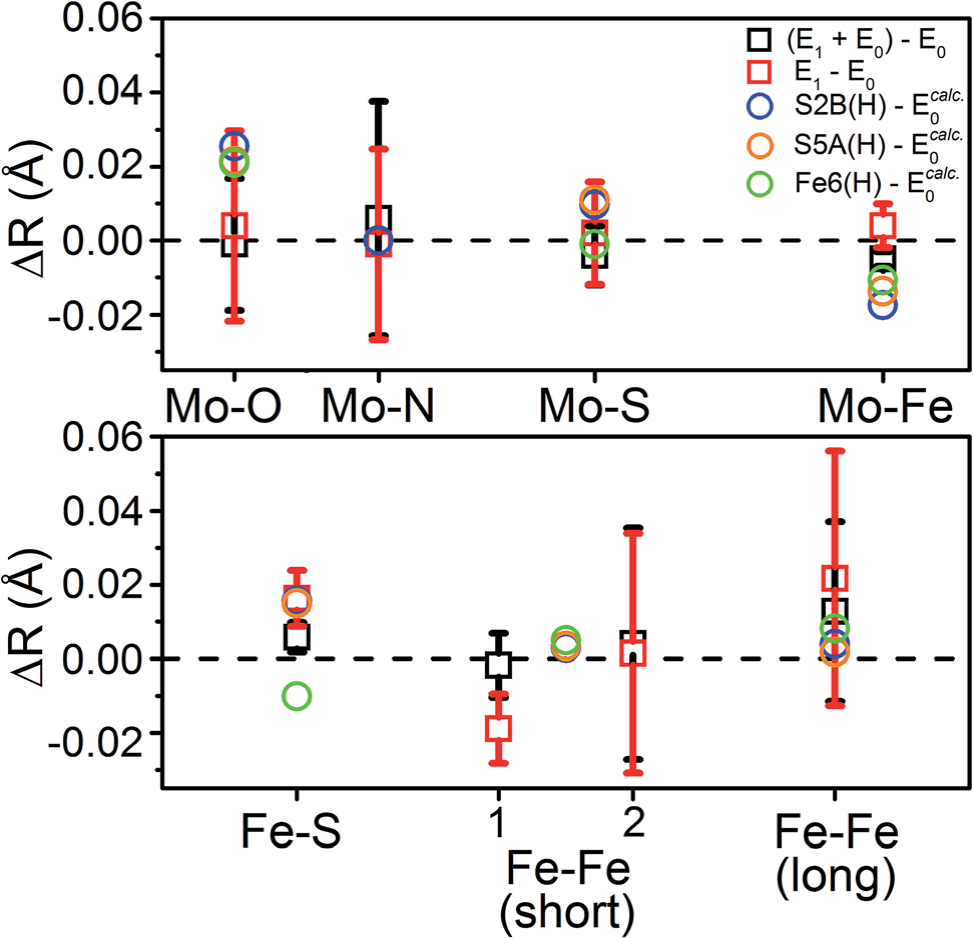
Comparison of the change in calculated distances upon reduction from E_0_ → E_1_ for the S2B(H), S5A(H), and Fe6(H) models in the BS-235 electronic state, with His195 treated as N_ε_(H). Experimental Fe–Fe(short) distances correspond with the (left) Fe–Fe(short 1), *N* = 2.53 and (right) Fe–Fe(short 2), *N* = 0.93 shells described in Table 2. E_0_ calculated distances from the BS-235 solution are used in the calculated differences, as previous comparisons have shown this solution to best reproduce those determined in the ; 3U7Q crystal structure.^24^ A full comparison of all calculated changes in relevant bond distances for all 6 protonated models (in both the N_ε_(H) and N_δ_(H) singly-protonated states of His195), in all three considered BS-solutions, relative to all three BS solutions of the E_0_ state are provided in Fig. S5.6-3 through S5.6-8.† Error bars for the experimentally determined distances are reported on a 95% confidence interval.

To distinguish these models further, we turn to how well each reproduces the experimentally observed changes in the individual scattering paths. As an example, the changes in the average calculated Mo and Fe distances is provided in Fig. 12 for the S2B(H) and Fe6(H) models. Section 5.6 of the ESI† contains complete comparisons of the calculated changes in bond distances for all E_1_ models, with protonation of either the N_ε_ or N_δ_ positions of His195, in all three considered BS solutions, relative to the complimenting three BS solutions of the E_0_ state. All three BS solutions for all models display an increase in the average Mo–O bond length. This increase in Mo–O bond length is relatively mild in most cases, ranging from 0.01–0.03 Å, although the “no H^+^” model consistently results in expansions of ∼0.04–0.05 Å. Although the EXAFS cannot resolve the individual Mo–O paths, we note that this calculated increase is almost completely attributable to the Mo–O(2) bond (Fig. 1), which corresponds to the hydroxy group of homocitrate. No effective increase in the Mo–N_His442_ distance is found in any of the calculations, for any model or BS solution. All models display a mild increase in the average Mo–S distance; most are within the range of experimental error, although the S3B(H) model presents a significantly greater expansion than the others (∼0.03 Å). Lastly, all belt sulfide models (as well as S1A(H)) show small decreases of 0.005–0.03 Å in the average Mo–Fe distance; this decrease is particularly exacerbated in the case of S3A(H) (Fig. S6.4-5†).

Comparing the average calculated Fe distances with experiment, we find that the expansion of the Fe–S path is reasonably reproduced by the three belt-sulfide protonated models, as well as S1A(H) and “no H^+^”. Meanwhile, the Fe6-hydride model displays a decrease in average Fe–S distance, counter to our EXAFS results. No model is capable of reproducing the contraction found in the Fe–Fe(short 1) distance, although the three belt-sulfide protonated models show no effective change while all others show an expansion (Fig. S6.4-6 and S6.4-7†). The C(H) model is particularly extreme, with an expansion of ∼0.08 Å. All models also predict virtually no change in the average Fe–Fe(long) distance with the exception of C(H), which shows an expansion of up to ∼0.14 Å (Fig. S6.4-8†).

From our comparisons with the present EXAFS results, we conclude that the S2B(H), S5A(H), and S1A(H) of models of E_1_ appear the most reasonable. While the weighted RMSD metric suggested the Fe6(H) hydride model as also reasonable, the considerable overestimation of the change in Mo–O and the wrong trend in the change of average Fe–S distance make this model less likely.

**Fig. 13:**
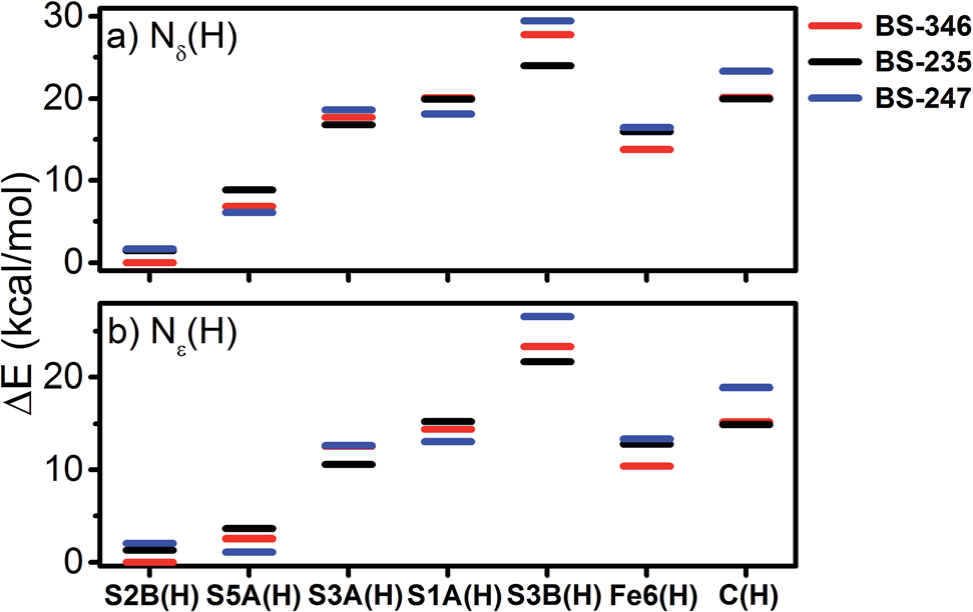
Relative energies of the calculated energies of the investigated protonated models of E_1_ with both (a) N_δ_(H) and (b) N_ε_(H) protonation states of His195. Displayed energies are QM/MM total energies.

With this experimental parameterization in hand, we can compare the relative energies of these different states (Fig. 13). We find that the S2B(H) model is most favorable when N_δ_(H) is used at His195, with the S5A(H) model appearing ∼7 kcal mol^–1^ higher in energy. However, the energies of the S5A(H) and S2B(H) models become comparable when the N_ε_ position of His195 is protonated. Meanwhile, the S3A(H), S1A(H), Fe6(H), S3B(H), and C(H) models all appear considerably unfavorable in either of the His195 protonation states, with energies >10 kcal mol^–1^ higher than those of the lowest energy solutions.

## Discussion

The present results show that very little change occurs in the metal clusters of MoFe upon formation of E_1_, counter to the results of a previous EXAFS study.^14^ Particularly, we find that the large contractions in Mo–O/N, Mo–Fe, and Fe–Fe(short 1) previously reported from Mo and Fe K-edge EXAFS do not occur in the present system (Table 3). One plausible explanation for the large discrepancies between the present study and the previously reported^14^ Mo and Fe K-edge EXAFS lies in the fitting procedure used to interpret the data. In particular, the models employed in the previous EXAFS study utilized significantly path-dependent values of Δ*E*_0_, the parameter which is used to align the energy grids of the experimental spectrum and the model.^14^ Fundamentally, *E*_0_ (not to be confused with the resting E_0_ state of N_2_ase) is used to describe the kinetic energy necessary for a photoelectron to escape an absorber. Therefore, it is characteristic of the absorbing atom and *independent of the scattering path*. Small variations in Δ*E*_0_, on the order of up to a couple eV, are generally acceptable when describing the paths of two unique absorbers of the same type in the same sample. For example, EXAFS fitting of NiFe or FeFe hydrogenases may require two unique Δ*E*_0_, one for the Fe of the 4Fe–4S clusters, and a second for the Fe–Fe active site. In the previous report of the Mo and Fe EXAFS of MoFe, large variations in Δ*E*_0_ of up to 17 eV and 7 eV were employed for different paths involving Mo and Fe, respectively.^14^ As only a single species of Mo is present in these samples, there is no physical justification for using radically different values of Δ*E*_0_ for the various scattering pathways. These changes in Δ*E*_0_ effectively change the phase of the individual fit paths relative to one another, making the bond distances determined by said fit effectively arbitrary (please see Section S4 of the ESI† for further discussion). This is an unfortunately common mistake in EXAFS fitting;^59^–^64^ to quote Scott Calvin – “There are few mistakes more common in published EXAFS work, and more unambiguously wrong, than publishing fits where every path has a unique *E*_0_…”.^65^

**Table 3:** Comparison of the present EXAFS determined changes in path lengths (E_1_–E_0_) with those previously reported.^14^ “present” distances were determined from the 3-path Mo and 6-path Fe fits. All standard errors are rounded to the nearest third decimal

Path	Δ*R*(E_1_ – E_0_) (Å)	
	Present	Ref. 14
Mo–O/N	0.004 ± 0.014	–0.07
Mo–S	0.003 ± 0.006	0.00
Mo–Fe^*a*^	0.008 ± 0.004	–0.06
Fe–S	0.016 ± 0.004	0.02
Fe–Fe(short 1)	–0.019 ± 0.005	–0.04 to –0.06
Fe–Fe(short 2)	0.002 ± 0.016	–0.01 to –0.02
Fe–Fe(long)	0.022 ± 0.017	0.01 to 0.02

^*a*^Mo-Fe path distances shown were determined from Mo K-edge EXAFS.

**Fig. 14:**
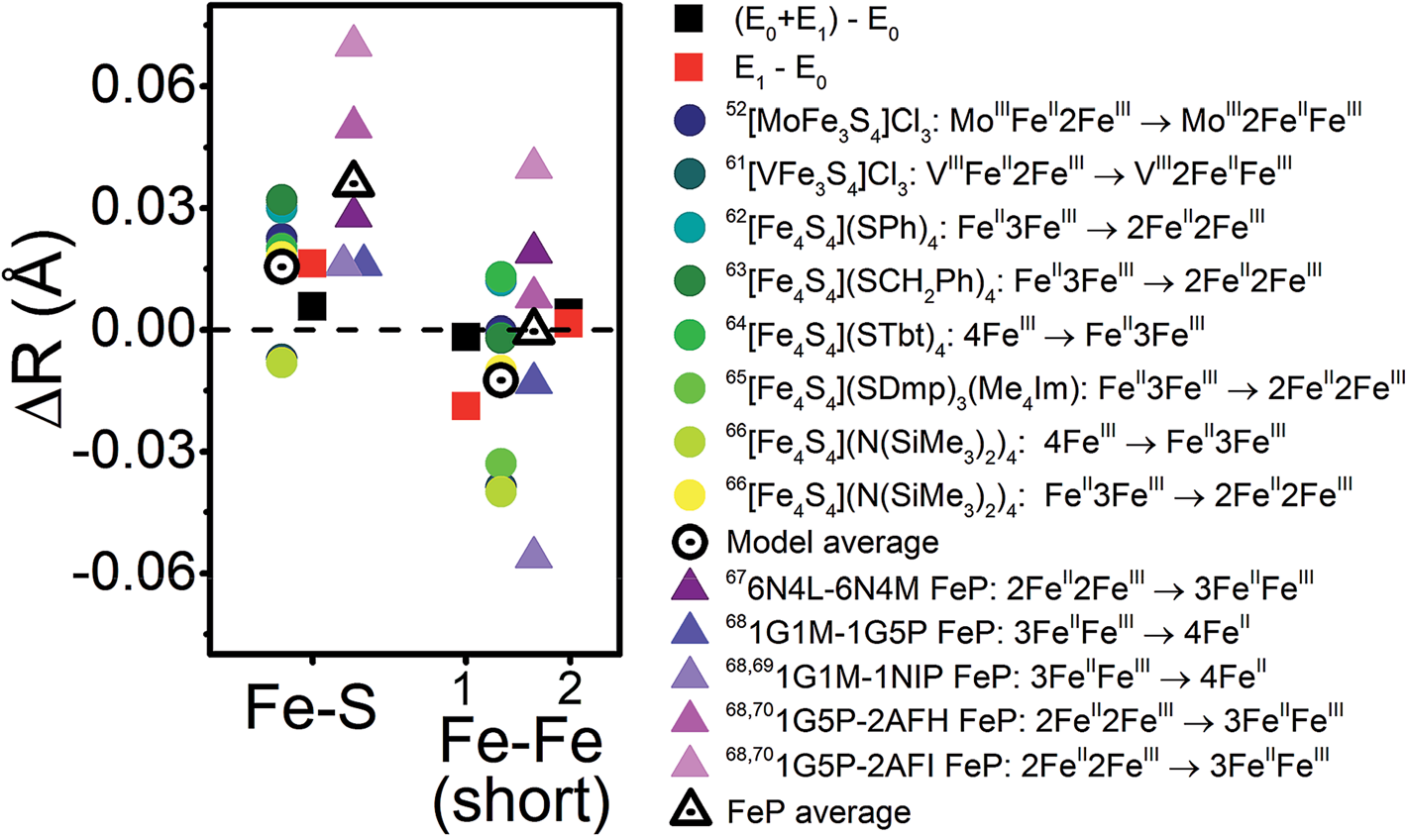
Comparison of the changes in observed core Fe–S and Fe–Fe distances upon reduction for the turnover (E_0_ + E_1_) and E_1_ states (black and red squares, respectively) with those observed in a series of cubane model complexes^57^,^66^–^71^ (circles) and in FeP^72^–^75^ (triangles) based on XRD. The differences in Fe–Fe(short) distances are split to represent Fe–Fe(short 1) to the outside-left, and Fe–Fe(short 2) to the outside-right.

With the experimental bond distances of E_0_ and E_1_ in hand, we can compare the observed differences between these two states with those of model complexes and other FeS cluster proteins. Fig. 14 provides the changes in average Fe–S and Fe–Fe distances between several oxidation states in a variety of model cubane clusters and in FeP, based on various literature reports. On average, the Fe–S distance increases by 0.015–0.03 Å in both the cubane model complexes and FeP. Meanwhile, a spread of changes are observed in the Fe–Fe(short) distances, ranging from expansion to contraction. The model cubane complexes show a small decrease on average, while those observed for the FeP are dependent on the redox couple (where a slight expansion is seen going from 2Fe^II^2Fe^III^ → 3Fe^II^Fe^III^, and a contraction going from 3Fe^II^Fe^III^ → 4Fe^II^). We can conclude from these comparisons that both the sign and magnitude of the observed changes are consistent with an Fe-centered reduction.

We can now use our revised understanding of the structural changes that occur upon reduction of MoFe from E_0_ to E_1_ as a metric to judge the various QM/MM models of E_1_. The structural changes which occur in several of the QM/MM models when moving from E_0_ → E_1_ provide reasonable agreement with the EXAFS data, while several others do not. In nearly all cases, the Mo–O bond length changes were found to be overestimated compared to the EXAFS data. This overestimation relates to the proton of the hydroxy group, which forms a strong hydrogen bond with the carboxylate-arm of the homocitrate (see Fig. S6.4-2†). The strength of this hydrogen bond is highly dependent on how well the hydrogen-bonding environment is described in the model, and hence can directly impact the calculated Mo–O distance. We have previously observed spontaneous proton transfer to the carboxylate group in some FeMoco models, revealing a sensitivity to the precise location of this proton; under experimental conditions this acidic proton may even be delocalized between these oxygen atoms, a feature that our computational models are currently unable to capture.

The energetic comparison in Fig. 13 reveals large differences between the investigated models, which are overall in agreement with the result of a previous computational study.^18^ The bridging sulfides are generally more basic than other sulfides, with S2B and S5A being much more favorable than S3A. The variation between the three belt-sulfide positions is likely related to the peptide backbone environment near S3A, as peptide NH groups show weak hydrogen bonds surrounding this sulfide which render protonation unfavorable. Calculations of these E_1_ models in the absence of the MM environment (using instead a polarizable continuum model) confirm the secondary coordination environment as the source of this disparity, where protonation becomes almost equally favorable for all belt-sulfide sites (see Fig. S6.3-1 of the ESI†). Additionally, the formation of Fe6(H), a terminal hydride, is not thermodynamically favorable at the redox level of E_1_.

Several studies have suggested that His195 functions as a competent proton donor to the FeMoco cluster;^76^–^78^ indeed, the orientation of His195 and 3.2 Å S2B-N_ε_ distance in the ; 3U7Q crystal structure of E_0_ suggests that His195 is likely protonated at N_ε_, forming a hydrogen bond to S2B.^29^,^79^ Proton transfer from N_ε_(H) of His195 to S2B could plausibly result in immediate reprotonation of His195. One possible proton pathway involving His195, Tyr281 and a water molecule has been previously discussed by Dance, where reprotonation of His195 occurs at the N_δ_ position.^19^,^76^ Although this is a plausible scenario, there is no direct experimental evidence for this inverse protonation state in any E_*n*_ state. If a regular protonation state of His195 is considered instead, the S2B and S5A become equally plausible protonation sites in E_1_, based on the relative energies (∼2 kcal mol^–1^, see Fig. 13), likely due to reduced basicity of the S2B site *via* the His195-S2B hydrogen bond. We note that a previous QM/MM study found a larger energy difference (7.6 kcal mol^–1^) between the S2B(H) and S5A(H) E_1_ models; the reasons for this difference are not clear but may be related to the use of different functionals (that has been the subject of discussion in the literature^80^) or slightly different QM/MM models.

As previously mentioned, the electronic state considered appears to determine the specific site of reduction on FeMoco (Fe5, Fe6 or Fe7). One might imagine the electron and proton ending up at the same or neighboring sites (*e.g.* a redox event at Fe6 resulting in protonation of Fe6-bound S2B, a E_0_(BS-346) → E_1_(346)-S2B(H) scenario). Comparing the QM/MM determined energies, we can see that the BS solution which places the additional electron either directly on or neighboring the site of protonation becomes most favorable in all cases. However, this effect is only a few kcal mol^–1^ at best. This implies that while the basicity/hydricity of the protonation sites investigated are, to a certain degree, sensitive to the location of the additional electron, the surrounding secondary environment of the cluster plays a dominant role in determining the most favorable protonation site.

To conclude, E_1_ models featuring a protonated bridging sulfide fit best with the EXAFS data. Based on the relative energies of these various models, those involving a protonated sulfide at either the S2B or S5A positions in tandem with an Fe-centered reduction at the [MoFe_3_S_3_C] sub-cubane are the most likely candidates to describe the E_1_ state. These results are in good agreement with numerous experimental studies of ligand-bound states of both Mo and V nitrogenase in which substitution of the belt sulfides occurs.^81^–^85^ Further distinguishing between S2B and S5A as protonation site in E_1_ likely requires experimentally establishing whether His195 can serve as a direct proton donor in the E_0_ → E_1_ process. The similar basicity of these sulfide sites also suggest that both may play a role in the formation of other reduced states of FeMoco, *e.g.* E_4_, where potentially 4 protons have been added to the cofactor.

## Conclusions

Determining how nitrogenases are capable of accumulating 3–4 reducing equivalents, while maintaining effectively the same reduction potential, is critical in understanding how these enzymes bind and activate N_2_. The E_1_ state represents the first critical step in this process. The present study has used Mo and Fe K-edge EXAFS to characterize the structural changes which occur upon reduction of E_0_ to E_1_. While a previous report claimed large contractions occur in Mo–O, Mo–Fe, and short Fe–Fe distances,^14^ we have found that only minor modulation of these distances occurs. By comparing our observations with both known model complexes and FeP, we have found that formation of the E_1_ state is consistent with an Fe-centered reduction, in agreement with our previous observations.^15^ Furthermore, the combination of the present EXAFS results with QM/MM calculations supports that protonation of a belt sulfide likely occurs in E_1_, most favorably at the S2B or S5A positions.

## Conflicts of interest

There are no conflicts to declare.

## Supplementary Material

Supplementary information

Supplementary information
