# Supplementary material for: Resolving the structure of the E1 state of Mo nitrogenase through Mo and Fe K-edge EXAFS and QM/MM calculations
Source: Chem Sci. 2019 Sep 4;10(42):9807–21. doi: 10.1039/c9sc02187f (PMC6984330; doi:10.1039/c9sc02187f)
Supplement: Supplementary file 1 [file SC-010-C9SC02187F-s001.pdf]

Electronic Supplementary Material (ESI) for Chemical Science.
This journal is © The Royal Society of Chemistry 2019

Supporting Information: Resolving the structure of the E_1 state of Mo Nitrogenase using Mo and Fe K-edge EXAFS and QM/MM calculations

Casey Van Stappen, Albert Thor Thorhallsson, Laure Decamps, Ragnar Bjornsson*, Serena DeBeer*

[†]Max-Planck Institute for Chemical Energy Conversion, Stiftstrasse 34-36, 45470 Mülheim an der Ruhr, NRW, Germany.

email: serena.debeer@cec.mpg.de, ragnar.bjornsson@cec.mpg.de

Table of Contents

S1. EPR of the resting and low-flux turnover states of MoFe 3
Figure S1-1. 3

S2. Acquiring and adjusting degeneracy „*N*“ 4
Figure S2-1. 5
Figure S2-2. 6

S3. The feasibility of fitting the Fe-C scatterer 8
Figure S3-1. 8
Figure S3-2. 9

S4. Why is E_0 important? 10
Figure S4-1. 11
Table S4-1. 11

S5. Supplementary XAS Figures & Tables 12
Figure S5-1. 12
Figure S5-2. 13
Figure S5-3. 14
Figure S5-4. 14
Figure S5-5. 15
Figure S5-6. 15
Figure S5-7. 16
Figure S5-8. 16
Figure S5-9. 17
Table S5-1. 18
Table S5-2. 18
Table S5-3. 19
Table S5-4. 19
Table S5-5. 20
Table S5-6. 20

S6. Computational Details 21
S6.1. Labelling scheme of the broken-symmetry states & Selection of broken-symmetry states for investigation 21
Figure S6.1-1. 21
Figure S6.1-2. 23
Table S6.1-1 23
Table S6.1-2 24
Table S6.1-3 24

S6.2. Determination of the homocitrate protonation state 25
Figure S6.2-1. 25
Figure S6.2-2. 26
Figure S6.2-3. 27
Table S6.2-1. 27
Table S6.2-2. 27
Table S6.2-3. 28
Table S6.2-4. 28
Table S6.2-5. 28
S6.3. Protonated models of E_1 29
S6.3.1 Protonated cluster models of E_1 in a polarizable continuum 29
Figure S6.3-1. 29
S6.3.2 Direction of protonation in QM/MM models of E_1 29
S6.4. Figures & Tables 30
Figure S6.4-1. 30
Figure S6.4-2. 31
Figure S6.4-3. 31
Figure S6.4-4. 32
Figure S6.4-5. 32
Figure S6.4-6. 33
Figure S6.4-7. 33
Figure S6.4-8. 34
Figure S6.4-9. 34
Figure S6.4-10. 35
Figure S6.4-11. 35
Table S6.4-1. 35
Table S6.4-2. 36
Table S6.4-3. 36
Table S6.4-4. 36
Table S6.4-5. 37
Table S6.4-6. 37
References 38

S1. EPR of the resting and low-flux turnover states of MoFe

EPR measurements were performed to establish the relative degree of reduction of the $S = {}^{3}/_{2}$ signal of the E_0 resting state of MoFe N_2ase in turnover samples. These measurements were performed on the exact samples which were used for further XAS measurements. All continuous wave (CW) X-band EPR measurements were recorded using a Bruker E500 ELEXSYS spectrometer equipped with a Bruker dual-mode cavity (ER4116DM) and an Oxford Instruments helium flow cryostat (ESR 900). All measurements were performed at 10 K. A high-sensitivity Bruker Super-X (ER-049X) bridge with integrated microwave frequency counter was employed as the microwave unit. A magnetic field controller (ER032T) was externally calibrated with a Bruker NMR field probe (ER035M). X-band measurements utilized a 2 mW microwave power and 0.746 mT/100 kHz modulation. Spectra were analyzed in both the local software package esim_gfit (available from Eckhard Bill) and the software package EasySpin (version 5.1.9) as implemented in Matlab.[1] An average of 50% reduction in the $S = {}^{3}/_{2}$ E_0 signal was found for turnover samples, based upon both relative intensity of the g_1 feature at $g = 4.3$ and spin-integration area (Figure S1-1).

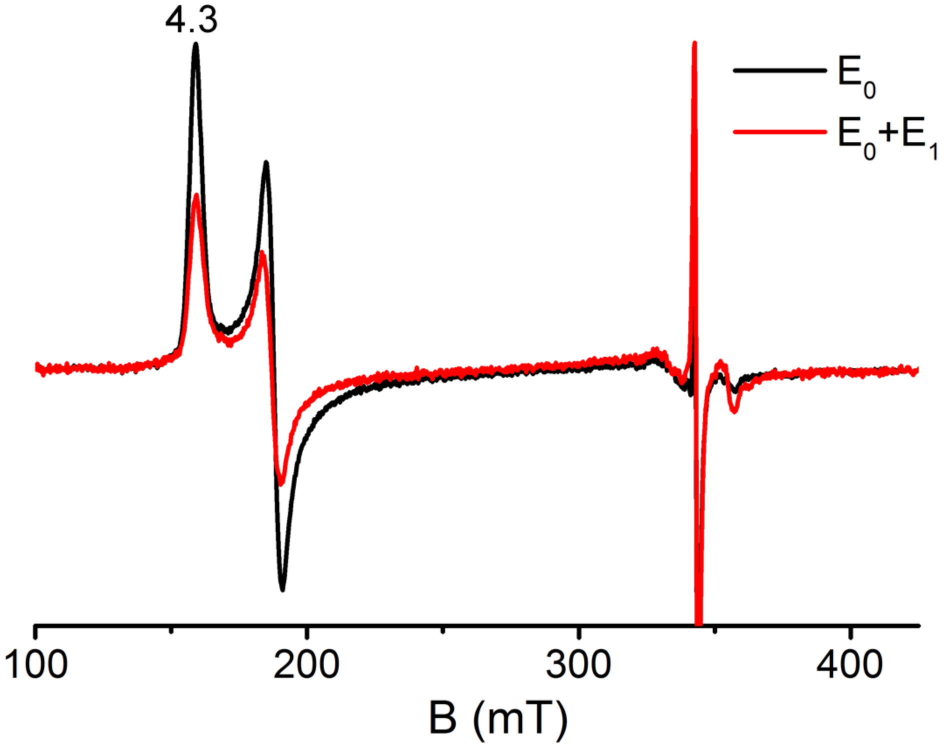

Figure S1-1. X-band EPR spectra of the resting (E_0, black) and turnover (E_0+E_1, red) states. The large radical signal ~ g = 2 arises from the sample holder itself and is unaffiliated with the MoFe sample.

S2. Acquiring and adjusting degeneracy „*N*“

In EXAFS fitting, *N* represents the degeneracy of a given scattering path. This can be interpreted as the average „coordination number“, or how many of a given scatterer surround the absorbing atom. In a more technical sense, it is the number of identical distinct ways per absorbing atom that the scattering defined by a given path can occur.

The degeneracy „*N*“ can be easily ascertained when crystal structures are available, or sufficient information about the coordination environment of the absorber of interest is already known. If we consider the case of the Mo EXAFS of MoFe, where Mo is the absorber, it is already established that Mo is coordinated by two O, one N, three S, and additionally has three neighboring Fe. If we fit individual scattering paths for each of these neighbors, we have $N_{\text{Mo-O}}$ = 2, $N_{\text{Mo-N}}$ = 1, $N_{\text{Mo-S}}$ = 3, and $N_{\text{Mo-Fe}}$ = 3. As O and N are virtually indistinguishable due to their similar mass, the ability to differentiate these paths depends on how disparate the Mo-O vs. Mo-N distances are. If we consider Mo-O and Mo-N indistinguishable, then simply $N_{\text{Mo-O/N}}$ = 3, $N_{\text{Mo-S}}$ = 3, and $N_{\text{Mo-Fe}}$ = 3.

If we consider the case of Fe in MoFe, things become more complicated as there are 15 unique Fe in the protein which may absorb; while a possible partially occupied 16th mononuclear Fe site has been reported,[2] it is not considered due to the uncertain occupancy of this site, light-atom only coordination, and small contribution to the total spectrum. To obtain N here, we can begin by counting all of the unique scattering paths for each individual Fe. The unique scattering pathways for Fe are Fe-C, Fe-Mo, Fe-S, and Fe-Fe. We can then look at the distribution of these distances to decide whether they can be fit with one single-scattering path, or if multiple are needed. For example, a quick survey of a histogram of Fe-Fe distances (Figure S2-1) shows that there is a major group of short Fe-Fe distances between 2.5 and 3 Å, a second group of longer distances ~ 3.8 Å, and several even longer scatterers. Considering the resolution of EXAFS experiments are typically ~ 0.1-0.2 Å (determined by the equation $\Delta R = 2\pi/\Delta k$, where ΔR is the minimum difference in scattering distance to differentiate scatterers of the same identity, and Δk is the range of k-space utilized in the fitting procedure), we can immediately split the Fe-Fe scattering shell into „short“ and „long“ Fe-Fe scattering paths.

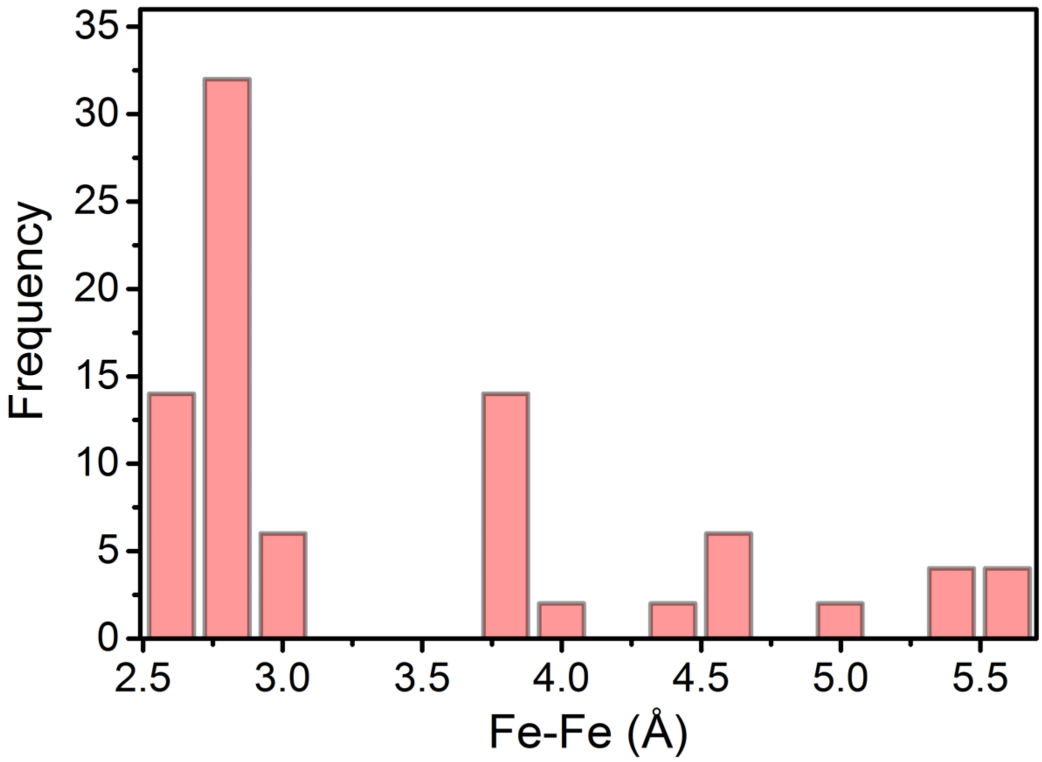

Figure S2-1. Histogram showing all of the Fe-Fe distances below 5.6 Å based on the XRD structure, PDB ID 3U7Q.[3]

We can further examine whether these Fe-Fe paths need to be further differentiated. Looking to Figure S2-2, we see that the long Fe-Fe scatterers arising from the P-cluster are considerably spread over a ~1.7 Å range of distances. To see whether some of these may be feasible to fit, we can determine *N* for a hypothetical path by dividing the total number of scatterers described by that path by the total number of unique Fe (15). For example, a scattering path intended to describe the group of 6 near degenerate Fe-Fe scatterers ~ 4.6 Å would have *N* = 0.4. Considering the large disparity (and therefore low degeneracy) of the Fe-Fe(long) distances arising from the P-cluster, combined with their overall relatively long distance away from the absorber and the limited number of degrees of freedom available in our fitting procedure (determined by the Nyquist criterion, such that the number of degrees of freedom X_{ind} is defined by $X_{ind} = \frac{2(k_{max}-k_{min})(R_{max}-R_{min})}{\pi}$), these scatterers should be omitted in a good EXAFS model of this system. Meanwhile, a scattering path intended to fit the 12 tightly grouped long Fe-Fe distances arising from FeMoco, which are spread by only ~ 0.04 Å, (inset of Figure S2, top) would have *N* = 0.8, and considering the reasonable distance and substantial degeneracy should be able to be reasonably modeled.

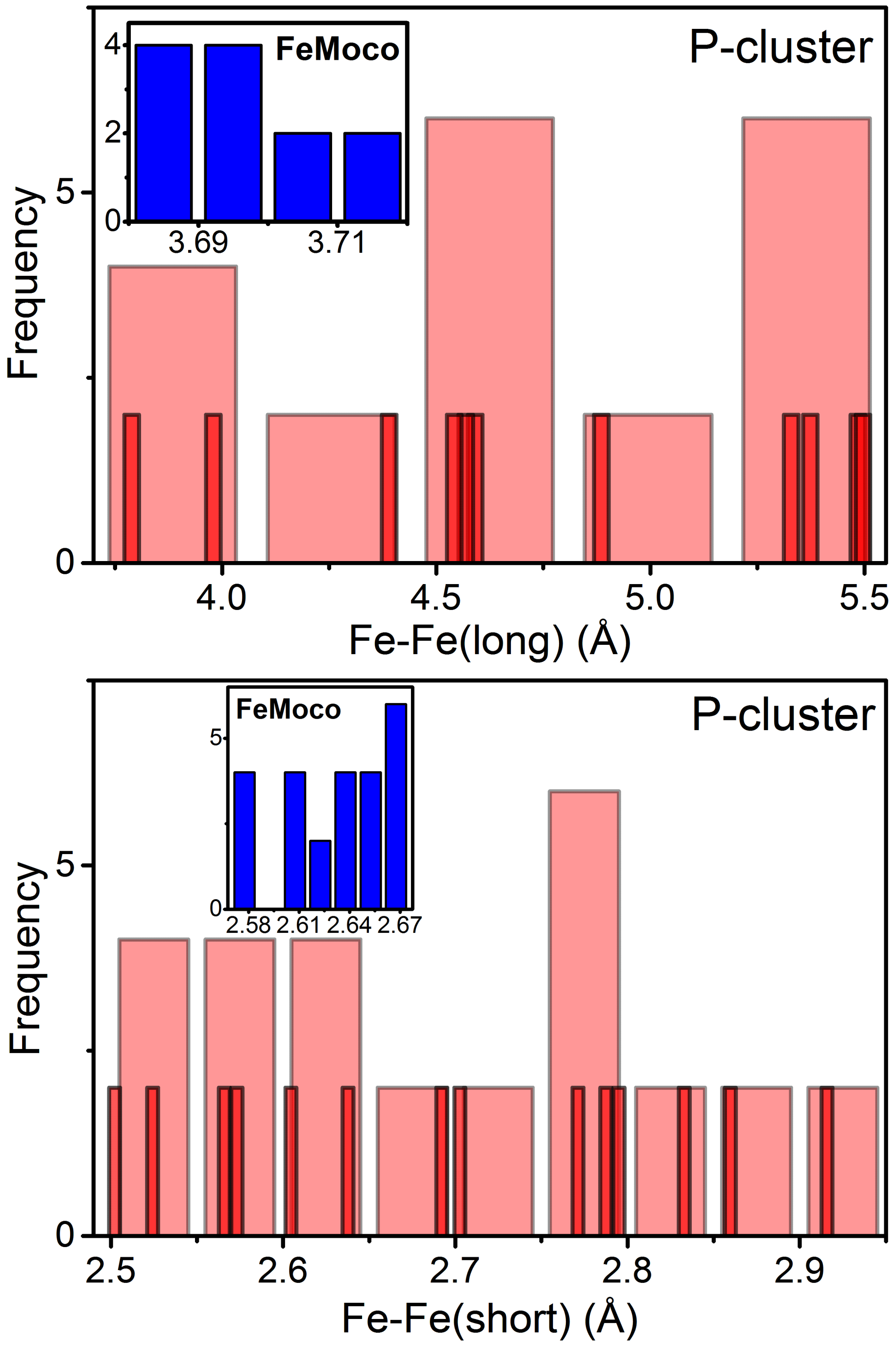

Figure S2-2. Enlarged version of Figure 8 from the main text. Comparison of the (top) long and (bottom) short Fe-Fe bond distances of P-cluster and (insets) FeMoco based on the XRD structure, PDB ID 3U7Q.[3] For the P-cluster distances, both the broader histogram (light red, broad) and exact distances (red, narrow) have been provided. It should be immediately noticeable that the FeMoco presents a significantly more symmetric structure, with long Fe-Fe distances in FeMoco varying only $\Delta_{Fe\text{-}Fe(long)}$ ~ 0.04 Å vs. $\Delta_{Fe\text{-}Fe(long)}$ ~ 0.75-1.7 Å in the P-cluster, and short Fe-Fe distances only $\Delta_{Fe\text{-}Fe(short)}$ ~ 0.1 Å vs. $\Delta_{Fe\text{-}Fe(short)}$ ~ 0.4 Å.

Moving to the short Fe-Fe distances, we have a similar albeit less extreme situation. A scattering path intended to fit all short Fe-Fe distances would need to

encompass a total of 52 Fe-Fe scatterers (24 from FeMoco, 28 from the P-cluster), and would therefore have a degeneracy of N ~3.47. The 24 FeMoco short Fe-Fe scatterers are spread by ~0.1 Å with a median distance of ~ 2.62 Å. Meanwhile, we see that the P-cluster short Fe-Fe distances are spread over a range of ~0.4 Å, with 14 scatterers shorter than 2.7 Å, and 14 longer. Considering this relatively wide range of distances (and that the resolution of our fitting procedure (ΔR) is at most 0.143 Å) it is reasonable that we consider splitting the short Fe-Fe scattering path into two separate paths. Exactly where the split is made is a matter of judgement, and influences the final degeneracy N of each of the new paths. In the present study, we have opted to split at approximately the median of the short Fe-Fe scatterers, 2.7 Å, resulting in a shorter scattering path Fe-Fe (short 1) with 40 scatterers (N ~ 2.53) and a slightly longer scattering path Fe-Fe (short 2) with 14 scatterers (N ~ 0.93).

S3. The feasibility of fitting the Fe-C scatterer

It is now well established that the FeMoco cluster contains a light atom at its core which has been identified as a carbide.[3-6] The knowledge of its existence naturally has implications for any structural modelling of the FeMoco. However, considering C is a very light atom (and thus a weak scatterer) which is coordinated to only 6 of 15 unique irons (giving a degeneracy of N = 0.4), is inclusion of the Fe-C scattering path really required?

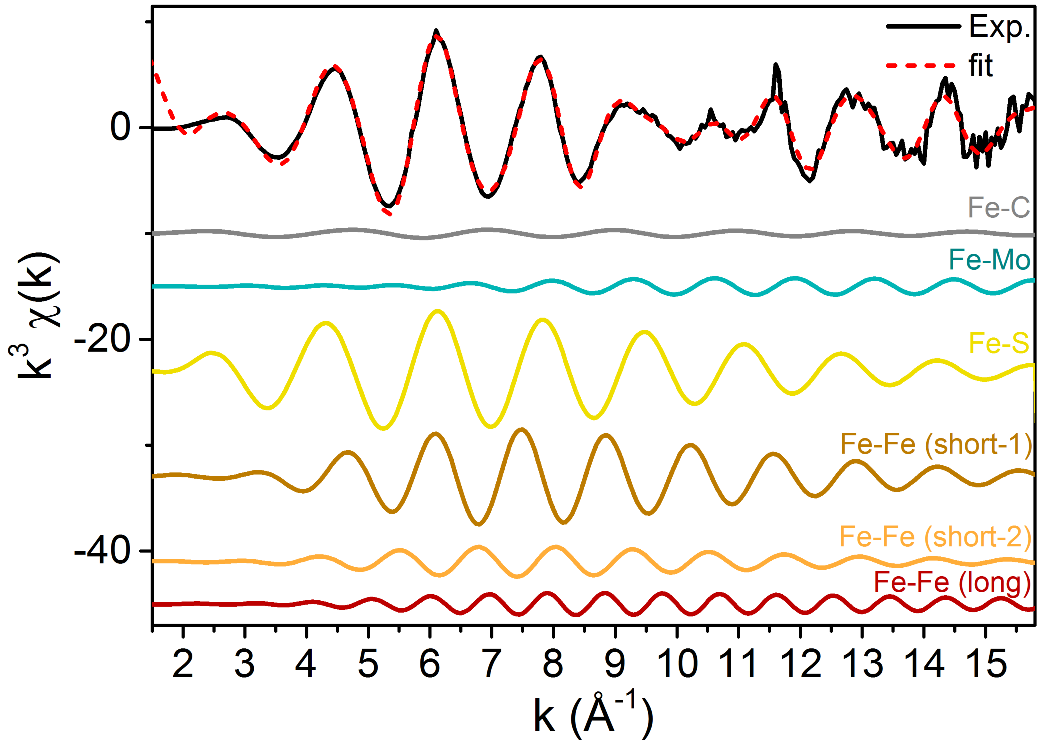

Figure S3-1. k^3-weighted Fe K-edge EXAFS of the resting E_0 state and the 6-component fit to the data (top). Individual scattering path contributions to the total fit in the 6-component model.

Figure S3-1 demonstrates the contributions of each individual scattering path to the 6-component model fit, where the Fe-C scattering path clearly makes only a small contribution to the overall fit. While this may seem insignificant, it is still unclear exactly how modulation of the other paths to make-up for this small contribution in the absence of a Fe-C scattering path would influence the final fit distances. In Figure S3-2, the influence of the presence of the Fe-C scattering path on the final determined distances for the 6-component and 5-component models is shown. Namely, this figure displays the differences in the fit distances for the all other scattering pathways when the Fe-C scattering path is not included. While some very slight modulation occurs, all of these changes are within the error of the fit.

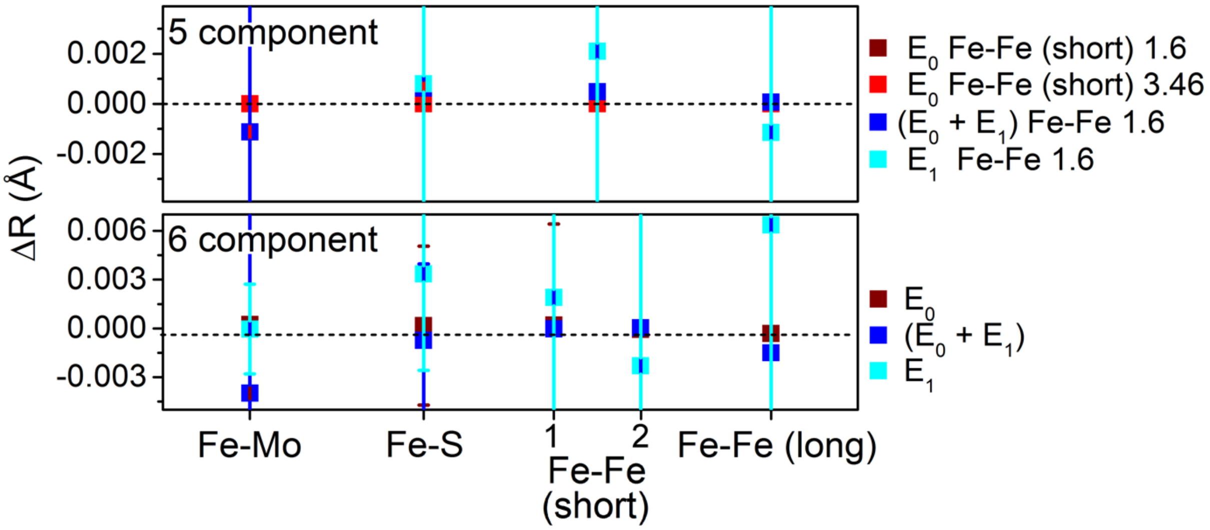

Figure S3-2. Differences in fit distances of the Fe K-edge EXAFS between models including/excluding the Fe-C scattering path. Fe-Fe denotes the Fe-Fe(short) paths, with Fe-Fe(short 1) to the left, and Fe-Fe(short 2) to the right.

The exact parameters for each scattering path, as well as final goodness of fit parameters for the inclusion/exclusion of the Fe-C scatterer, are provided in Table S5-2. On the basis of these statistics, as well as the stark demonstrations provided in Figures S3-1 and S3-2, it is clear that fitting of the Fe-C scattering path in the Fe K-edge EXAFS has a trivial influence on the overall fit. We conclude by acknowledging that its existence sets an *a priori* precedence for including this scattering path when modelling the Fe EXAFS of MoFe, but that its small magnitude and very, very minor influence on the overall fit imply any conclusions based on the fit parameters of this scattering path should be avoided.

S4. Why is E_0 important?

The parameter E_0 (not be confused with the enzyme resting state E_0) is often referred to as the edge energy, reference energy, or even as the inner potential of the absorber of interest. In a formal sense, this parameter functions as the energy which can eject a photoelectron with zero final momentum from a given electronic shell (in the case of K-edge XAS, the 1s). Since numerous electronic processes can occur at the K-edge, including shake-up and shake-down transitions, this parameter becomes difficult to precisely determine. Therefore, it is usually necessary to fit E_0 when modeling EXAFS. E_0 functions to define at what point the EXAFS region begins, as can be seen from equation 1:

$$k(E) = \frac{1}{\hbar}\sqrt{2m_e(E - E_0)} \tag{1}$$

where $\hbar$ is the reduced Planck's constant, E is a given incident energy, and m_e is the mass of an electron. Since E_0 determines the beginning of the EXAFS region, it should also be intuitive that the value of E_0 lies in neither the pre-edge region or EXAFS regions; instead, it should correspond to some point along the edge, and possibly up to the white-line region.

In the case of a single absorbing atom, there can only be one E_0 for all scattering paths. This is because E_0 is defined as a characteristic of the absorber itself, and is independent of the scattering path. If a different E_0 is provided for each scattering path, a change in phase of these paths relative to one another will occur. While doing this may provide fits that appear statistically reasonable, the determined distances are unphysical and can carry major errors. This is demonstrated in Figure S4-1/Table S4-1 for the $MoCl_3$ttcn complex, which consists of Mo coordinated by three S and three Cl. Here, we can see that while using drastically different values of E_0 for each path provides slightly better statistics in the fit, it results in the exchange of the fit Mo-Cl and Mo-S distances, which is chemically unreasonable.

Some samples may contain multiple metals of the same identity (such as Fe in MoFe in this study). If several absorbers contribute to the same spectrum but exist in drastically different chemical environments or oxidation states (for example, 4-sulfur coordinated Fe(II) vs. an Fe(IV)-oxo), it may be possible to model each site individually within a single set of data, if the data is of sufficiently high quality. Using the hypothetical example, it would then be prudent to use one value of E_0 for scattering

pathways involving one of the iron sites, and another E_0 for those involving the other. However, these values should still remain within a reasonable range of one another, on the order of the change in the edge position (usually with 1-4 eV).

In the case of MoFe, only one unique Mo center exists, and it is known that all Fe centers exist in either the Fe(II) or Fe(III) oxidation state, all predominately ligated by either cysteine or sulfide ligation. Therefore, only a single E_0 should be applied to all scattering paths when fitting either the Mo or Fe K-edge EXAFS. Furthermore, as no shift is observed in the position of the edge upon reduction to E_1, both the E_0 and E_1 states should have the same (or very similar) E_0.

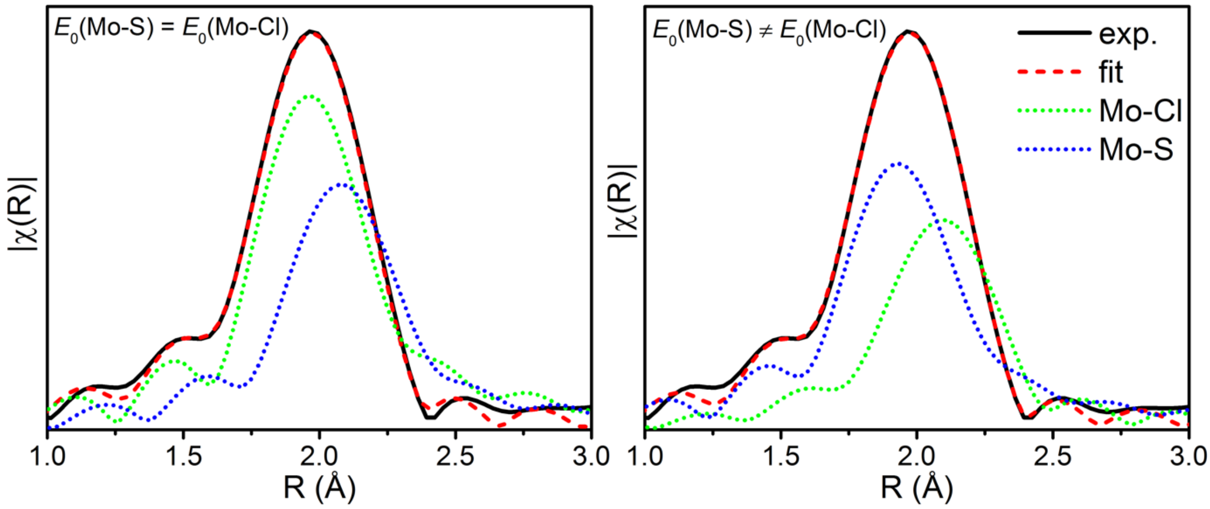

Figure S4-1. Mo K-edge FT EXAFS of $MoCl_3$ttcn, comparing the influence of using separate values of E_0 for each path (right), versus using an identical E_0 for each path (left).

Table S4-1. Comparison of the influence of using separate values of E_0 for each path, versus using an identical E_0 for each path in the $MoCl_3$ttcn complex when fitting the Mo K-edge EXAFS. All fits were performed using a k-range of 3.0-12.5 $Å^{-1}$ and an R-range of 1.0-2.5 Å, along with a fixed S_0^2 = 1.05.

Model	Path	N	E_0 (eV)	σ^2 (10^{-3} $Å^2$)	R (Å)	R-factor
3Cl/3S	Cl	3	20011.33	1.00	2.40	0.0032
	S	3		1.79	2.53	
3Cl/3S	Cl	3	20018.99	3.87	2.52	0.0026
	S	3	20002.96	1.69	2.40	

S5. Supplementary XAS Figures & Tables

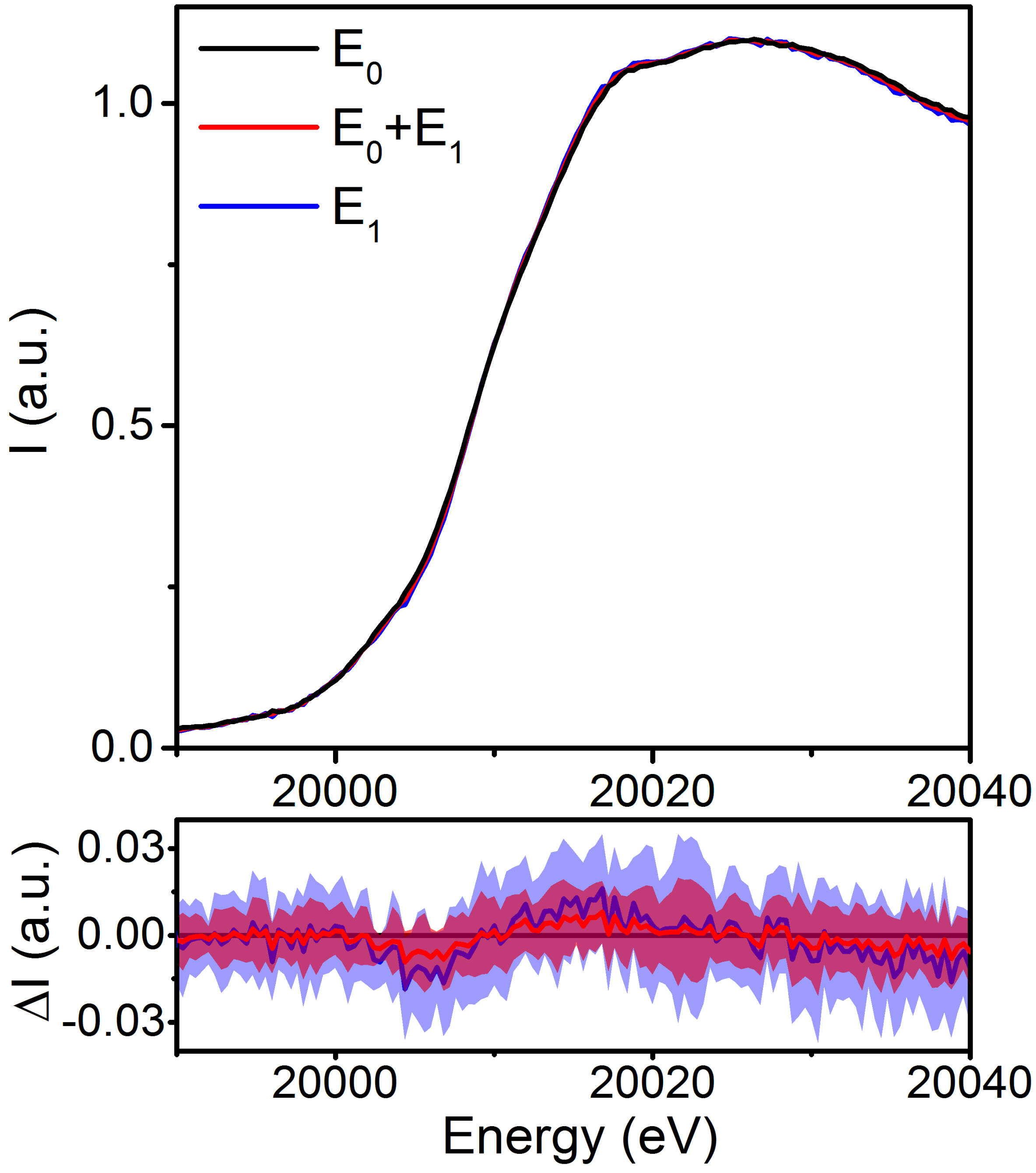

Figure S5-1. (top) Mo K-edge XAS spectra of resting (E_0, black), turnover (E_0+E_1, red) and pure E_1 (blue) MoFe. (bottom) Differential N2ase Mo XAS spectra of E_1 – E_0 (blue) and (E_0+E_1) - E_0 (red). Standard deviations for the difference spectra are plotted.

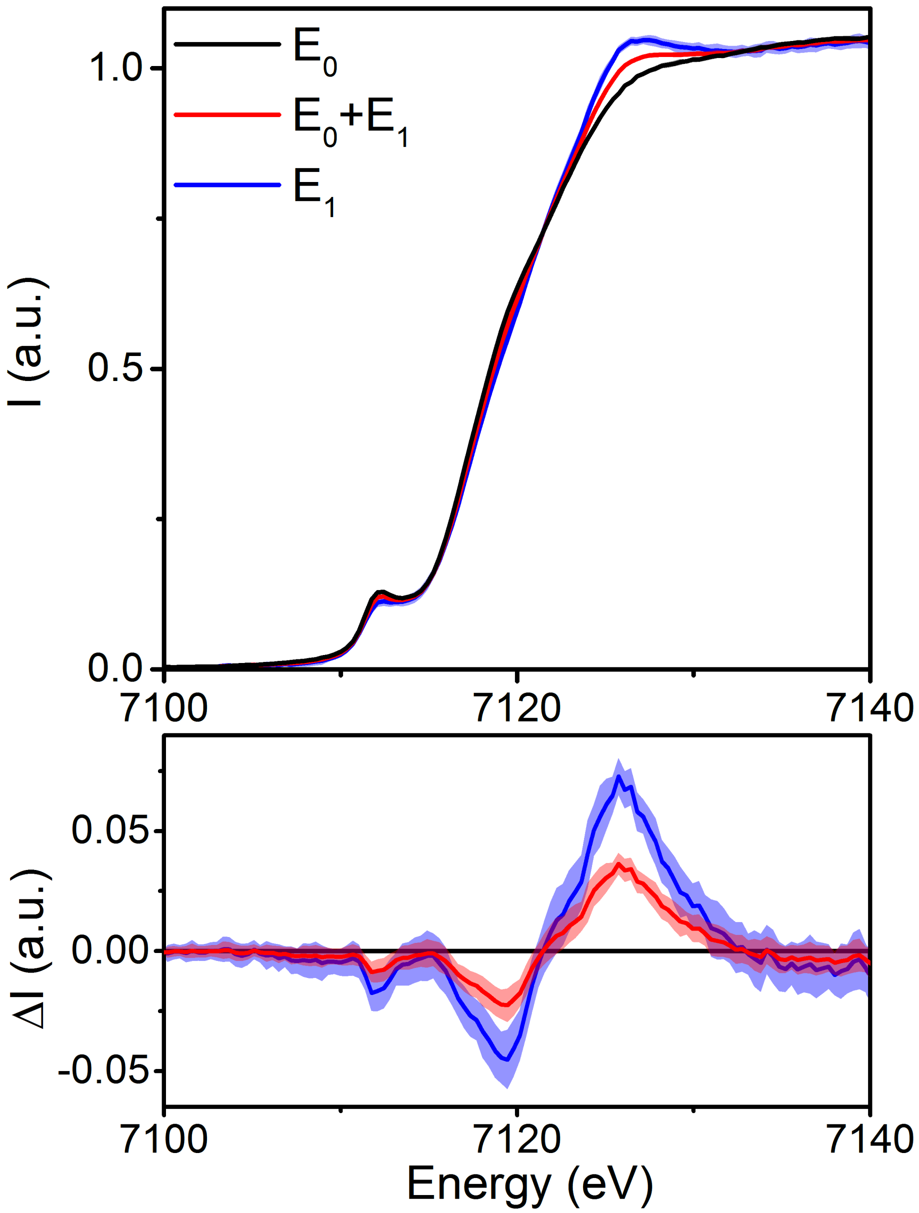

Figure S5-2. (top) Fe K-edge XAS spectra of resting (E_0, black), turnover (E_0+E_1, red) and pure E_1 (blue) Mo Fe. (bottom) Differential N2ase Mo XAS spectra of E_1 – E_0 (blue) and (E_0+E_1) - E_0 (red), demonstrating the changes which occur in these spectra upon native reduction. Standard deviations for both XAS and difference spectra are displayed. The spectra exhibit a pre-edge feature at 7112.8 eV and an inflection in the rising edge at 7118.4 eV. The pre-edge feature arises from the 1s $\rightarrow$ 3d transition, which, while formally dipole forbidden, gains intensity through T_d-symmetry allowed 3d-4p mixing.[7-9] Upon reduction, decreases in intensity at the pre-edge and rising edge are followed by an increased white-line intensity.

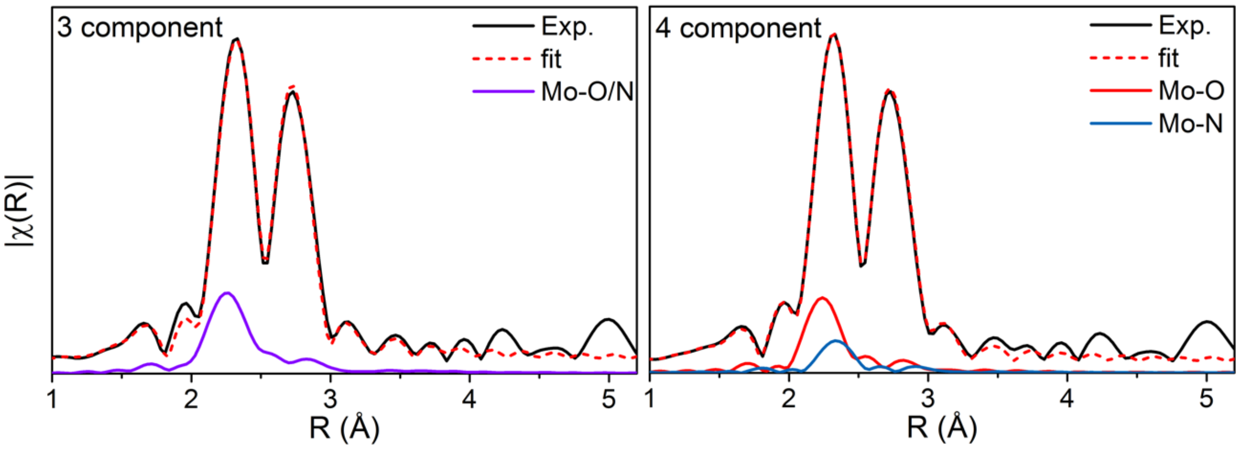

Figure S5-3. Comparison the |FT| space fits of the Mo K-edge EXAFS for the (left) 3-component and (right) 4-component models. The red dashed line represents the total fit, including all scattering paths. The spectra have been phase shifted for the Mo-S scattering path.

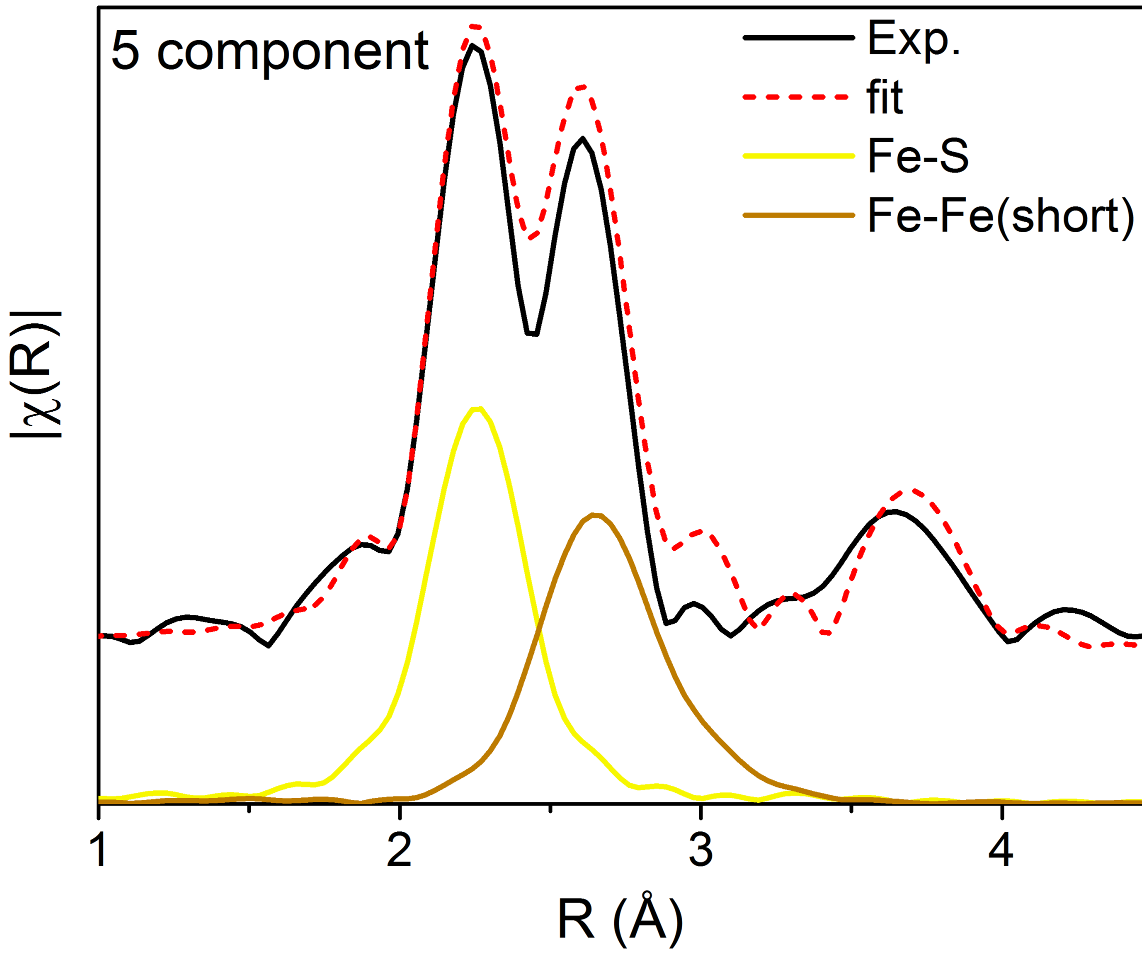

Figure S5-4. Comparison the |FT| space fits and Fe-S and Fe-Fe (short) scattering paths of the Fe K-edge EXAFS for the 5-component, $N_{\text{Fe-Fe(short)}}$ = 3.46 model. The red dashed line represents the total fit, including all scattering paths (and not only those additionally displayed). The spectrum has been phase shifted for the Fe-S scattering path.

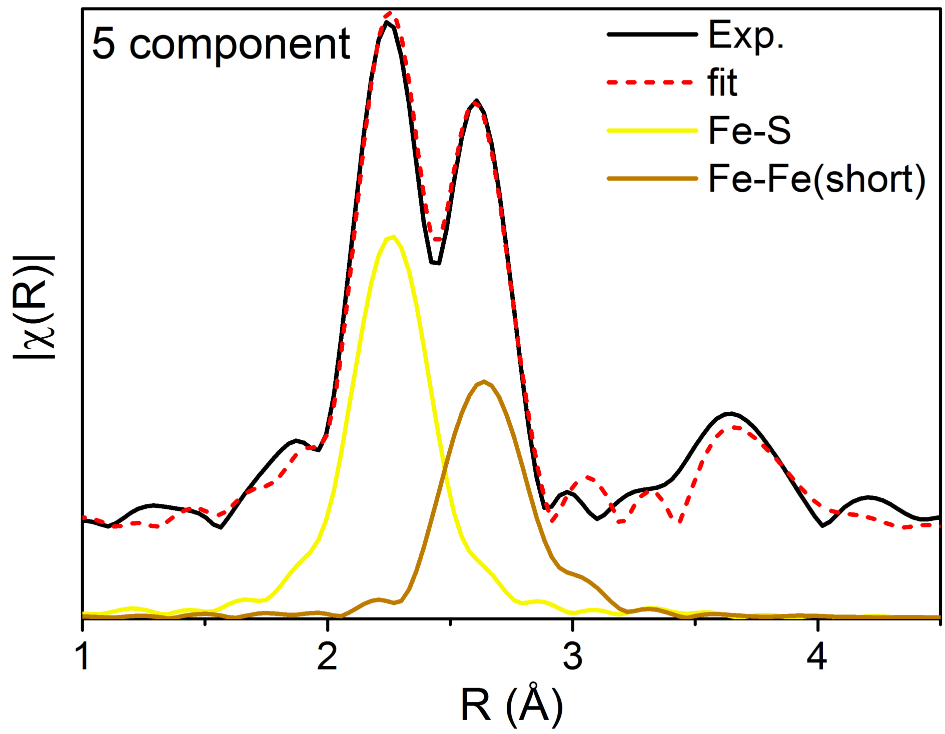

Figure S5-5. Comparison the |FT| space fits and Fe-S and Fe-Fe(short) scattering paths of the Fe K-edge EXAFS for the 5-component, $N_{Fe\text{-}Fe(short)}$ = 1.65 model. The red dashed line represents the total fit, including all scattering paths (and not only those additionally displayed). The spectrum has been phase shifted for the Fe-S scattering path.

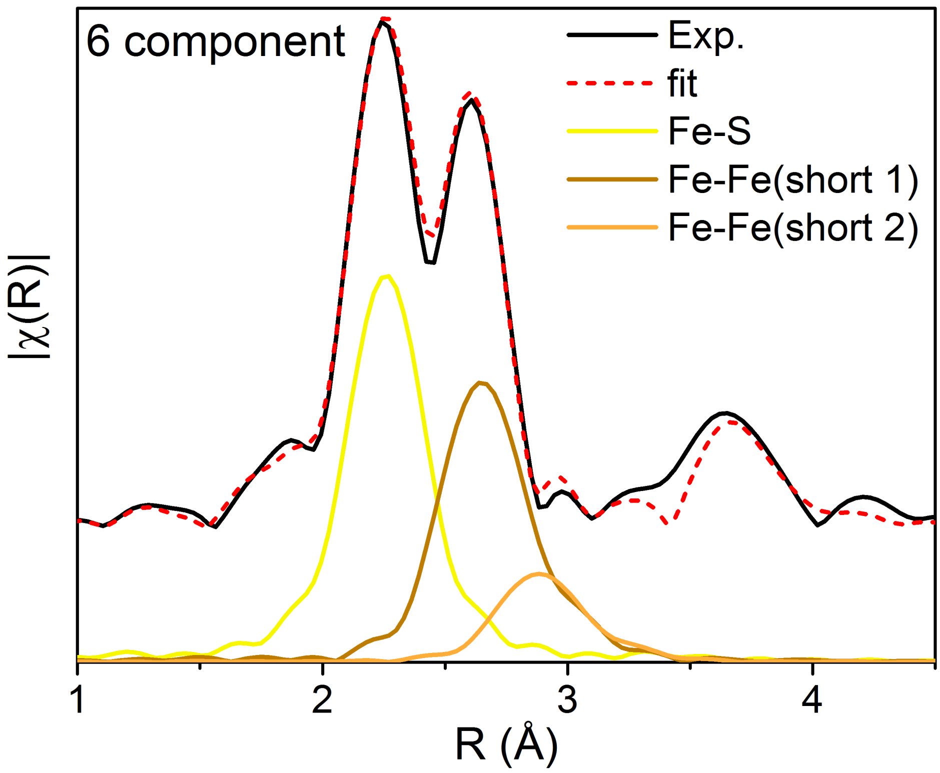

Figure S5-6. Comparison the |FT| space fits and Fe-S, Fe-Fe (short 1), and Fe-Fe (short 2) scattering paths of the Fe K-edge EXAFS for the 6-component, $N_{Fe\text{-}Fe(short\ 1)}$ = 2.53 & $N_{Fe\text{-}Fe(short\ 2)}$ = 0.93 model. The red dashed line represents the total fit, including all scattering paths (and not only those additionally displayed). The spectrum has been phase shifted for the Fe-S scattering path.

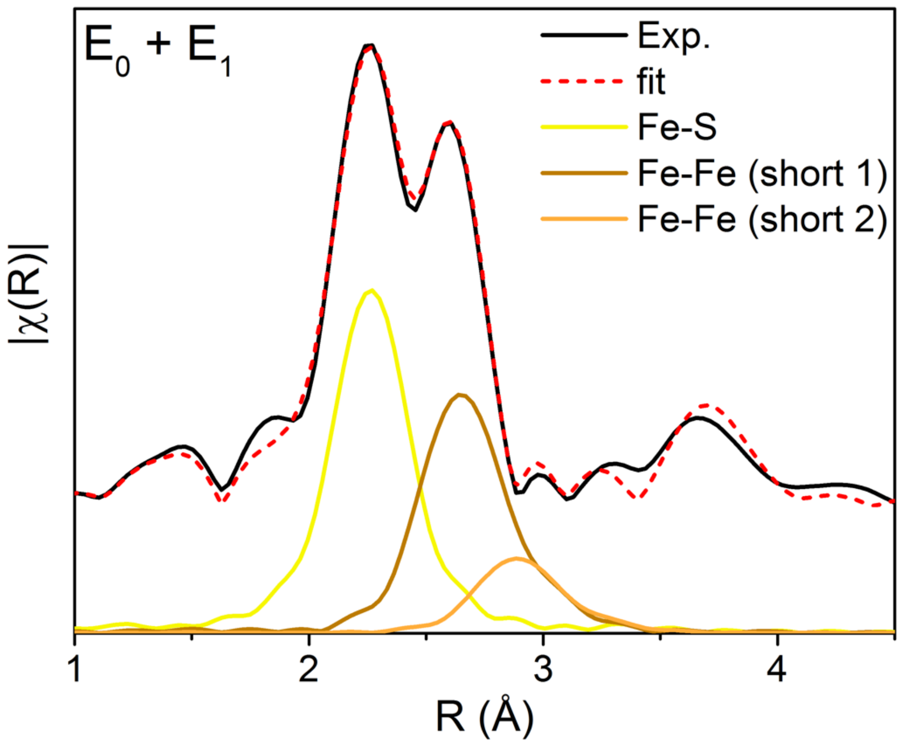

Figure S5-7. Comparison the |FT| space Fe-S, Fe-Fe(short 1), and Fe-Fe(short 2) scattering paths of the Fe K-edge EXAFS for the 6-component model as applied to the turnover E_0+E_1 state. The red dashed line represents the total fit, including all scattering paths (and not only those additionally displayed). The spectra have been phase shifted for the Fe-S scattering path.

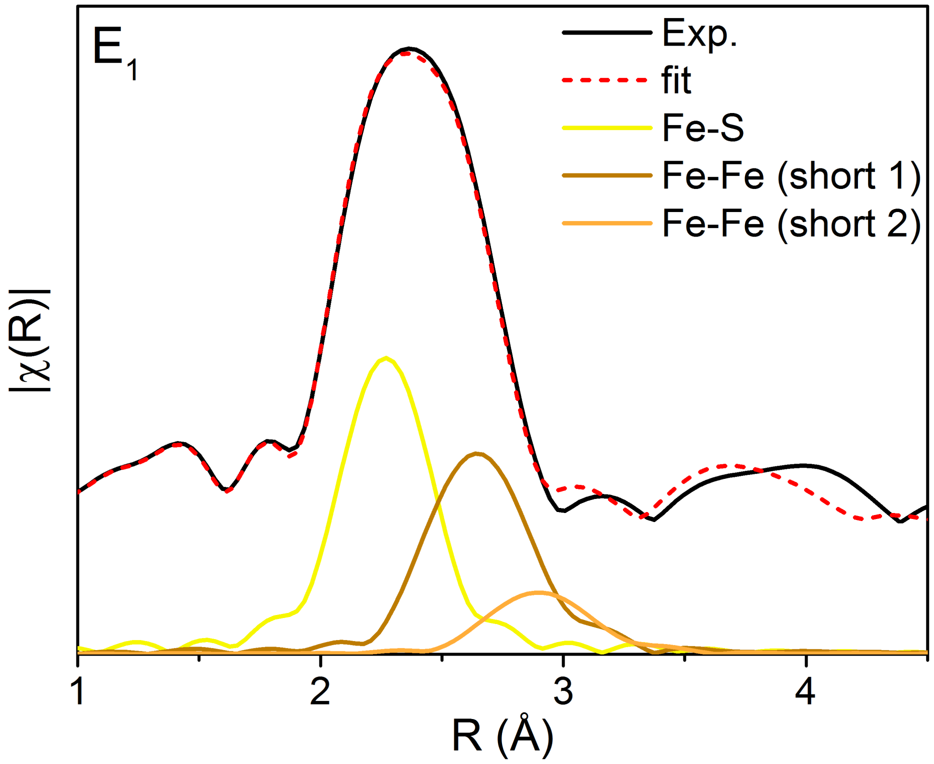

Figure S5-8. Comparison the |FT| space Fe-S, Fe-Fe(short 1), and Fe-Fe(short 2) scattering paths of the Fe K-edge EXAFS for the 6-component model as applied to the E_1 state. The red dashed line represents the total fit, including all scattering paths (and not only those additionally displayed). The spectra have been phase shifted for the Fe-S scattering path.

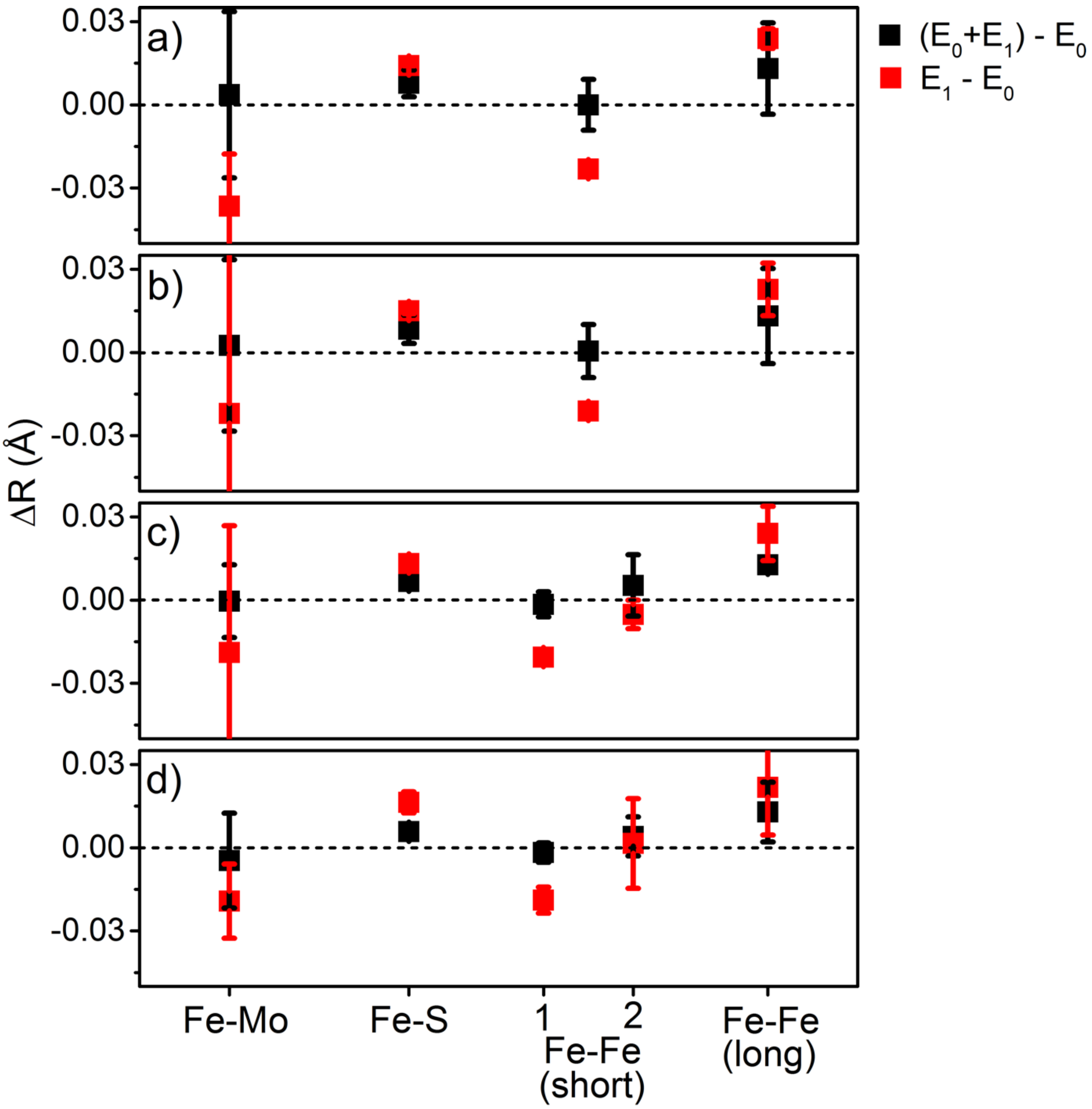

Figure S5-9. Comparison of variation in determined bond distances from the resting E_0 state in the **a**) 4-component (reduced Fe-Fe(short) *N*, no C), **b**) 5-component (reduced Fe-Fe(short) *N*), **c**) 5-component (split Fe-Fe(short), no C), and **d**) 6-component models applied to the Fe EXAFS. Distances are calculated by subtraction of the fit E_0 distances from those of the turnover (E_0+E_1) and E_1 fits. The differences in the Fe-Fe(short) distances in c) and d) are split such that Fe-Fe(short 1) is to the left, and Fe-Fe(short 2) to the right.

Table S5-1. Comparison of the fit parameters of the 3-component and 4-component Mo K-edge EXAFS models.

**Mo feff fits, E_0 = 20013.895 eV**							
Sample	Path = 3	*N*	S_0^2	σ^2 (10^{-3} Å^2)	R (Å)	*k* (Å^{-1})	R-factor
Resting	O/N	3		4.20 (1.01)	2.217 (0.009)	2 – 16.5	
	S	3	1	2.42 (0.30)	2.362 (0.003)	min. ΔR	0.0061
	Fe-short	3		3.25 (0.18)	2.689 (0.002)	0.108	
Turnover	O/N	3		3.52 (1.01)	2.221 (0.009)	2 – 16.5	
(E_0 + E_1)	S	3	1	2.57 (0.36)	2.361 (0.004)	min. ΔR	0.0065
	Fe-short	3		3.24 (0.19)	2.688 (0.002)	0.108	
Turnover	O/N	3		2.34 (1.31)	2.221 (0.010)	2 – 16	
(E_1)	S	3	1	2.81 (0.68)	2.365 (0.005)	min. ΔR	0.0117
	Fe-short	3		3.16 (0.29)	2.697 (0.003)	0.112	

Sample	Path = 4	*N*	S_0^2	σ^2 (10^{-3} Å^2)	R (Å)	*k* (Å^{-1})	R-factor
Resting	O	2	1	2.36 (0.67)	2.198 (0.007)	1.6 – 16.4 min. ΔR 0.106	0.0024
	N	1		1.72 (1.09)	2.303 (0.011)		
	S	3		2.51 (0.20)	2.365 (0.004)		
	Fe-short	3		3.18 (0.11)	2.693 (0.001)		
Turnover (E_0 + E_1)	O	2	1	2.53 (0.68)	2.197 (0.006)	1.6 – 16.4 min. ΔR 0.106	0.0024
	N	1		2.01 (1.35)	2.309 (0.011)		
	S	3		2.24 (0.16)	2.361 (0.002)		
	Fe-short	3		3.16 (0.11)	2.688 (0.001)		
Turnover (E_1)	O	2	1	0.93 (0.77)	2.202 (0.011)	1.6 – 16 min. ΔR 0.109	0.0108
	N	1		3.00 (0.02)	2.302 (0.006)		
	S	3		2.57 (0.52)	2.367 (0.006)		
	Fe-short	3		3.18 (0.26)	2.697 (0.003)		

Table S5-2. Fe K-edge EXAFS fit parameters of the 4-component model using a reduced degeneracy *N* for the short Fe-Fe path and no Fe-C scattering path (no Fe-C analog of 5-component model w/ reduced N).

**Fe feff fits, E_0 = 7118.03 eV**							
Sample	Path = 4	*N*	S_0^2	σ^2 (10^{-3} Å^2)	R (Å)	*k* (Å^{-1})	R-factor
Resting	Mo	0.2	0.9	2.00 (2.07)	2.700 (0.026)	2 – 15.8 min. ΔR 0.114	0.0252
	S	3.6		5.66 (0.39)	2.269 (0.003)		
	Fe-short	1.65		4.26 (0.96)	2.612 (0.006)		
	Fe-long	0.8		2.00 (1.08)	3.691 (0.012)		
Turnover (E_0 + E_1)	Mo	0.2	0.9	1.14 (1.16)	2.704 (0.015)	2 – 15.8 min. ΔR 0.114	0.0203
	S	3.6		6.30 (0.42)	2.277 (0.003)		
	Fe-short	1.65		5.56 (0.94)	2.612 (0.007)		
	Fe-long	0.8		2.01 (1.00)	3.704 (0.011)		
Turnover (E_1)	Mo	0.2	0.9	7.00 (0.00)	2.664 (0.007)	2 – 13 min. ΔR 0.143	0.0099
	S	3.6		7.28 (0.42)	2.283 (0.003)		
	Fe-short	1.65		5.48 (0.49)	2.588 (0.004)		
	Fe-long	0.8		4.37 (1.68)	3.715 (0.016)		

Table S5-3. Fe K-edge EXAFS fit parameters of the 5-component model using a reduced degeneracy *N* for the short Fe-Fe path.

Fe feff fits, E_0 = 7118.03 eV

Sample	Path = 5	*N*	S_0^2	σ^2 (10^{-3} Å^2)	R (Å)	*k* (Å^{-1})	R-factor
Resting	C	0.4		2.00 (0.23)	1.910 (0.036)		
	Mo	0.2		2.00 (2.08)	2.700 (0.026)	2 – 15.8	
	S	3.6	0.9	5.66 (0.39)	2.269 (0.003)	min. ΔR	0.0224
	Fe-short	1.65		4.26 (0.97)	2.611 (0.006)	0.114	
	Fe-long	0.8		2.00 (0.05)	3.691 (0.012)		
Turnover	C	0.4		5.00 (0.00)	1.949 (0.065)		
(E_0 + E_1)	Mo	0.2		1.15 (1.26)	2.703 (0.016)	2 – 15.8	
	S	3.6	0.9	6.19 (0.44)	2.277 (0.004)	min. ΔR	0.0201
	Fe-short	1.65		5.51 (1.02)	2.612 (0.007)	0.114	
	Fe-long	0.8		2.00 (1.08)	3.705 (0.012)		
Turnover	C	0.4		5.00 (0.00)	2.009 (0.073)		
(E_1)	Mo	0.2		7.00 (0.00)	2.679 (0.123)	2 – 13	
	S	3.6	0.9	7.22 (0.59)	2.276 (0.004)	min. ΔR	0.0130
	Fe-short	1.65		5.39 (1.48)	2.591 (0.006)	0.143	
	Fe-long	0.8		4.87 (2.37)	3.714 (0.022)		

Table S5-4. Fe K-edge EXAFS fit parameters of the 5-component model as applied to the resting state E_0 EXAFS, along with the 4-component analog in which the Fe-C scattering path is not considered.

Fe feff fits, E_0 = 7118.03 eV

Sample	Path = 4	*N*	S_0^2	σ^2 (10^{-3} Å^2)	R (Å)	*k* (Å^{-1})	R-factor
Resting	Mo	0.2		1.00 (1.53)	2.702 (0.017)	2 – 15.8	
	S	3.6	0.9	5.90 (0.65)	2.265 (0.006)	min. ΔR	0.0678
	Fe-short	3.64		8.00 (0.01)	2.618 (0.008)	0.114	
	Fe-long	0.8		0.87 (1.25)	3.690 (0.015)		

Sample	Path = 5	*N*	S_0^2	σ^2 (10^{-3} Å^2)	R (Å)	*k* (Å^{-1})	R-factor
Resting	C	0.4		1.00 (4.84)	1.907 (0.049)		
	Mo	0.2		1.00 (1.68)	2.702 (0.019)	2 – 15.8	
	S	3.6	0.9	5.90 (0.72)	2.265 (0.006)	min. ΔR	0.0684
	Fe-short	3.64		8.00 (0.01)	2.618 (0.008)	0.114	
	Fe-long	0.8		0.87 (1.34)	3.690 (0.016)		

Table S5-5. Fe K-edge EXAFS fit parameters of the 5-component model using split short Fe-Fe paths and no Fe-C scattering path (a no Fe-C analog of 6-component model).

**Fe feff fits, E_0 = 7118.03 eV**							
Sample	Path = 5	*N*	S_0^2	σ^2 (10^{-3} Å^2)	R (Å)	*k* (Å^{-1})	R-factor
Resting	Mo	0.2	0.9	2.00 (0.26)	2.683 (0.048)	2 – 15.8 min. ΔR 0.114	0.018
	S	3.6		5.63 (0.43)	2.267 (0.004)		
	Fe-short	2.53		5.73 (1.27)	2.622 (0.004)		
	Fe-short	0.93			2.855 (0.016)		
	Fe-long	0.8		2.00 (0.59)	3.691 (0.013)		
Turnover (E_0 + E_1)	Mo	0.2	0.9	2.00 (2.45)	2.683 (0.035)	2 – 15.8 min. ΔR 0.114	0.0153
	S	3.6		6.22 (0.44)	2.274 (0.004)		
	Fe-short	2.53		6.61 (1.27)	2.620 (0.009)		
	Fe-short	0.93			2.860 (0.027)		
	Fe-long	0.8		2.00 (0.98)	3.704 (0.011)		
Turnover (E_1)	Mo	0.2	0.9	5.00 (0.00)	2.664 (0.002)	2 – 13 min. ΔR 0.143	0.0081
	S	3.6		7.54 (0.76)	2.280 (0.005)		
	Fe-short	2.53		7.47 (0.77)	2.601 (0.005)		
	Fe-short	0.93			2.849 (0.021)		
	Fe-long	0.8		5.05 (2.45)	3.715 (0.022)		

Table S5-6. Fe K-edge EXAFS fit parameters of the 6-component model. Due to the similar scattering behavior of the two Fe-short scattering paths, the σ^2 of these two paths were set to be equivalent during fitting.

**Fe feff fits, E_0 = 7118.03 eV**							
Sample	Path = 6	*N*	S_0^2	σ^2 (10^{-3} Å^2)	R (Å)	*k* (Å^{-1})	R-factor
Resting	C	0.4	0.9	2.00 (0.82)	1.911 (0.034)	2 – 15.8 min. ΔR 0.114	0.0142
	Mo	0.2		2.00 (0.62)	2.683 (0.013)		
	S	3.6		5.59 (0.44)	2.267 (0.002)		
	Fe-short	2.53		5.72 (1.30)	2.622 (0.003)		
	Fe-short	0.93			2.854 (0.012)		
	Fe-long	0.8		1.99 (0.09)	3.691 (0.010)		
Turnover (E_0 + E_1)	C	0.4	0.9	2.00 (2.81)	1.928 (0.024)	2 – 15.8 min. ΔR 0.114	0.0093
	Mo	0.2		2.00 (2.27)	2.679 (0.010)		
	S	3.6		6.10 (0.39)	2.273 (0.001)		
	Fe-short	2.53		6.45 (1.11)	2.620 (0.003)		
	Fe-short	0.93			2.858 (0.010)		
	Fe-long	0.8		1.99 (0.85)	3.704 (0.007)		
Turnover (E_1)	C	0.4	0.9	2.00 (0.35)	1.992 (0.029)	2 – 13 min. ΔR 0.143	0.0079
	Mo	0.2		5.00 (0.00)	2.664 (0.001)		
	S	3.6		7.12 (0.00)	2.283 (0.003)		
	Fe-short	2.53		7.60 (0.91)	2.603 (0.003)		
	Fe-short	0.93			2.856 (0.010)		
	Fe-long	0.8		4.66 (2.10)	3.713 (0.014)		

S6. Computational Details

S6.1. Labelling scheme of the broken-symmetry states & Selection of broken-symmetry states for investigation

The FeMoco cluster contains 8 metal centers with unpaired electrons. The E_0 state is calculated assuming the charge state $[MoFe_7S_9C]^{1-}$ (as has been established in previous work[10, 11]) that can be written in terms of a formal oxidation state distribution of $Mo^{III}4Fe^{III}3Fe^{II}$. The Mo^{III} oxidation state has been previously determined by Mo HERFD-XAS and theoretical calculations.[12] In E_1 the formal oxidation state distribution changes to $Mo^{III}3Fe^{III}4Fe^{II}$. To find the lowest energy antiferromagnetically coupled broken-symmetry spin states we first calculate high-spin states (where all spins are ferromagnetically aligned), M_S = $^{35}/_2$ (E_0) and M_S = $^{34}/_2$ (E_1). From these high-spin states, the spin densities at selected Fe atoms are “flipped” and the SCF (self-consistent field) is then reconverged to find the lowest energy solution corresponding to a specific M_S value. For E_0, the M_S = $^3/_2$ solutions are lowest in energy, consistent with the experimental S = $^3/_2$ spin state. As the experimental spin state of E_1 is ≥ 1,[13] both the M_S = 1 and M_S = 2 broken symmetry state solutions were initially probed (Figure S6.1-1).

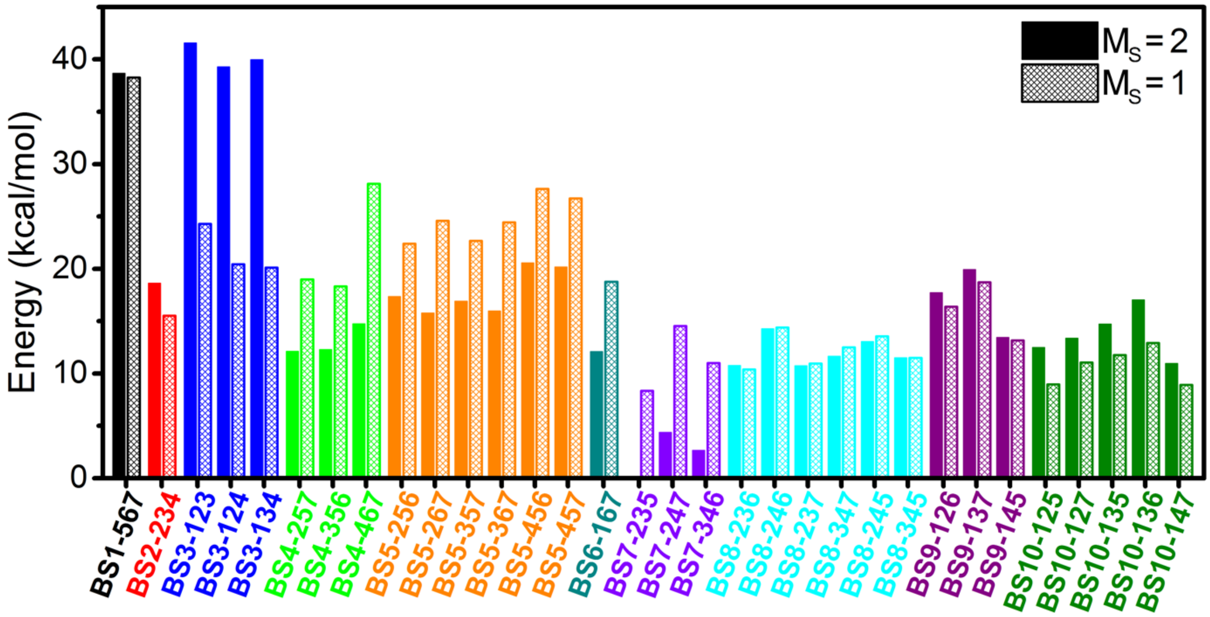

Figure S6.1-1. Relative energies (w.r.t. BS7-235) of possible broken-symmetry solutions from the addition of a single electron to the BS-235 optimized E_0 structure of the FeMoco cluster to form an unrelaxed unprotonated E_1 state. The presented labeling scheme provides first the C_{3v} category of the BS solution, followed by three numbers which describe the BS solution under C_1 symmetry. The numbers denote the “spin-down” Fe ions.

The possible BS solutions of the E_0 state of FeMoco have previously been organized into 10 categories with the assumption that the cluster maintains C_{3v} symmetry.[14] However, as the effective C_{3v} symmetry of the cluster is broken by the ligands and the secondary environment of the protein, all degeneracy within these categories is lifted, resulting in up to 35 different solutions. The same case is true following the addition of one electron to form the E_1 state. The energies of these different solutions for E_1 (in the absence of an added proton) are shown in Figure S6.1-1. From these calculations, we find that all three of the lowest energy solutions fall within the "BS7" category, with S = 2. Any further discussion of these three lowest energy solutions will forgo the label BS7.

The precise couplings of the BS-235 solution of E_0 and three lowest energy BS solutions of E_1 (BS-235, BS-346 and BS-247) are described in Figure S6.1-2. Details regarding the electronic state of E_0 can be found in our past work.[10-12] Briefly, based on localized orbital (Pipek-Mezey method[15]) and structural analyses, our present understanding of E_0 consists of an $Mo^{III}4Fe^{III}3Fe^{II}$ oxidation state distribution in a BS-235 electronic configuration. In this case, Fe2 and Fe3 form a mixed-valent delocalized pair in the $[Fe_4S_3C]$ sub-cubane, while Fe6 and Fe7 are mixed-valence delocalized in the $[MoFe_3S_3C]$ sub-cubane. There is partial delocalization of the paired electron between Fe1 and Fe4, although it is more localized on Fe_1. The BS-247 and BS-346 solutions, which are comparable in energy to the BS-235 solution, are the C_3 rotated equivalents of this description.

In the context of E_1, these three solutions effectively rotate which of two possible Fe in the cluster is reduced (Fe_4 or Fe_5 in BS-235, Fe_2 or Fe_6 in BS-346, and Fe_3 or Fe_7 in BS-247; see Figure S6.1-2). While it is possible that the added electron could be on either of two Fe atoms for a given BS solution, we have found that reduction is always localized to one of the Fe atoms of the $[MoFe_3S_3C]$ sub-cubane (Fe_5 through Fe_7). The localized orbital of the added electron are provided in Figure S6.1-2 for the most favorable BS solution of each of the three possible belt-sulfide protonation sites. Interestingly, localized orbital analysis of these solutions has revealed that the delocalized electron of the spin-down mixed-valent delocalized pair in the $[Fe_4S_3C]$ sub-cubane becomes less delocalized in our E_1 models. While this localization tends to occur at the Fe of the pair which resides closer to either of the Arg residues (Arg 96 or Arg359, Figure S6.4-2), we note that the magnitude of localization/delocalization is dependent on both the size of the QM region and employed exchange-correlation

functional. Tables S6.1-1 to S6.1-3 give the spin populations of all broken-symmetry states calculated for E_0 and E_1. The site of reduction can also be deduced from the change in spin population.

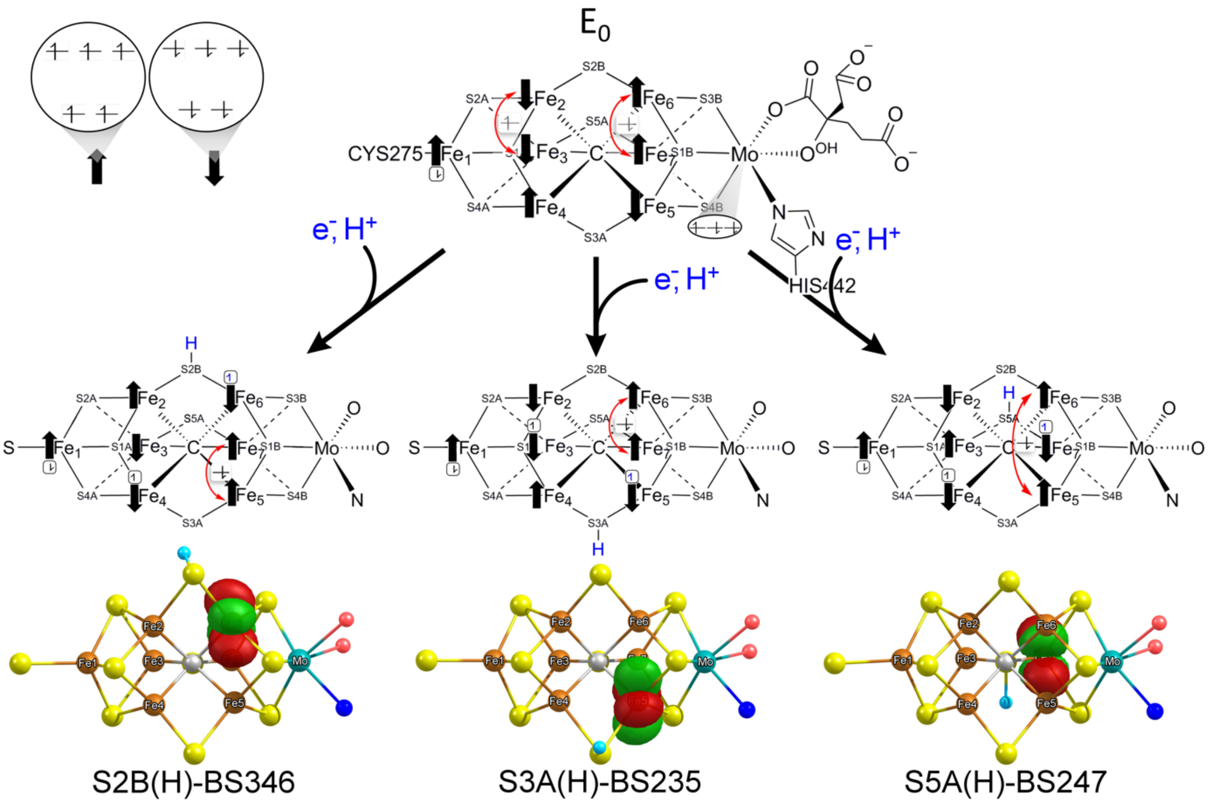

Figure S6.1-2. Spin-coupling of the BS235 solution of the E_0 state, and the three considered BS solutions (BS235, BS346, and BS247) resulting broken symmetry solutions of the E_1 state (demonstrated for the S2B(H)-BS346, S3A(H)-BS235, and S5A(H)-BS247 models) according to localized orbital analysis. The red arrow denotes completely spin-delocalized mixed-valent pairs. In all cases, the additional electron of the E_1 state (blue) most favorably resides on the a) Mo sub-cubane, and b) on the Fe neighboring the protonated S. The localized *d*-orbital associated with the minority-spin electron at Fe6, Fe5 and Fe7, respectively is shown.

Table S6.1-1 Mulliken spin-populations of the metal centers of FeMoco for all homocitrate-protonated models of E_0 and E_1 using the BS-235 solution.

	Metal Center							
N_ε	Mo	Fe1	Fe2	Fe3	Fe4	Fe5	Fe6	Fe7
E_0	-0.54	3.43	-3.21	-3.21	3.30	-3.12	2.98	2.90
$E_0 + e^-$	-0.45	3.42	-3.17	-3.17	3.36	-2.76	3.06	2.94
S2B(H)	-0.36	3.41	-3.14	-3.21	3.37	-2.77	2.99	3.00
S5A(H)	-0.59	3.46	-3.18	-3.21	3.21	-2.51	3.05	2.99
S3A(H)	-0.40	3.41	-3.18	-3.14	3.35	-2.74	3.08	2.90
S1A(H)	-0.43	3.38	-3.06	-3.16	3.22	-2.75	3.12	2.99
S3B(H)	0.22	3.43	-3.18	-3.21	3.34	-3.13	3.17	2.87
Fe6(H)	-0.36	3.40	-2.70	-3.05	3.32	-2.65	2.35	2.96
N_δ	Mo	Fe1	Fe2	Fe3	Fe4	Fe5	Fe6	Fe7
E_0								

$E_0 + e^-$	-0.41	3.41	-3.14	-3.17	3.36	-2.77	3.00	2.94
S2B(H)	-0.35	3.41	-3.15	-3.20	3.37	-2.77	2.98	3.00
S5A(H)	-0.55	3.45	-3.15	-3.20	3.22	-2.51	2.98	2.99
S3A(H)	-0.37	3.41	-3.15	-3.13	3.36	-2.76	3.02	2.90
S1A(H)	-0.42	3.37	-3.01	-3.15	3.21	-2.77	3.08	2.99
S3B(H)	-0.48	3.41	-3.07	-3.12	3.37	-2.69	2.88	2.90
Fe6(H)	-0.36	3.40	-2.68	-3.05	3.32	-2.67	2.31	2.96

Table S6.1-2 Mulliken spin-populations of the metal centers of FeMoco for all homocitrate-protonated models of E_0 and E_1 using the BS-346 solution.

	Metal Center							
N_ε	Mo	Fe1	Fe2	Fe3	Fe4	Fe5	Fe6	Fe7
E_0	-0.26	3.42	3.32	-3.23	-3.20	2.99	-3.12	2.87
$E_0 + e^-$								
S2B(H)	-0.31	3.46	3.30	-3.21	-3.19	3.06	-2.62	2.94
S5A(H)	-0.23	3.45	3.38	-3.14	-2.78	2.90	-3.09	2.92
S3A(H)	-0.24	3.41	3.38	-3.12	-3.20	3.09	-2.69	2.86
S1A(H)	-0.28	3.42	3.25	-3.16	-3.22	3.10	-2.71	2.95
S3B(H)	-0.08	3.43	3.36	-3.20	-3.22	3.00	-2.83	2.95
Fe6(H)	-0.35	3.43	3.24	-3.12	-3.13	3.04	-2.61	2.87
N_δ	Mo	Fe1	Fe2	Fe3	Fe4	Fe5	Fe6	Fe7
E_0								
$E_0 + e^-$	-0.31	3.41	3.38	-3.18	-3.19	3.04	-2.64	2.92
S2B(H)	-0.35	3.44	3.32	-3.21	-3.20	3.06	-2.56	2.93
S5A(H)	-0.26	3.41	3.38	-3.23	-3.11	2.89	-2.58	2.98
S3A(H)	-0.28	3.40	3.37	-3.13	-3.20	3.09	-2.59	2.85
S1A(H)	-0.33	3.41	3.24	-3.16	-3.23	3.09	-2.61	2.95
S3B(H)	-0.15	3.42	3.36	-3.14	-3.18	2.98	-2.76	2.92
Fe6(H)	-0.37	3.43	3.24	-3.13	-3.13	3.04	-2.56	2.88

Table S6.1-3 Mulliken spin-populations of the metal centers of FeMoco for all homocitrate-protonated models of E_0 and E_1 using the BS-247 solution.

	Metal Center							
N_ε	Mo	Fe1	Fe2	Fe3	Fe4	Fe5	Fe6	Fe7
E_0	-0.46	3.42	-3.21	3.32	-3.18	3.00	2.93	-3.05
$E_0 + e^-$								
S2B(H)	-0.36	3.39	-3.16	3.38	-3.19	3.09	2.95	-2.62
S5A(H)	-0.32	3.40	-3.19	3.40	-3.07	2.90	3.03	-2.65
S3A(H)	-0.46	3.44	-3.18	3.28	-3.18	3.07	2.99	-2.55
S1A(H)	-0.38	3.42	-3.08	3.39	-3.15	3.05	2.98	-2.66
S3B(H)	-0.26	3.42	-3.11	3.36	-3.15	2.99	2.98	-2.78
Fe6(H)	-0.36	3.38	-2.76	3.35	-3.06	3.04	2.33	-2.47
N_δ	Mo	Fe1	Fe2	Fe3	Fe4	Fe5	Fe6	Fe7
E_0								
$E_0 + e^-$	-0.47	3.40	-3.14	3.38	-3.16	3.06	2.97	-2.61
S2B(H)	-0.35	3.39	-3.16	3.39	-3.18	3.09	2.94	-2.62
S5A(H)	-0.30	3.41	-3.18	3.40	-3.06	2.89	2.97	-2.66
S3A(H)	-0.44	3.44	-3.16	3.28	-3.18	3.08	2.93	-2.54
S1A(H)	-0.38	3.41	-3.04	3.40	-3.15	3.06	2.92	-2.66
S3B(H)	-0.26	3.41	-3.09	3.37	-3.16	3.00	2.90	-2.77

Fe6(H)	-0.36	3.38	-2.75	3.36	-3.07	3.05	2.29	-2.49

S6.2. Determination of the homocitrate protonation state

The protonation state of the hydroxyl group of homocitrate has direct implications on the structural environment of Mo in the FeMoco cluster. Previous studies have supported that this group is protonated in the E_0 state, and is held hydrogen-bonded to one of the uncoordinated carboxylate groups of the homocitrate (Figure S6.2-1).[10, 16] However, it is unknown whether this remains true for any of the E_n states (n > 0), and necessitates further investigation in the case of E_1.

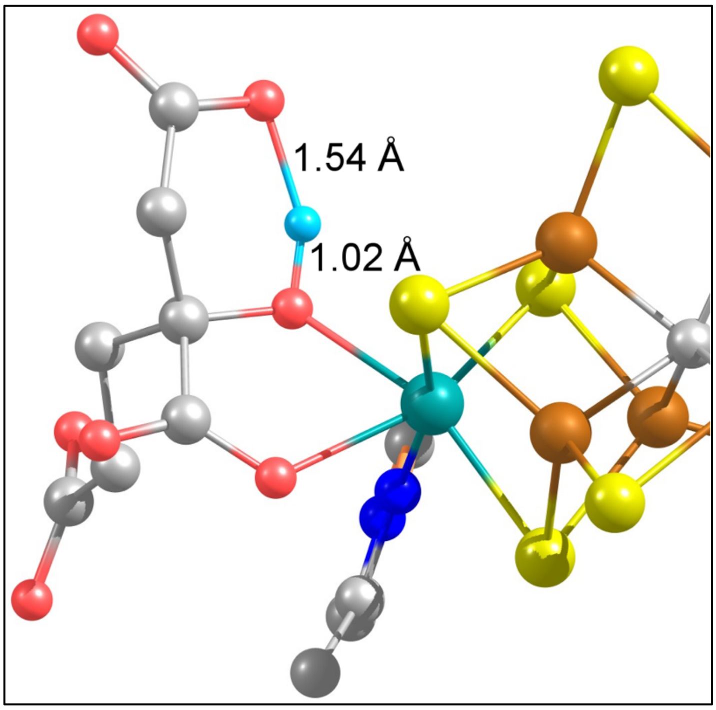

Figure S6.2-1. Bonding and hydrogen bonding distances between the carboxylate and hydroxyl groups of homocitrate.

To investigate the possibility of the hydroxyl group homocitrate undergoing deprotonation to form E_1, we have chosen to investigate several BS solutions of the two most energetically favorable protonation sites (S2B and S5A). Comparing the structural changes which occur at Mo in going from E_0 to a hydroxyl-deprotonated E_1 state (Figure S6.2-2), we find that several significant changes occur. In four of the five cases, a drastic contraction in the Mo-O distance; only the S5A(H) model using a BS247 solution appears reasonable. However, this same solution also displays a drastic increase in the average Mo-S distance, and contraction in the average Mo-Fe distance. The four other solutions show expansion of both the average Mo-S and Mo-Fe distances, well out of the range of experimental error.

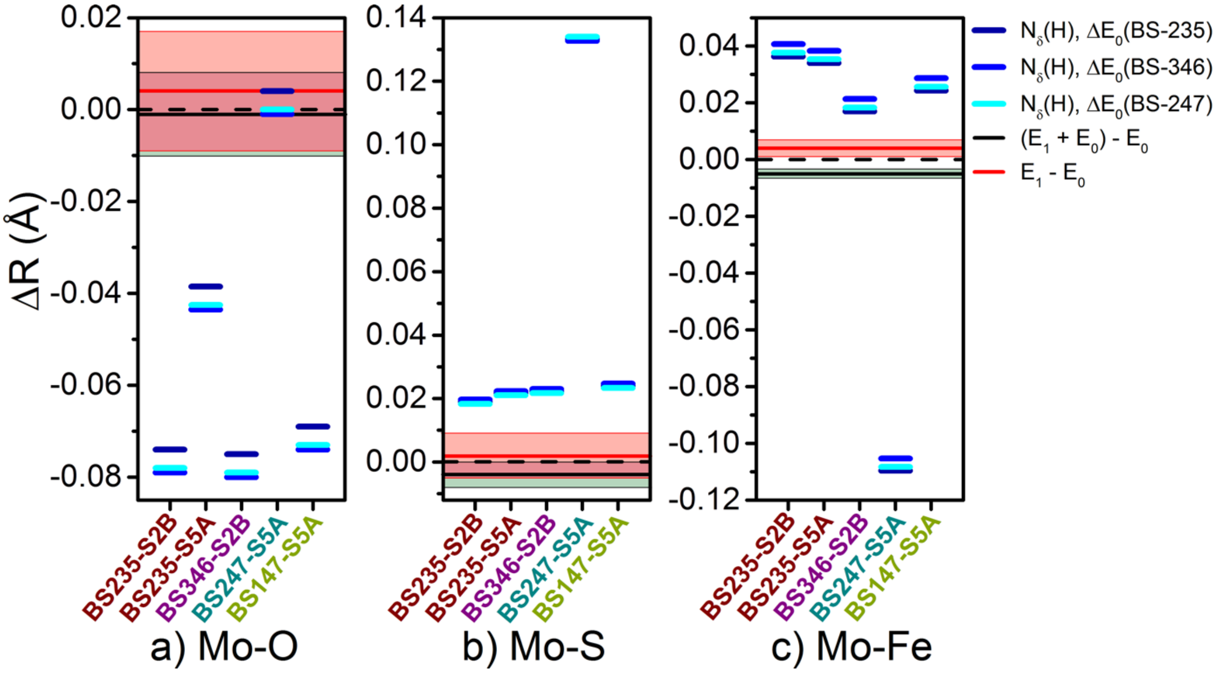

Figure S6.2-2. Comparison of the changes in Mo bond distances for several protonated models of E_1 in which the homocitrate hydroxyl group is unprotonated. Experimental distances and standard errors are provided as solid lines and corresponding shaded areas spanning the width of each graph.

Meanwhile, we find that deprotonation of the hydroxyl group during E_1 formation has very little effect on the average Fe-S, Fe-Fe(short), and Fe-Fe(long) distances (Figure S6.2-3), where the changes observed are similar to those found when the hydroxyl group is protonated. This is not so surprising, as the question of homocitrate protonation state predominately concerns the Mo coordination environment, and only several of the Fe secondarily.

The drastic changes predicted to occur in the Mo coordination environment during formation of E_1 in the absence of a hydroxyl proton are unsupportive of this site being deprotonated during reduction. Furthermore, we find that *all* E_1 models investigated here, which lack a protonated hydroxyl group, produce a series of HOMOs with positive orbital energies (Tables S6.2-1 through S6.2-5). This is an unphysical result, as it implies the occupation of an orbital which is so destabilized that spontaneous ionization would occur. In all cases, only the addition of a H^+ back to the model produces negative energies for all HOMOs.

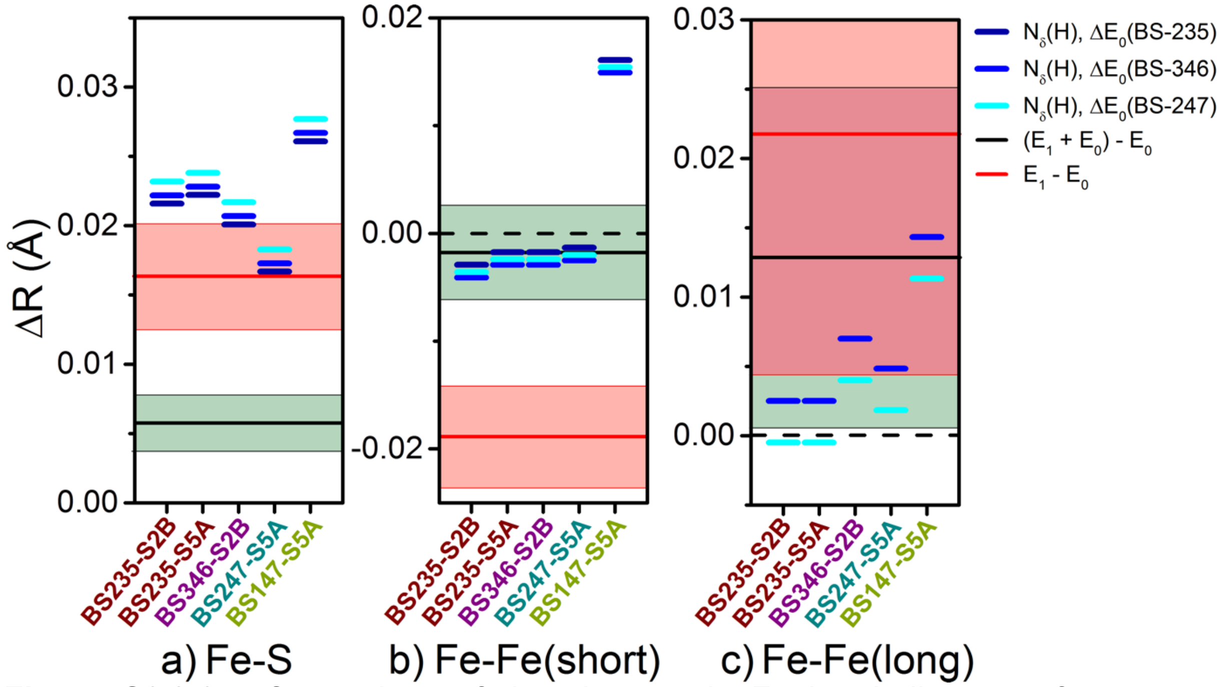

Figure S6.2-3. Comparison of the changes in Fe bond distances for several protonated models of E_1 in which the homocitrate hydroxyl group is unprotonated. Experimental distances and standard errors are provided as solid lines and corresponding shaded areas spanning the width of each graph.

Table S6.2-1. Calculated alpha and beta frontier orbital energies of the unprotonated homocitrate S2B(H) model of E_1, calculated using the BS-235 solution.

α		β	
Orbital	Energy (eV)	Orbital	Energy (eV)
LUMO	2.9501	LUMO	2.9291
HOMO	1.7054	HOMO	1.1761
HOMO-1	1.1893	HOMO-1	1.1051
HOMO-2	0.9646	HOMO-2	1.0225
HOMO-3	0.8322	HOMO-3	0.8506
HOMO-4	0.7821	HOMO-4	0.4976
HOMO-5	0.559	HOMO-5	0.3563
HOMO-6	0.3763	HOMO-6	0.2054
HOMO-7	0.1549	HOMO-7	0.1078
HOMO-8	-0.0394	HOMO-8	0.0105

Table S6.2-2. Calculated alpha and beta frontier orbital energies of the unprotonated homocitrate S5A(H) model of E_1, calculated using the BS-235 solution.

α		β	
Orbital	Energy (eV)	Orbital	Energy (eV)
LUMO	2.9455	LUMO	2.8856
HOMO	1.5229	HOMO	1.1629
HOMO-1	1.2197	HOMO-1	1.1091
HOMO-2	0.9956	HOMO-2	1.0122
HOMO-3	0.8268	HOMO-3	0.783
HOMO-4	0.7857	HOMO-4	0.5739
HOMO-5	0.4991	HOMO-5	0.3986
HOMO-6	0.4591	HOMO-6	0.2641

HOMO-7	0.2027	HOMO-7	0.0965
HOMO-8	0.0428	HOMO-8	0.0424
HOMO-9	-0.0853	HOMO-9	0.0319

Table S6.2-3. Calculated alpha and beta frontier orbital energies of the unprotonated homocitrate S2B(H) model of E_1, calculated using the BS-346 solution.

α		β	
Orbital	Energy (eV)	Orbital	Energy (eV)
LUMO	2.9803	LUMO	2.872
HOMO	1.2957	HOMO	1.2335
HOMO-1	1.1226	HOMO-1	1.1134
HOMO-2	0.887	HOMO-2	1.086
HOMO-3	0.863	HOMO-3	0.8018
HOMO-4	0.6499	HOMO-4	0.5569
HOMO-5	0.4517	HOMO-5	0.48
HOMO-6	0.2931	HOMO-6	0.2543
HOMO-7	0.215	HOMO-7	0.1716
HOMO-8	0.0328	HOMO-8	0.046
HOMO-9	0.0065	HOMO-9	0.0316

Table S6.2-4. Calculated alpha and beta frontier orbital energies of the unprotonated homocitrate S5A(H) model of E_1, calculated using the BS-247 solution.

α		β	
Orbital	Energy (eV)	Orbital	Energy (eV)
LUMO	3.0158	LUMO	2.8436
HOMO	1.2886	HOMO	1.2518
HOMO-1	1.1796	HOMO-1	1.1221
HOMO-2	0.9418	HOMO-2	1.0846
HOMO-3	0.903	HOMO-3	0.7336
HOMO-4	0.8304	HOMO-4	0.5466
HOMO-5	0.4503	HOMO-5	0.5335
HOMO-6	0.4039	HOMO-6	0.3717
HOMO-7	0.1824	HOMO-7	0.1602
HOMO-8	0.1433	HOMO-8	0.1431
HOMO-9	-0.001	HOMO-9	0.1278

Table S6.2-5. Calculated alpha and beta frontier orbital energies of the unprotonated homocitrate S5A(H) model of E_1, calculated using the BS-147 solution.

α		β	
Orbital	Energy (eV)	Orbital	Energy (eV)
LUMO	2.9539	LUMO	2.7726
HOMO	1.5137	HOMO	1.0977
HOMO-1	1.3139	HOMO-1	0.9307
HOMO-2	1.1293	HOMO-2	0.7314
HOMO-3	0.8318	HOMO-3	0.7144
HOMO-4	0.7412	HOMO-4	0.619
HOMO-5	0.6823	HOMO-5	0.4245
HOMO-6	0.289	HOMO-6	0.3209
HOMO-7	0.1403	HOMO-7	0.1898
HOMO-8	0.1223	HOMO-8	0.1227
HOMO-9	-0.0724	HOMO-9	0.0695

S6.3. Protonated models of E_1

S6.3.1 Protonated cluster models of E_1 in a polarizable continuum

To test the influence of the surrounding secondary environment on the energy of belt-sulfide protonated models of E_1, these models were calculated using a simple minimal cluster model of FeMoco with a surrounding polarizable continuum ($\epsilon = 4$) described by CPCM.[17] In these models the MM region and all secondary amino acid residues surrounding FeMoco are absent (homocitrate and the sidechains of His442 and Cys275 included). The resulting relative energies for these models, as well as the Fe-hydride model, is shown in Figure S6.5-1 for the BS-346 electronic state. The relative energies show that removal of the secondary coordination environment makes the bridging sulfides almost equally favorable as protonation sites.

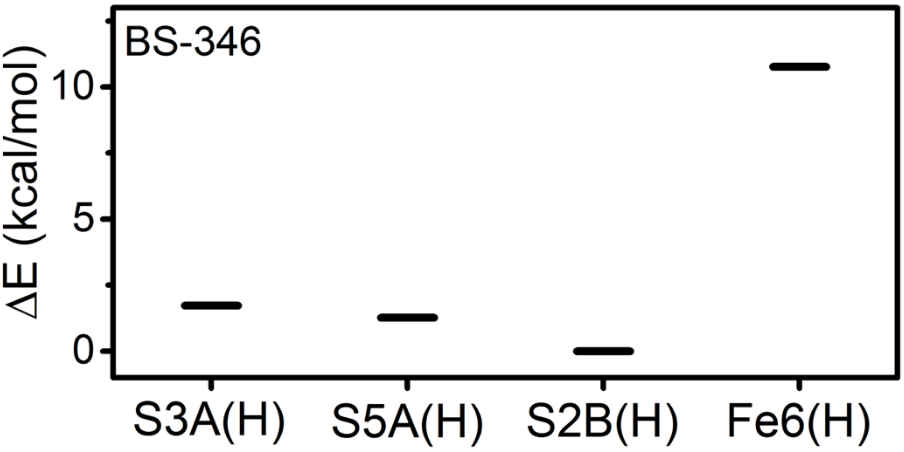

Figure S6.3-1. BS-346 calculated energies (relative to the S2B(H) model) of the belt-sulfide protonated and Fe6 hydride E_1 models in the absence of a secondary coordination sphere.

S6.3.2 Direction of protonation in QM/MM models of E_1

It has been previously found that the direction of protonation of the FeMoco cluster embedded in a QM/MM environment can result in different relative energies. The orientations of protonation selected for each of the models in the present study was partially based on previous studies investigating the favorability of these different orientations for protonation.[18] The protonation of the S5A position was performed such that the proton is directed towards the S3A position. The S3A position was protonated in the direction of S2B. The orientation of the proton on S2B was calculated both in the directions of S5A and S3A for the N_ε-protonated state of His195; the resulting energy difference was found to be 1.4 kcal/mol, in favor of orientation towards the S3A position. Considering 1.4 kcal/mol is smaller than the uncertainty of the calculated energies in our protocol we do not think this orientation dependence is significant and

this has a small effect on the electronic and molecular structure of the cofactor, and given the evidence of H-bonding of His195 to the S2B position in the E_0 state, the S5A oriented model was used in the EXAFS analysis. The S1A position is protonated in an endo-fashion, directed slightly towards the S2B position. Meanwhile, the S3B position is protonated in an exo-fashion nearly trans (161°) from Fe6. Lastly, hydride formation at Fe6 is positioned trans to the central carbide. The SI contains Cartesian coordinates as XYZ files of all models, showing the orientation.

S6.4. Figures & Tables

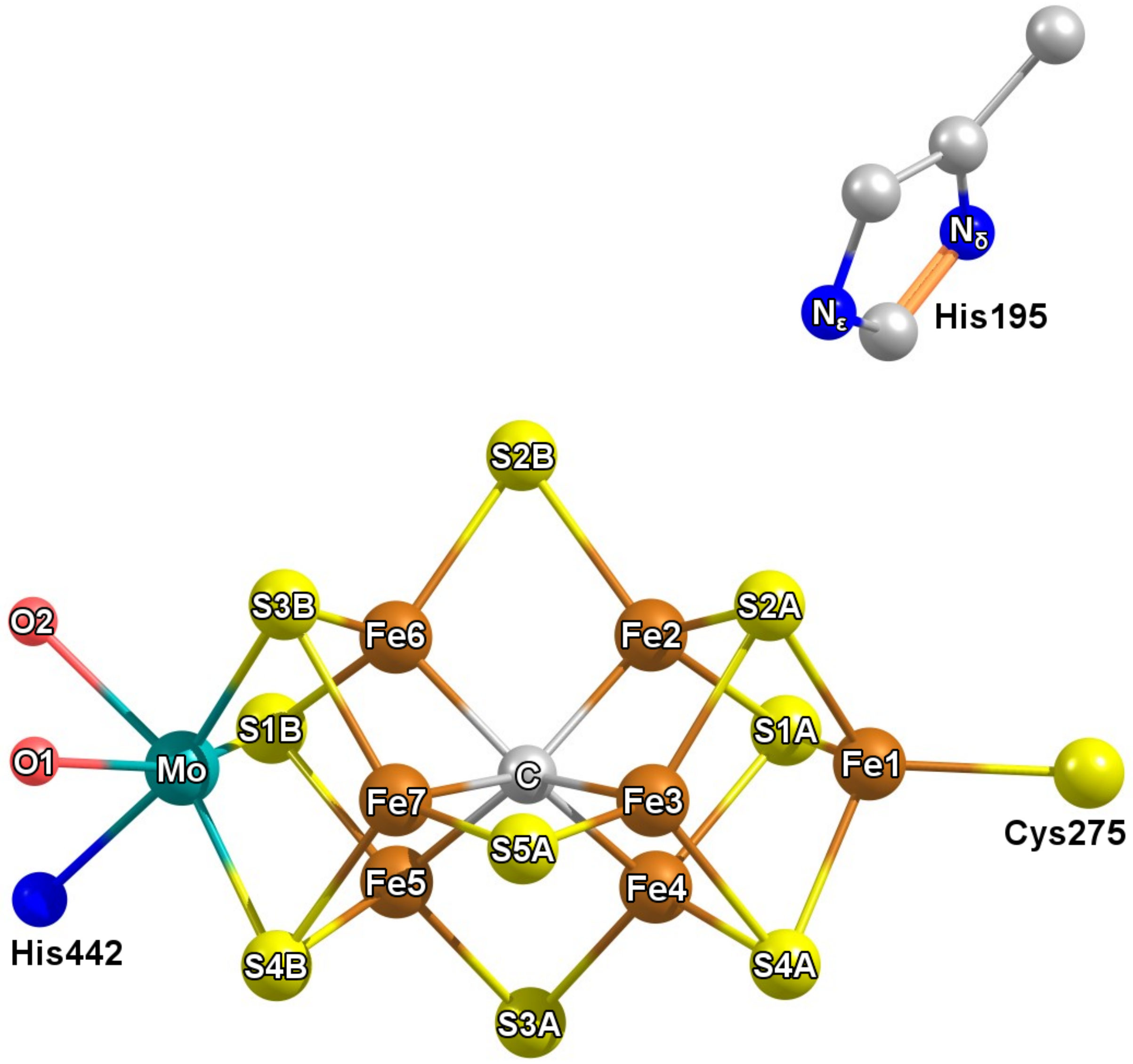

Figure S6.4-1. Enlarged version of the labeling scheme from Figure 1 of the main text.

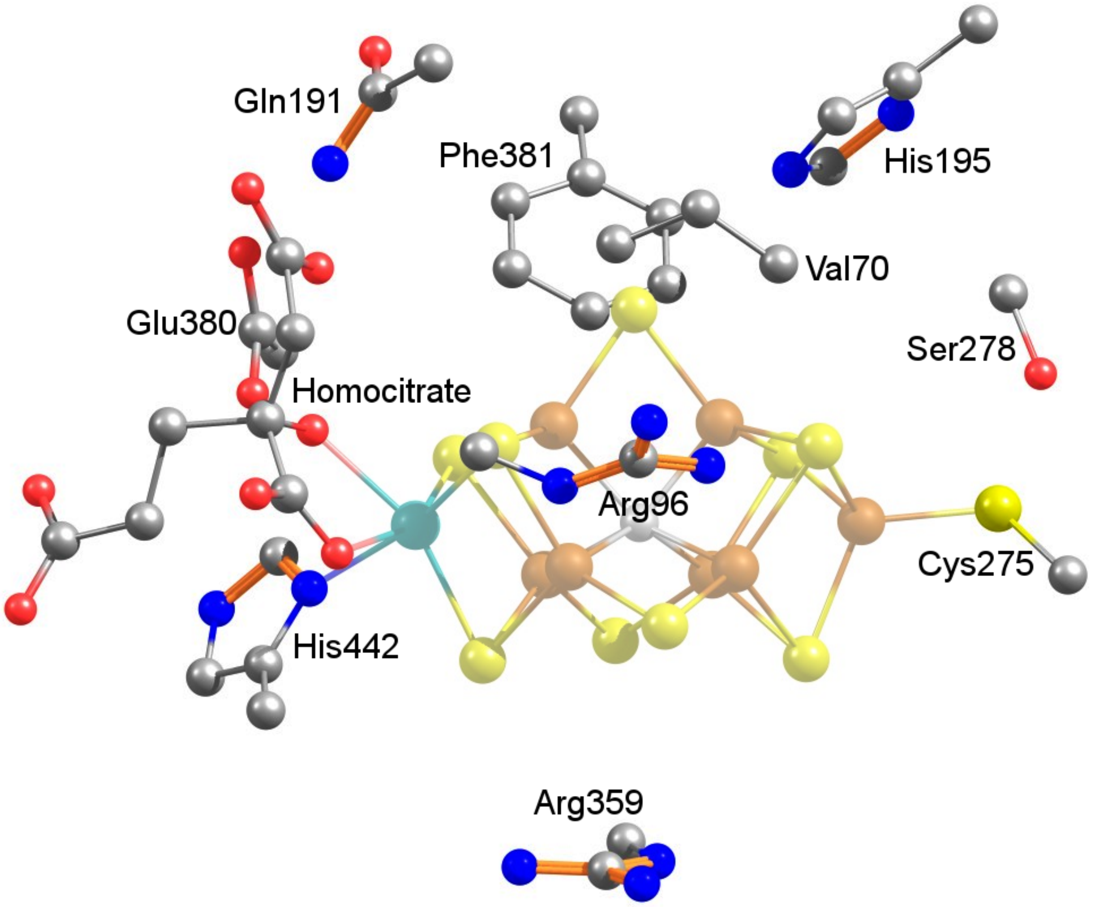

Figure S6.4-2. The QM region used in all QM/MM models for E_0 and E_1 states.

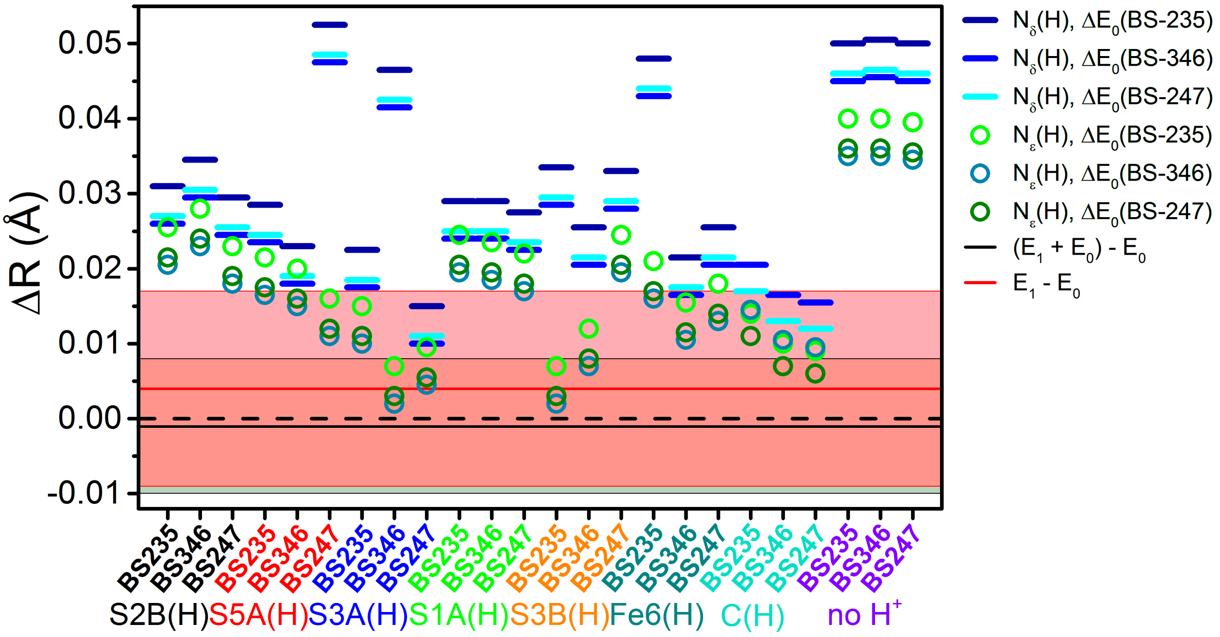

Figure S6.4-3. Average calculated changes in the Mo-O bond distances, ΔR(Mo-O), relative to each BS solution of the E_0 state for all BS solutions of all models and of E_1 in which the homocitrate is protonated. Experimentally determined changes in bond distance from EXAFS and standard errors are provided as solid lines/shaded areas spanning the width of the graph, where **black**/grey denotes the (E_0+E_1) – E_1 , and **red**/pink denotes E_1 – E_0.

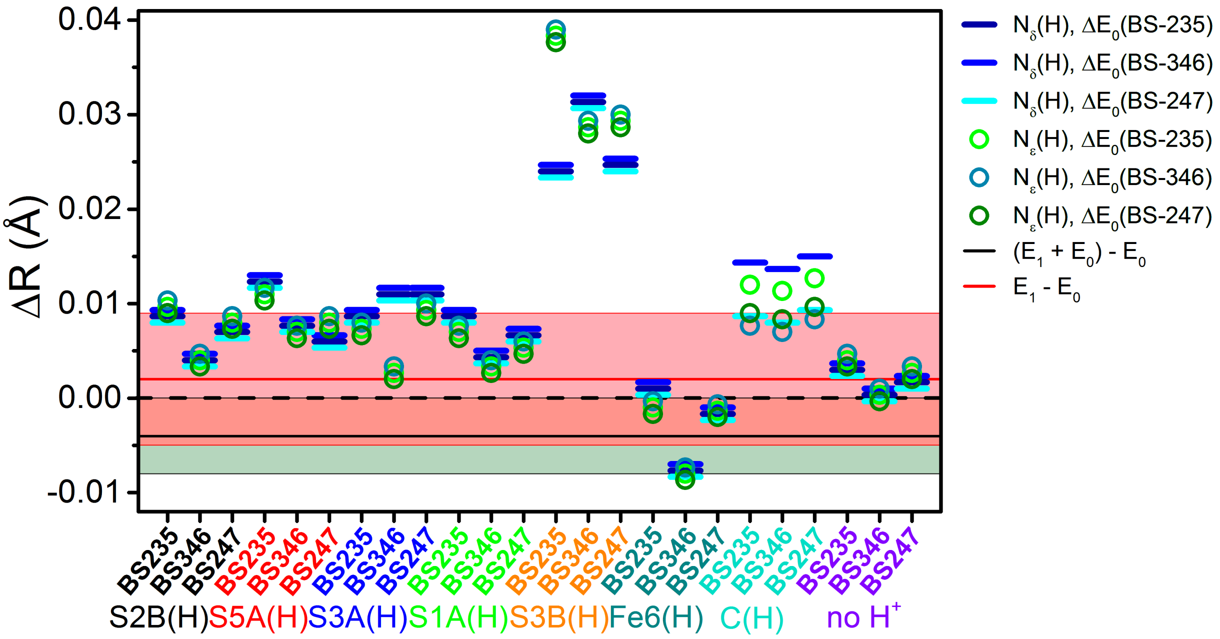

Figure S6.4-4. Average calculated changes in the Mo-S bond distances, ΔR(Mo-S), relative to each BS solution of the E_0 state for all BS solutions of all models and of E_1 in which the homocitrate is protonated. Experimentally determined changes in bond distance from EXAFS and standard errors are provided as solid lines/shaded areas spanning the width of the graph, where **black**/grey denotes the $(E_0+E_1) - E_1$, and **red**/pink denotes $E_1 - E_0$.

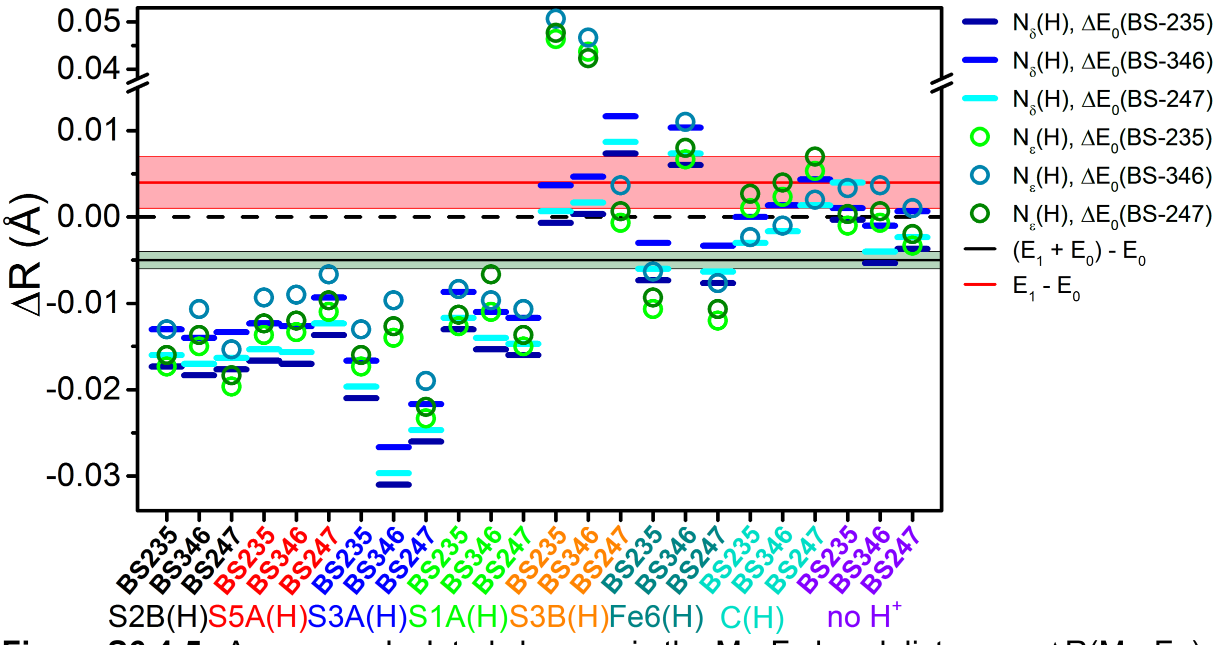

Figure S6.4-5. Average calculated changes in the Mo-Fe bond distances, ΔR(Mo-Fe), relative to each BS solution of the E_0 state for all BS solutions of all models and of E_1 in which the homocitrate is protonated. Experimentally determined changes in bond distance from EXAFS and standard errors are provided as solid lines/shaded areas spanning the width of the graph, where **black**/grey denotes the $(E_0+E_1) - E_1$, and **red**/pink denotes $E_1 - E_0$.

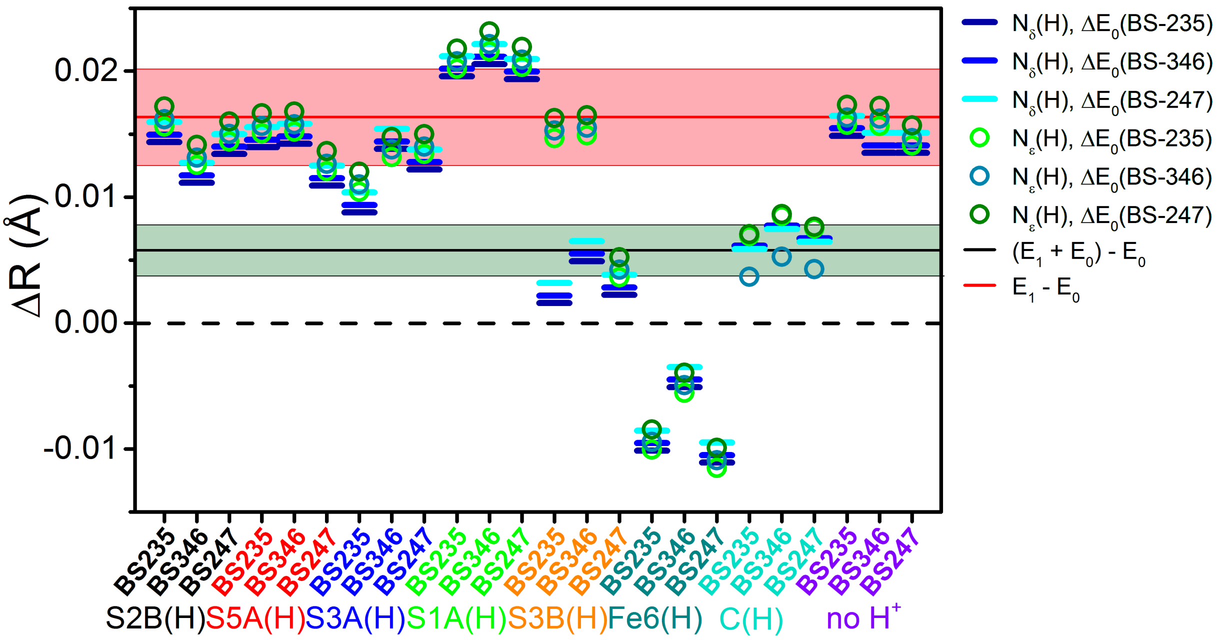

Figure S6.4-6. Average calculated changes in the Fe-S bond distances, ΔR(Fe-S), relative to each BS solution of the E_0 state for all BS solutions of all models and of E_1 in which the homocitrate is protonated. Experimentally determined changes in bond distance from EXAFS and standard errors are provided as solid lines/shaded areas spanning the width of the graph, where **black**/grey denotes the $(E_0+E_1) - E_1$, and **red**/pink denotes $E_1 - E_0$.

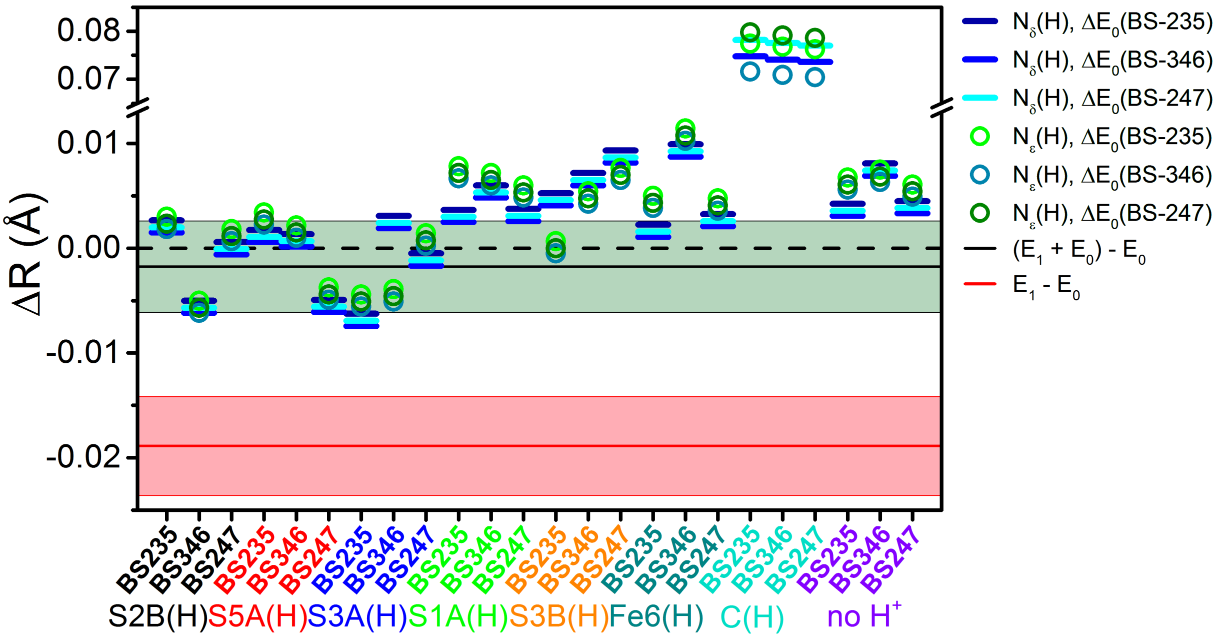

Figure S6.4-7. Average calculated changes in the Fe-Fe(s) bond distances, ΔR(Fe-Fe(s)), relative to each BS solution of the E_0 state for all BS solutions of all models and of E_1 in which the homocitrate is protonated. Experimentally determined changes in the Fe-Fe (short 1) scattering distance from EXAFS and standard errors are provided as solid lines/shaded areas spanning the width of the graph, where **black**/grey denotes the $(E_0+E_1) - E_1$, and **red**/pink denotes $E_1 - E_0$.

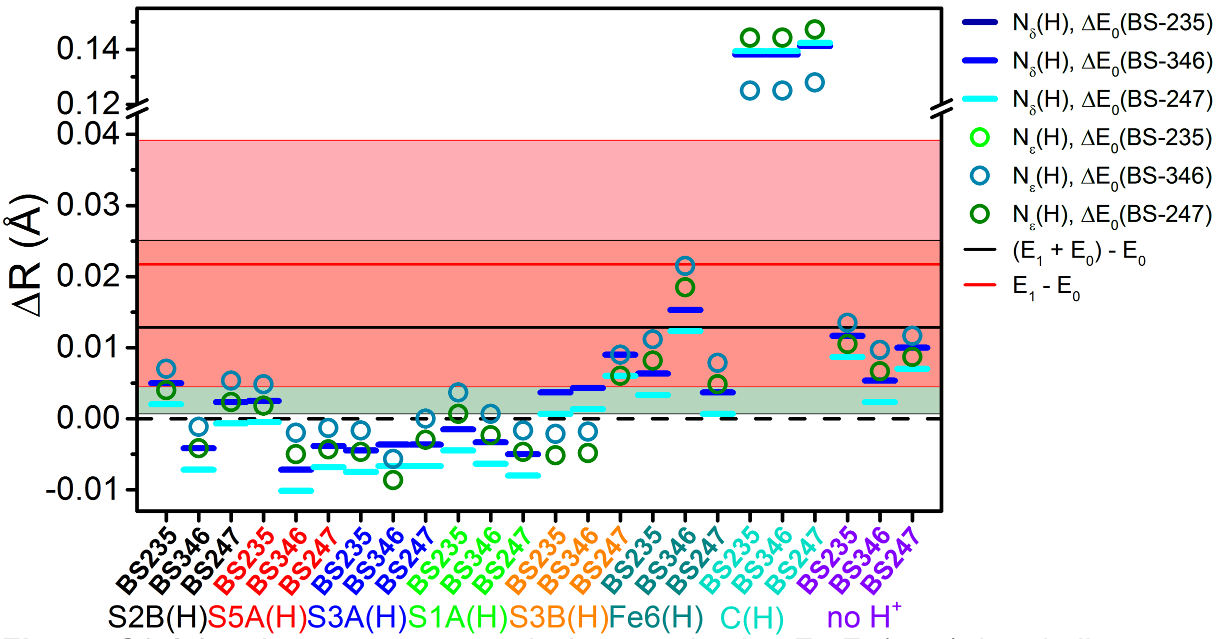

Figure S6.4-8. Average calculated changes in the Fe-Fe(long) bond distances, ΔR(Fe-Fe(l)), relative to each BS solution of the E_0 state for all BS solutions of all models and of E_1 in which the homocitrate is protonated. Experimentally determined changes in the Fe-Fe(long) scattering distance from EXAFS and standard errors are provided as solid lines/shaded areas spanning the width of the graph, where **black**/grey denotes the $(E_0+E_1) - E_1$, and **red**/pink denotes $E_1 - E_0$. Since the BS-235 and BS-247 solutions of the E_0 state produce an identical average Fe-Fe(long) distance, all ΔE_0(BS-235) and ΔE_0(BS-247) changes in distance appear identical for a given protonation model/BS state/His195 protonation state combination.

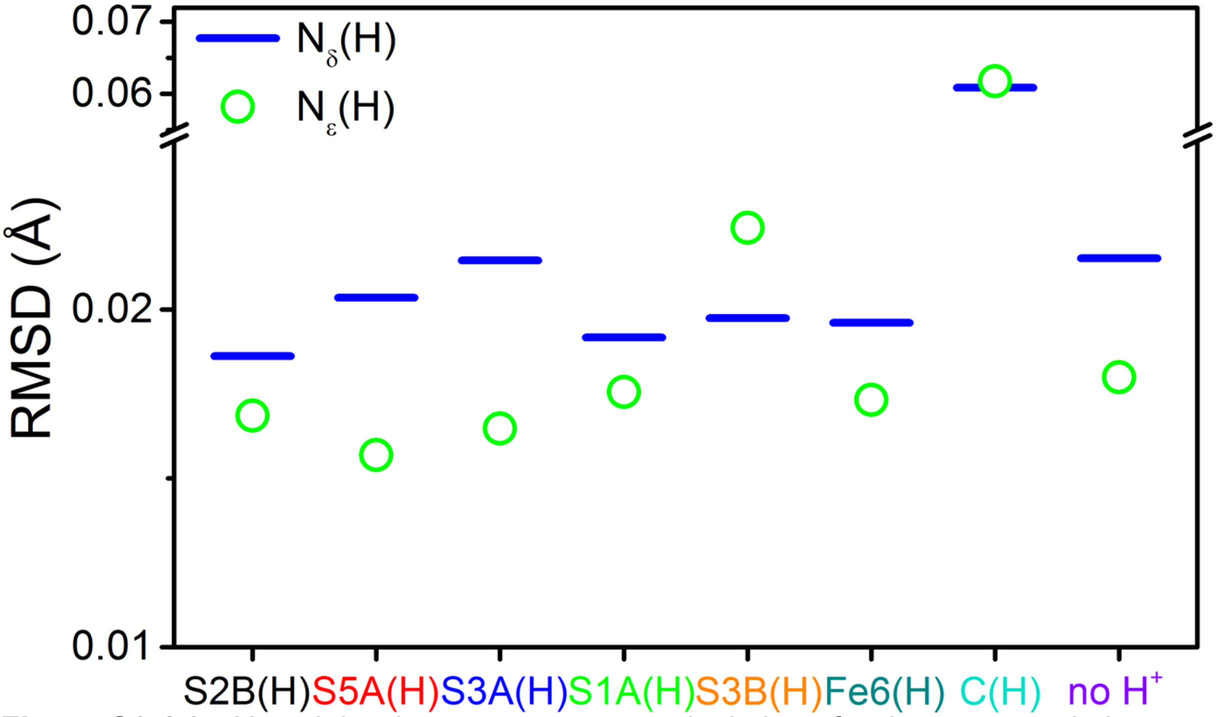

Figure S6.4-9. Unweighted root-mean-square deviations for the calculated changes in distances (ΔR^{calc}) of the computational models of the E_1 state averaged across the BS-235, BS-346, and BS-247 solutions. Calculation of the unweighted RMSDs is described in the Statistical Analysis section of the Experimental details. Experimental changes in distances (ΔR^{exp}) used in these calculations were acquired from E_1-E_0.

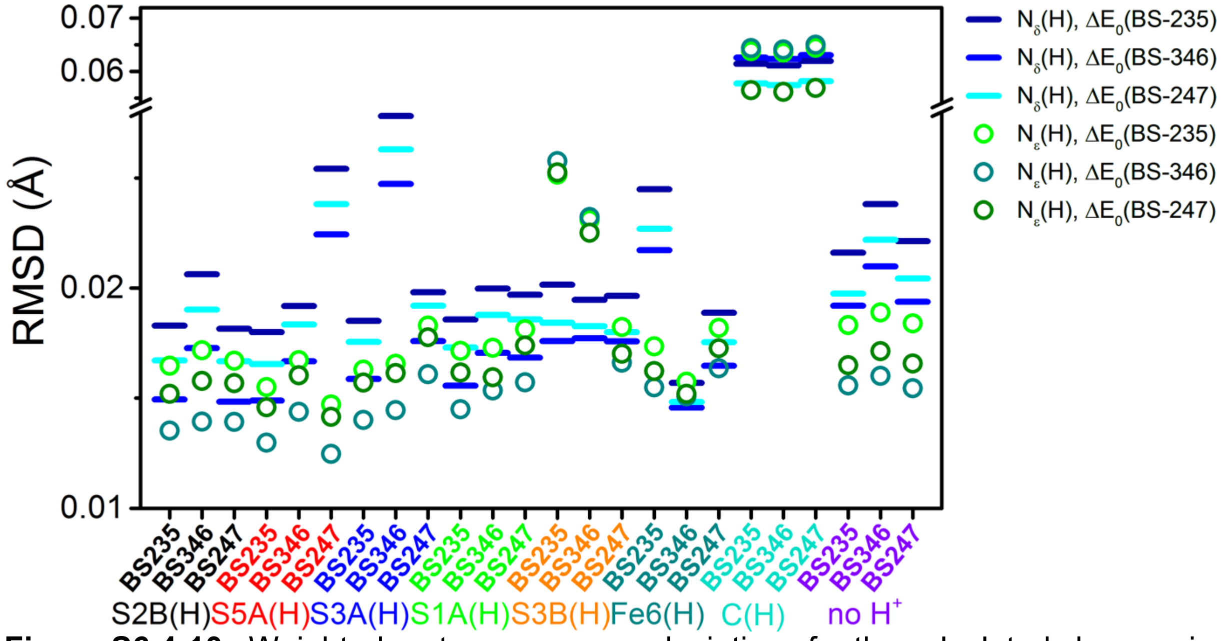

Figure S6.4-10. Weighted root-mean-square deviations for the calculated changes in distances (ΔR^{calc}) of the computational models of the E_1 state. Each individual contributing path has been weighted based on the experimental standard deviation, as described in the Statistical Analysis section of the Experimental details. Experimental changes in distances (ΔR^{exp}) used in these calculations were acquired from E_1-E_0.

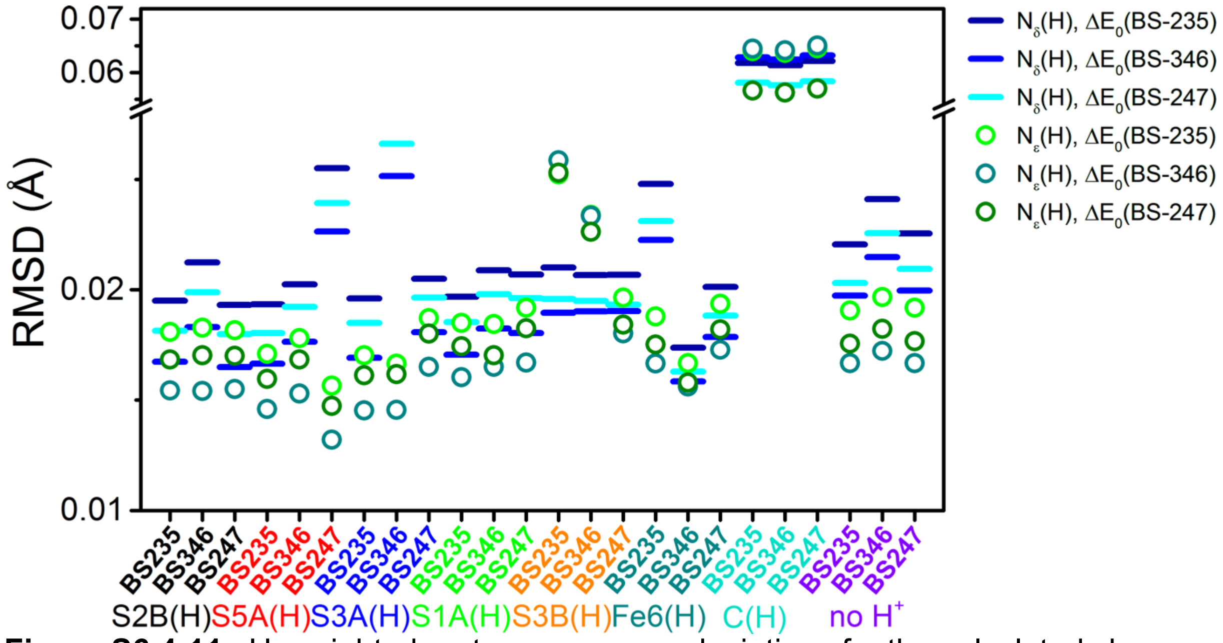

Figure S6.4-11. Unweighted root-mean-square deviations for the calculated changes in distances (ΔR^{calc}) of the computational models of the E_1 state. Calculation of the unweighted RMSDs is described in the Statistical Analysis section of the Experimental details. Experimental changes in distances (ΔR^{exp}) used in these calculations were acquired from E_1-E_0.

Table S6.4-1. Compilation of the calculated relative energies (both polarized QM and QM/MM energies are shown) for the various protonation models investigated, for which the N_δ position of His195 is protonated.

	ΔE (kcal/mol)

	BS346		BS235		BS247	
Model	QM	QM/MM	QM	QM/MM	QM	QM/MM
S2B(H)	0	0	0.9	1.5	0.8	1.7
S3A(H)	17.6	17.7	15.4	16.8	17.8	18.6
S5A(H)	9.1	6.8	7.3	8.8	8.2	6.1
S1A(H)	21.9	20.1	21.0	19.9	19.4	18.1
S3B(H)	33.3	27.7	29.3	23.9	33.9	29.4
Fe6(H)	16.6	13.8	17.6	16.0	18.7	16.4

Table S6.4-2. Compilation of the calculated relative energies (both polarized QM and QM/MM energies are shown) for the various protonation models investigated, for which the N_ε position of His195 is protonated.

	ΔE (kcal/mol)					
	BS346		BS235		BS247	
Model	QM	QM/MM	QM	QM/MM	QM	QM/MM
S2B(H)	0.4	0.0	1.2	1.3	1.4	2.1
S3A(H)	9.8	12.6	8.0	10.6	9.3	12.6
S5A(H)	0.0	2.6	1.2	3.6	2.5	1.1
S1A(H)	14.7	14.4	15.2	15.2	12.5	13.1
S3B(H)	21.5	23.3	18.3	21.7	26.3	26.6
Fe6(H)	10.8	10.4	14.0	12.8	14.2	13.3

Table S6.4-3. Calculated alpha and beta frontier orbital energies of the BS-235 model of E_1 without proton transfer, N_δ(H) protonation state of His195.

α		β	
Orbital	Energy (eV)	Orbital	Energy (eV)
LUMO	3.6474	LUMO	3.6861
HOMO	2.0627	HOMO	1.8777
HOMO-1	1.9289	HOMO-1	1.8305
HOMO-2	1.5973	HOMO-2	1.73
HOMO-3	1.5712	HOMO-3	1.3393
HOMO-4	1.3939	HOMO-4	1.2155
HOMO-5	1.1767	HOMO-5	1.1357
HOMO-6	1.1194	HOMO-6	0.9645
HOMO-7	0.9782	HOMO-7	0.766
HOMO-8	0.7133	HOMO-8	0.7227
HOMO-9	0.4961	HOMO-9	0.5084
HOMO-10	0.44	HOMO-10	0.4371
HOMO-11	0.2834	HOMO-11	0.2211
HOMO-12	0.1977	HOMO-12	0.1351
HOMO-13	0.1304	HOMO-13	0.0691

Table S6.4-4. Calculated alpha and beta frontier orbital energies of the BS-346 model of E_1 without proton transfer, N_δ(H) protonation state of His195.

α		β	
Orbital	Energy (eV)	Orbital	Energy (eV)
LUMO	3.6672	LUMO	3.5565
HOMO	2.0701	HOMO	1.749
HOMO-1	1.6714	HOMO-1	1.7031
HOMO-2	1.5545	HOMO-2	1.6464
HOMO-3	1.4308	HOMO-3	1.3186

HOMO-4	1.3319	HOMO-4	1.2398
HOMO-5	1.1923	HOMO-5	1.1252
HOMO-6	0.9536	HOMO-6	0.831
HOMO-7	0.7096	HOMO-7	0.7817
HOMO-8	0.5939	HOMO-8	0.7227
HOMO-9	0.4901	HOMO-9	0.4641
HOMO-10	0.4493	HOMO-10	0.295
HOMO-11	0.192	HOMO-11	0.0838
HOMO-12	0.0182	HOMO-12	0.0381
HOMO-13	-0.0644	HOMO-13	0.004

Table S6.4-5. Calculated alpha and beta frontier orbital energies of the BS-247 model of E_1 without proton transfer, N_δ(H) protonation state of His195.

α		β	
Orbital	Energy (eV)	Orbital	Energy (eV)
LUMO	3.6417	LUMO	3.6004
HOMO	2.0671	HOMO	1.8791
HOMO-1	1.8538	HOMO-1	1.8347
HOMO-2	1.6369	HOMO-2	1.7418
HOMO-3	1.5682	HOMO-3	1.3764
HOMO-4	1.3946	HOMO-4	1.26
HOMO-5	1.2657	HOMO-5	1.2181
HOMO-6	1.0275	HOMO-6	0.967
HOMO-7	0.9775	HOMO-7	0.9027
HOMO-8	0.6587	HOMO-8	0.771
HOMO-9	0.4693	HOMO-9	0.5125
HOMO-10	0.449	HOMO-10	0.4669
HOMO-11	0.3305	HOMO-11	0.2664
HOMO-12	0.2533	HOMO-12	0.1371
HOMO-13	0.0961	HOMO-13	0.0849

Table S6.4-6. Calculated alpha and beta frontier orbital energies of the BS-235 model of E_1 without proton transfer, N_ε(H) protonation state of His195.

α		β	
Orbital	Energy (eV)	Orbital	Energy (eV)
LUMO	3.3784	LUMO	3.4665
HOMO	1.8293	HOMO	1.6251
HOMO-1	1.6833	HOMO-1	1.576
HOMO-2	1.3538	HOMO-2	1.503
HOMO-3	1.2951	HOMO-3	1.1571
HOMO-4	1.1887	HOMO-4	0.9507
HOMO-5	0.9381	HOMO-5	0.8984
HOMO-6	0.8804	HOMO-6	0.7431
HOMO-7	0.7317	HOMO-7	0.5482
HOMO-8	0.4683	HOMO-8	0.4846
HOMO-9	0.3096	HOMO-9	0.2489
HOMO-10	0.2182	HOMO-10	0.2016
HOMO-11	0.0784	HOMO-11	-0.029

References

1. S. Stoll and A. Schweiger, *J. Magn. Reson.*, 2006, **178**, 42-55.
2. L. Zhang, J. T. Kaiser, G. Meloni, K.-Y. Yang, T. Spatzal, S. L. A. Andrade, O. Einsle, J. B. Howard and D. C. Rees, *Angew. Chem., Int. Ed. Engl.*, 2013, **52**, 10529-10532.
3. T. Spatzal, M. Aksoyoglu, L. Zhang, S. L. Andrade, E. Schleicher, S. Weber, D. C. Rees and O. Einsle, *Science*, 2011, **334**, 940.
4. K. M. Lancaster, Y. Hu, U. Bergmann, M. W. Ribbe and S. DeBeer, *J. Am. Chem. Soc.*, 2013, **135**, 610-612.
5. K. M. Lancaster, M. Roemelt, P. Ettenhuber, Y. Hu, M. W. Ribbe, F. Neese, U. Bergmann and S. DeBeer, *Science*, 2011, **334**, 974-977.
6. D. Lukoyanov, V. Pelmenschikov, N. Maeser, M. Laryukhin, T. C. Yang, L. Noodleman, D. R. Dean, D. A. Case, L. C. Seefeldt and B. M. Hoffman, *Inorg. Chem.*, 2007, **46**, 11437-11449.
7. T. E. Westre, P. Kennepohl, J. G. DeWitt, B. Hedman, K. O. Hodgson and E. I. Solomon, *J. Am. Chem. Soc.*, 1997, **119**, 6297-6314.
8. G. R. Shulman, Y. Yafet, P. Eisenberger and W. E. Blumberg, *Proc. Nat. Acad. Sci.*, 1976, **73**, 1384-1388.
9. S. Yao, F. Meier, N. Lindenmaier, R. Rudolph, B. Blom, M. Adelhardt, J. Sutter, S. Mebs, M. Haumann, K. Meyer, M. Kaupp and M. Driess, *Angew. Chem., Int. Ed. Engl.*, 2015, **54**, 12506-12510.
10. B. Benediktsson and R. Bjornsson, *Inorg. Chem.*, 2017, **56**, 13417-13429.
11. R. Bjornsson, F. Neese and S. DeBeer, *Inorg. Chem.*, 2017, **56**, 1470-1477.
12. R. Bjornsson, F. A. Lima, T. Spatzal, T. Weyhermüller, P. Glatzel, E. Bill, O. Einsle, F. Neese and S. DeBeer, *Chem. Sci.*, 2014, **5**, 3096-3103.
13. S. J. Yoo, H. C. Angove, V. Papaefthymiou, B. K. Burgess and E. Münck, *J. Am. Chem. Soc.*, 2000, **122**, 4926-4936.
14. T. Lovell, J. Li, T. Liu, D. A. Case and L. Noodleman, *J. Am. Chem. Soc.*, 2001, **123**, 12392-12410.
15. J. Pipek and P. G. Mezey, *J. Chem. Phys.*, 1989, **90**, 4916-4926.
16. L. Cao, O. Caldararu and U. Ryde, *J. Phys. Chem. B*, 2017, **121**, 8242-8262.
17. V. Barone and M. Cossi, *J. Phys. Chem. A*, 1998, **102**, 1995-2001.
18. L. Cao, O. Caldararu and U. Ryde, *J. Chem. Theory Comput.* 2018, **14**, 6653-6678.
